# What works to reduce socioeconomic inequalities in hospitalisations and readmissions? Systematic review of the equity impacts of population-level, health service and integrative interventions

**DOI:** 10.1136/bmjph-2025-002595

**Published:** 2025-09-23

**Authors:** Sarah Sowden, Behrouz Nezafat Maldonado, Fiona Beyer, William Bell, Mark Lambert, Richard Thomson, Richard Cookson, Clare Bambra

**Affiliations:** 1 Population Health Sciences Institute, Newcastle University, Newcastle upon Tyne, UK; 2 Imperial College London, London, UK; 3 NHS England, Newcastle upon Tyne, UK; 4 Centre for Health Economics, University of York, York, UK

**Keywords:** Preventive Medicine, Public Health, Sociodemographic Factors, Systematic Review

## Abstract

Inequalities exist in hospitalisation rates, which are undesirable and costly for health systems; with higher rates in populations with lower levels of income, education or residing in socioeconomically marginalised neighbourhoods where ill-health is more prevalent and preventive care is more limited.

**Objectives:**

To understand which interventions reduce, maintain or increase socioeconomic inequalities in hospitalisations or readmissions to aid efforts of policymakers and practitioners working to improve health equity and reduce hospital pressures.

**Design:**

Systematic review.

**Eligibility criteria:**

Intervention studies in any Organisation for Economic Co-operation and Development (OECD) country, involving individuals of any age, published in any language which reported the differential impact across socioeconomic group (any classification) for three categories of intervention (population-level, health service or integrative interventions) on hospitalisation or readmission outcomes (all cause or condition specific).

**Data extraction and synthesis:**

An electronic search of MEDLINE, Embase, CINAHL, Cochrane CENTRAL and Web of Knowledge was conducted covering 24 years (from 1 January 2000 to 1 April 2024), supplemented with full citation searches of included studies, website searches and expert consultation. Risk of bias was assessed using the EHPP tool, direction of effect classified and narrative synthesis conducted.

**Results:**

From 25 618 records screened, 36 studies met the inclusion criteria, conducted in eight countries with 42% of these published in the past 5 years. Studies employed a range of study designs and 88% were rated as either moderate or strong quality. A range of equity impacts of interventions on hospitalisations and readmissions were observed; 6 interventions increased inequalities, 7 maintained, 10 had mixed or inconclusive impacts, and 13 studies reported effective interventions for reducing inequalities. Interventions successful at reducing inequalities were those implemented and enforced across entire populations and systems and supportive interventions tailored to the varied needs and contexts of people from different socioeconomic groups.

**Conclusions:**

Socioeconomic disadvantage was variously measured making comparison of equity impacts across studies complex. Policymakers and practitioners cannot assume that interventions implemented to reduce hospitalisations or readmissions will necessarily reduce prevailing and costly healthcare inequalities; it is imperative that the equity impacts of interventions are consistently monitored. To improve equity of hospital outcomes, investment in population health and integrative activity addressing the social determinants of health, alongside health service interventions, is required.

**PROSPERO registration number:**

CRD42019153666.

**WHAT IS ALREADY KNOWN ON THIS TOPIC**

- Stark inequalities exist in hospitalisations but the differential impact across socioeconomic groups of interventions to reduce hospitalisations has not been systematically assessed, nor has the impact of non-health care interventions on inequalities in hospitalisations.

**WHAT THIS STUDY ADDS**

- Interventions were identified that reduce, maintain and increase socioeconomic inequalities in hospitalisations. Interventions successful at reducing inequalities include those implemented across entire populations or health systems, and supportive interventions tailored to the varied needs and contexts of people from different socioeconomic groups.

**HOW THIS STUDY MIGHT AFFECT RESEARCH, PRACTICE OR POLICY**

- Interventions to reduce hospitalisations can exacerbate socioeconomic inequalities, so it is imperative that equity impacts are consistently monitored. Cross-sector investment is required to reduce inequalities in hospitalisations as health-service interventions alone will fail to do this.

## Introduction

 Stark inequalities exist in avoidable hospitalisation^1^,^3^; with higher rates in those who are unemployed or with lower levels of income, those with lower education or literacy, those living in socioeconomically marginalised neighbourhoods and in the USA, those in receipt of Medicaid.^4^ Hospitalisations and readmissions are costly and undesirable for healthcare systems and reducing these is a focus of healthcare policy in the UK^5^ and worldwide^1^ as is addressing health and care inequalities.^6^,^9^ Avoidable hospitalisations account for over 37 million bed days each year across the European Union^10^ and excess hospitalisations associated with socioeconomic inequality have previously been estimated to cost the national health service in England £4.8 billion per year.^11^ Alongside being costly to health services, inequalities in hospitalisations are also costly to the families and individuals impacted^11^ and to wider society through, for example, loss of earnings and economic productivity.

There are varied definitions used to describe and measure hospitalisations that can be avoided or prevented.^1^ In this research, we are concerned with socioeconomic inequalities in any hospitalisation that can be prevented through intervention, be that through primary preventative measures (eg, wearing seatbelts to prevent admissions resulting from severe injury in road traffic accidents or fluoridating water to prevent tooth decay in children and resultant hospital admissions for dental extractions) or through secondary and tertiary prevention measures (eg, improving ambulatory care in the community for patients with angina to prevent them being admitted to hospital due to an escalation in their condition).

Existing reviews have documented the pervasive presence of socioeconomic inequalities in avoidable hospitalisations^1^,^3^ but not how to intervene to address them.^12^ There is review-level evidence of promising interventions to reduce avoidable hospital admissions in areas including education, self-management, rehabilitation and telemedicine, but previous reviews have focused principally on health service interventions^1 13^ and report only on the *average* effect of interventions on hospitalisations^1 13^ and readmissions,^14^ without examining whether there is a *differential* impact of interventions across people from different socioeconomic groups. This is needed to make an assessment of the impact of interventions on inequalities in hospitalisations.

**Figure 1:**
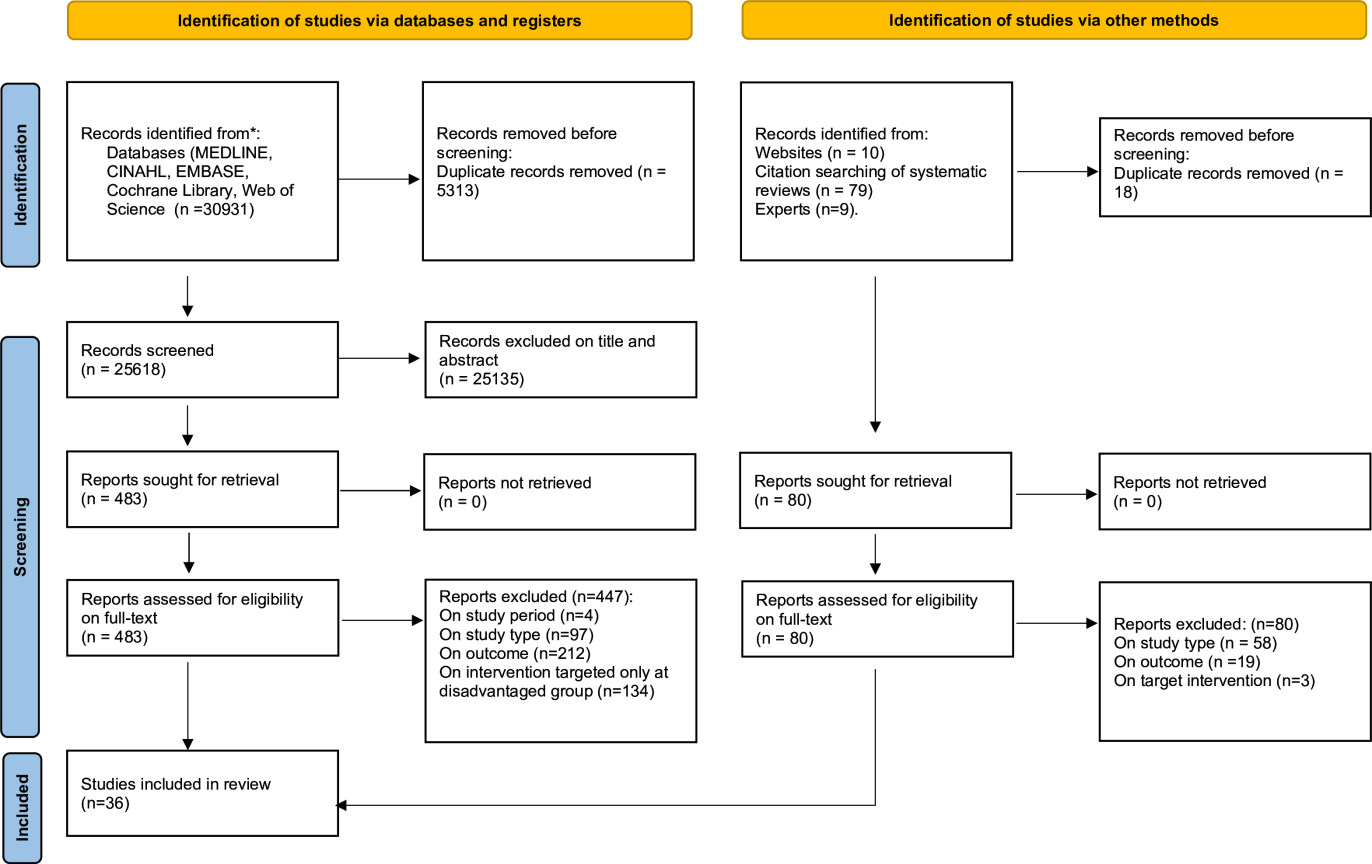
PRISMA flow diagram. PRISMA, Preferred Reporting Items for Systematic Reviews and Meta-Analysis.

It is important to examine the impact of interventions on inequalities because well-intended interventions can increase as well as decrease inequalities, a phenomenon known as intervention generated inequalities.^9 15^ In consultation with experts and members of the public, we have previously developed a theoretical framework to articulate the mechanisms through which interventions may influence inequalities in hospitalisations and readmissions (see figure 1, theoretical framework, outlined in the review protocol).^1^ Mechanisms include, for example, implementing an intervention universally (for example, a smoking ban in public places) or offering a service across an entire population (universal child vaccination programme), but providing additional targeted elements of service provision in neighbourhoods of socioeconomic disadvantage (for example, out-reach clinics in community venues offering flexible vaccination appointment times).

A limited number of review studies have examined approaches health services can take to intervene to address health inequalities^16^ and improve healthcare equity,^17^ but these are not focused on the outcome of hospitalisation or readmissions. There is also established evidence demonstrating the importance of public policy intervention on the wider determinants of health to effectively address health inequalities more broadly,^9^ but this literature has not been systematically considered with respect to inequalities in hospitalisations or readmissions.

This systematic review, therefore, includes a full range of interventions, using a categorisation that we previously developed and reported in the review protocol,^1^ informed by members of the public, an expert panel and previous literature:

1. *Health service interventions*: disease, medication and case management interventions (eg, education programmes, structured discharge planning, comprehensive geriatric assessment, virtual wards, hospital at home initiatives,^13^ vaccination programmes as well as system-wide care (provision, access, quality, continuity) benchmarking and intervention.
2. *Integrative interventions*: designed to create greater synergy between primary and secondary healthcare and community care and non-healthcare organisations and operations.^18 19^ For example, social prescribing initiatives facilitate closer working between healthcare and voluntary and statutory organisations supporting the wider determinants of health (housing, employment, education and social cohesion).
3. *Population health and public policy interventions*: legal, fiscal, structural, organisational, environmental and policy interventions that seek to change health-related behaviours or to modify the social and economic determinants of health.^20^

This systematic review aimed to examine the differential impact of interventions across socioeconomic groups in order to understand which health service, integrative and population health and public policy interventions reduce, maintain or increase socioeconomic inequalities in hospitalisations or readmissions. This is to aid efforts of policymakers and practitioners working to improve health equity and reduce costly hospital pressures.

## Methods

The review used the Preferred Reporting Items for Systematic Reviews and Meta-Analysis with a focus on health equity (PRISMA-E),^21^ and Synthesis without meta-analysis in systematic reviews (SWiM)^22^ reporting guidelines (online supplemental file 1). The review was registered with PROSPERO (CRD42019153666), and the protocol was published in a peer-reviewed journal.^1^

An electronic search of MEDLINE (Ovid), Embase (Ovid), CINAHL (EBSCO), Cochrane CENTRAL (Wiley), Science Citation Index (Web of Knowledge), Social Science Citation Index (Web of Knowledge) was conducted covering the time period 1 January 2000 to 1 April 2024, supplemented with full citation searches of included studies, website searches (Health Foundation, Nuffield Trust, Organisation for Economic Co-operation and Development (OECD), WHO, EuroStat, King’s Fund) for relevant grey literature and expert consultation with the Understanding Factors that explain Avoidable hospital admission Inequalities Research study (UNFAIR) programme collaborators.^23^ Our search was designed to focus on the impact of interventions on inequalities in hospitalisations. The electronic search combined search terms around intervention evaluation (eg, terms such as programme, policy, strategy and initiative close to terms such as evaluate, measure, assess, impact) with hospitalisations (eg, hospitalisation, patient readmission, patient admission) and socioeconomic characteristics using a validated health equity electronic search filter^24^ (eg, occupation, income, education, deprivation, home ownership, social class, poverty, socioeconomic factors). The complete search strategies for electronic databases are provided in online supplemental file 2.

Quantitative investigations of intervention effect of any study design, involving individuals of any age, undertaken in OECD countries, published in any language were included (see protocol for full inclusion and exclusion criteria).^1^ Studies were included if the differential effect of an intervention according to any measure of socioeconomic status (education, income, occupation, social class, deprivation, poverty, medicaid receipt status or an area-based characterisation of deprivation based on place of residence) with respect to all-cause or condition-specific hospitalisation or readmission was reported. Any studies reporting a composite outcome (eg, hospitalisation and deaths combined) were excluded.

All articles identified through the search were uploaded into Eppi-reviewer V.4 software^25^ and duplicates were removed automatically and manually. Two reviewers (SS and BNM) screened titles and abstracts of the retrieved articles to assess eligibility for inclusion (10% of papers were double screened independently to check concordance). Full-text screening of all potentially eligible articles was conducted independently by two reviewers (SS and BNM). Any disagreements at any stage were resolved through consultation with a third reviewer (FB).

Risk of bias was assessed independently by reviewers (SS, BNM and WB) using the Effective Public Health Practice Project (EPHPP) tool,^26^ chosen because the same tool can be used across a range of quantitative study designs. Data for included studies were extracted by three reviewers (SS, BNM and WB). Meta-analysis was not feasible due to variability in how socioeconomic status was defined, categorised and the differential equity outcome was reported, heterogeneity in study designs, population characteristics and outcomes were assessed. Instead, narrative synthesis of studies was conducted, including vote counting based on direction of effect,^27^ which was summarised in a modified version of a harvest plot^28^ (reported according to the PRISMA-E)^21^ checklist and SWiM guideline^22^—online supplemental file 1).

In a change to the protocol, the community domain was amalgamated with the integrative domain because in practice these two domains were mutually inclusive. Any intervention identified was categorised into these three domains (population health and public policy, health service and integrative) and included in the review. The plan was to use the Template for Intervention Description and Replication—Population Health and Policy to extract relevant contextual information, however, the level of implementation information contained within papers was insufficient to use this consistently. The planned subgroup analysis of the socioeconomic patterning of intervention equity impacts by age, gender and ethnicity, as specified in the protocol,^1^ was not possible because not enough studies provided this information and for those that did, reporting of subgroup analysis was not consistent.

A change to the protocol was agreed to aid study synthesis and interpreting the results. The planned systematic review outlined in the protocol became two separate reviews with a unifying methodological approach. Here, studies where the differential effectiveness of the intervention across socioeconomic groups has been examined are reported. Studies of targeted interventions (implemented only for people experiencing socioeconomic disadvantage) were excluded from this analysis but are reported in an allied review^29^ as these share common characteristics and are distinct from the interventions included in this review.

### Patient and public involvement

This systematic review is part of the UNFAIR research programme.^23^ Four members of the public sat on the UNFAIR advisory panel and contributed to, for example, discussions around the development of the theoretical framework for this systematic review (framework published in the protocol).^1^ As part of UNFAIR, diverse members of the public and local communities contributed to developing an animation, which provides powerful insights into what health inequalities mean to people with examples of lived experience,^30^ and this activity was co-led by one of the UNFAIR public contributors. This animation produced through public involvement corroborates the moral argument for the importance of this systematic review to understand what works to reduce inequalities in hospitalisations.

## Results

After removing duplicates, 25 618 titles and abstracts were screened, followed by 563 full texts (figure 1—PRISMA flow diagram). After applying inclusion and exclusion criteria, 36 studies were selected for inclusion (see online supplemental file 3: reasons for exclusion). The 36 studies were published between 2001 and 2023 with 15 (42%) published in the last 5 years since 2019 indicating how interest in health equity has grown in recent years. Half of all included studies came from one country: 18 (50%) were based in the USA, 8 studies (22%) in the UK, 4 studies (11%) in Canada, 2 studies (6%) in Spain, and one study each from New Zealand, Norway, Finland and Sweden (online supplemental table 1).

Ten studies reported the socioeconomic equity impact of an intervention on the outcome hospital readmission, 25 studies reported on the outcome hospitalisation and one study reported on both hospitalisations and readmissions. 11 studies considered all cause admissions, 24 were condition specific and 1 study considered both. Condition-specific admissions included asthma, cardiovascular disease, communicable disease, maternal admissions and composite measures including admissions for ambulatory care sensitive conditions, admissions attributable to alcohol or to opioid misuse or to road traffic accidents (online supplemental table 1).

Half of the included studies were health service interventions. These included primary prevention healthcare interventions (four vaccine studies), secondary/tertiary prevention healthcare interventions (eg, pharmacist medication intervention, patient self-care training) and studies reporting the equity impacts of changes to healthcare regulation, organisation and financing policy implemented at either a local, regional state or national level (12 studies). Seven studies reported integrative interventions; two holistic interventions in Spain for heart failure^31^ and multimorbid patients,^32^ an Integrated Care Team intervention in England with a clinical care-coordinator role working alongside staff from the NHS, local council and voluntary sector,^33^ a US study involving integrated peer coaching for parents of children with asthma,^34^ a US intervention to reorganise primary care to be more integrated and holistic,^35^ a US hospital community partnership initiative^36^ and a Drug Treatment court in Canada that addressed interdependent medical, social and legal issues faced by individuals who commit minor offences as a result of their substance use.^37^ There were 11 population health intervention studies. These included the impact of legislation to implement or change the minimum unit alcohol sale price,^38^,^40^ the age of state retirement in a country,^41^ adopting smoke-free public places,^42 43^ smoke-free vehicle,^44^ car booster seat legislation,^45^ water fluoridation,^46^ enforcing air quality management areas^47^ and implementing a smoke-free home mass media campaign.^43^

As summarised in online supplemental table 1, socioeconomic status of study participants was variously:

- Categorised; including education or health literacy, income, insurance status/Medicaid receipt, a composite metric or several different markers of socioeconomic position for one study.
- Measured; individual-level (eg, a person’s income, employment status or education level), individual-hospital level (eg, safety net status) or area-based measure derived from individual’s place of residence (eg, a deprivation index) or a combination of both individual-level and area-based measures.
- Reported; socioeconomic dimension of outcome reported a categorical (eg, insurance status)^48^ or ordered categorical classification (eg, quintiles or deciles of deprivation).^40 42^

There was no indication that how socioeconomic status was categorised, measured or reported across studies had a systematic impact on the results (eg, it was not the case that all studies measuring socioeconomic status on an individual level reported reducing inequalities, whereas those relying on area-based measure of deprivation reported increases); however, this variation hampered efforts to pool effects and compare across studies.

### Study quality

Included studies employed a range of study designs; 3 RCTs, 6 quasi-experimental, 2 cross-sectional and 3 cross-sectional time series, 12 interrupted time-series, 10 cohort (online supplemental table 1 and table 2). Studies were of variable quality (table 1) with the majority rated as high (N=19) or moderate (N=13) quality. There were no clear trends in study quality rating with respect to, for example, how socioeconomic status was measured, how recently the article was published or domain of intervention activity although there were no weakly rated integrative studies (table 1). Half (seven of 13) of the studies reporting a decrease in inequalities relating to the intervention were rated as high quality compared with only a third (two out of six) of studies reporting an increase in inequalities (table 3).

**Table 1:** Study quality assessment by domain of intervention

Domain of intervention	EPHPP tool global quality rating		
	Strong	Moderate	Weak
Health service	Bell *et al*^70^ Chou *et al*^59^ Sankaran *et al*^57^ DeWalt *et al*^58^ Hungerford *et al*^62^ Murty *et al*^71^ Petousis *et al*^72^	Angraal *et al*^48^ Colla *et al*^52^ Connell *et al*^53^ Wharam *et al*^73^ Salerno *et al*^56^ Gosselin A *et al*^60^ Gosselin B *et al*^61^ Lu *et al*^54^ Madden *et al*^55^	Blanchard *et al*^50^ Pimentel *et al*^74^
Population health and public policy	Millett *et al*^42^ Turner *et al*^43^ Zhao and Stockwell^38^ Grotting and Lillebø^41^ MacKay *et al*^44^ Almquist *et al*^75^ Wyper *et al*^40^	Rose *et al*^47^ Herrtua *et al*^39^	Elmer *et al*^46^ Pressley *et al*^45^
Integrative	Cheon *et al*^36^ Soto-Gordoa *et al*^32^ Meyers *et al*^35^ Capdevila *et al*^31^ Prioddi *et al*^33^	Garbutt *et al*^34^ Rezansoff *et al*^37^	

### Impact of interventions on socioeconomic inequalities in hospitalisations

Six studies reported increasing inequalities (table 2). Interventions that exacerbated inequalities were found across all three domains of activity (table 3). In seven studies, the intervention appeared to maintain prevailing relative inequalities across socioeconomic groups in hospitalisations and readmissions (eg, the rate ratios for the impact of the intervention on hospitalisations were similar in all quintiles of area deprivation).^42^ In 10 studies, the reported impact of the intervention on socioeconomic inequalities in hospitalisations or readmissions was either mixed or inconclusive. All of the studies examining intervention impact on socioeconomic inequalities in readmissions were health service-based interventions^48^,^57^ apart from one integrative intervention study,^36^ and all were based in the USA. These studies reported mixed or inconclusive evidence of impact of interventions on inequalities in readmissions, with the exception of two studies on financial incentivisation and penalty as part of US healthcare reform,^52 56^ which reported positive impacts for reducing inequalities (table 2).

**Table 2:** Direction of effect plot^27^

Study	Study design	Socioeconomic inequalities in hospitalisations	Socioeconomic inequalities in readmissions
DeWalt *et al*^58^	RCT	▼	
Grotting and Lillebø^41^	QEX	▼	
Hungerford *et al*^62^	ITS	▼	
Soto-Gordoa *et al*^32^	Cohort	▼	
Murty *et al*^71^	XS	▼	
Wyper *et al*^40^	ITS	▼	
Zhao and Stockwell^38^	XS(TS)	▼	
Garbutt *et al*^34^	RCT	▼	
Colla *et al*^52^	QEX		▼
Rose *et al*^47^	QEX	▼	
Salerno *et al*^56^	ITS		▼
Elmer *et al*^46^	XS	▼	
Pimentel *et al*^74^	XS(TS)	▼	
Bell *et al*^70^	RCT		◄►
Cheon *et al*^36^	Cohort		◄►
Millett *et al*^42^	ITS	◄►	
Sankaran *et al*^57^	QEX		◄►
Angraal *et al*^48^	XS(TS)		◄►
Madden *et al*^55^	ITS		◄►
Rezansoff *et al*^37^	Cohort	◄►	
Capdevila *et al*^31^	Cohort	▲	
MacKay *et al*^44^	ITS	▲	
Gosselin A *et al*^60^	Cohort	▲	
Gosselin B *et al*^61^	Cohort	▲	
Herrtua *et al*^39^	XS(TS)	▲	
Pressley *et al*^45^	Cohort	▲	
Almquist and Miething^75^	ITS	?	
Chou *et al*^59^	ITS	?	?
Meyers *et al*^35^	QEX	?	
Petousis *et al*^72^	Cohort	?	
Piroddi *et al*^33^	QEX	?	
Turner *et al*^43^	ITS	**?**	
Connell *et al*^53^	ITS		**?**
Lu *et al*^54^	ITS		**?**
Wharam *et al*^73^	ITS	**?**	
Blanchard *et al*^50^	Cohort		**?**

Study design: RCT, QEX, XS, XS(TS), ITS, cohort.

Effect direction: upward arrow ▲=increases inequalities; ▼=reduces inequalities; sideways arrow ◄►=maintains inequalities; ?=unclear or mixed effects.

Study quality: denoted by row colour: green=low risk of bias; amber=some concerns; red=high risk of bias.

ITS, interrupted time-series;; QEX, quasi-experimental; RCT, randomised controlled trial; XS, cross-sectional; XS(TS), cross-section (time series); .

**Table 3:** Impact* of interventions on socioeconomic inequalities in hospitalisations or readmissions according to domain of intervention and study quality

Domain of intervention	Impact of intervention on socioeconomic inequalities in hospitalisations or readmissions			
	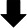 Decreased	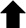 Increased	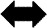 Maintained	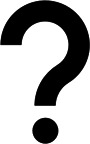 Mixed/inconclusive
Health service	6 studies: Colla *et al*^52^Salerno *et al*^56^DeWalt *et al*^58^ Hungerford *et al*^62^ Murty *et al*^71^ Pimentel *et al*^74^	2 studies: Gosselin A *et al*^60^Gosselin B *et al*^61^	4 studies: Angraal *et al*^48^ Bell *et al*^70^ Sankaran *et al*^57^ Madden *et al*^55^	6 studies: Blanchard *et al*^50^ Chou *et al*^59^ Connell *et al*^53^ Wharam *et al*^73^ Lu *et al*^54^ Petousis *et al*^72^
Population health and public policy	5 studies: Zhao and Stockwell^38^ Rose *et al*^47^ Elmer *et al*^46^ Grotting and Lillebø^41^ Wyper *et al*^40^	3 study: Herrtua *et al*^39^ MacKay *et al*^44^ Pressley *et al*^45^	1 study: Millett *et al*^42^	2 studies: Turner *et al*^43^ Almquist and Miething^75^
Integrative	2 studies: Soto-Gordoa *et al*^32^ Garbutt *et al*^34^	1 study: Capdevila *et al*^31^	2 studies: Cheon *et al*^36^ Rezansoff *et al*^37^	2 studies: Meyers *et al*^35^ Piroddi *et al*^33^

Effective Public Health Practice Project (EPHPP) global quality rating. Red: weak (high risk of bias), orange: moderate (some concerns), green: strong (low risk of bias).

* Modified harvest plot with vote counting, based on direction of effect.

Overall, we interpreted 13 studies as showing an intervention reduced socioeconomic inequalities in hospitalisations or readmissions. Table 2 presents the impact of interventions on inequalities in hospitalisations or readmissions using a direction of effect plot and vote counting against four categories (increased, decreased, maintained and mixed/inconclusive impact on socioeconomic inequalities). Table 3 summarises the impact of interventions on socioeconomic inequalities in hospitalisations or readmissions in a modified harvest plot according to domain of intervention and study quality. Online supplemental table 2 outlines study outcomes for all 36 studies. Effective interventions were found in all domains of action (health service, integrative and population health and public policy) and were characterised as either:

1. Interventions tailored to the differing needs and contexts of individuals across socioeconomic groups, which are supportive in their design and delivery^32 34 58^ (table 3). For example, a multisession self-help intervention delivered to patients with heart failure was more effective for those with low literacy at reducing hospitalisations^58^ compared with a less supportive version of the intervention, which only provided a single session.
2. Being legislative in nature addressing the rules and resources in society, applied and enforced at a population or health system level,^41 46 47 52 56^ which requires limited voluntary behaviour change on the part of an individual person or patient.

An exemplar of the second characteristic is the 2006 smoke-free legislation in Scotland (an intervention restricting opportunities and places to smoke mandated by law, applied to the whole population and importantly rigorously monitored and enforced). This intervention was found to be associated with reduced inequalities in asthma hospitalisations. In contrast, the 2014 ‘take it right out’ mass-media campaign (design to persuade individuals to change their behaviour and not mandated to the entire population) was not; asthma admissions did not change in any of the three deprivation categories as a result of the mass-media intervention.^43^ Smoke-free vehicle legislation was regulatory in nature and applied to the whole population but unlike the ban on smoking in public places lacked rigorous monitoring and enforcement (no judicial cases). This policy instead relied on voluntary adherence (individuals having an awareness of the law and choosing not to smoke in their car). The smoke-free vehicle legislation increased relative inequalities^44^ as did child booster seat legislation, which again required individual resource and proactive behaviour change (responsibility of an individual to source a booster seat and put this in a car for a child to use). For legislative interventions to work to reduce prevailing inequalities, comprehensive monitoring and enforcement of the law are an important implementation component.

Government intervention to change minimum unit pricing for the sale of alcohol, which is applied universally and enforced for every purchase, impacts on socioeconomic inequalities in hospitalisations. Introducing minimum unit pricing for alcohol in Scotland^40^ and increasing minimum unit pricing for alcohol in Canada^38^ resulted in decreasing socioeconomic inequalities in hospitalisations. In contrast, an opposing policy to reduce rather than increase minimum unit pricing for alcohol in Finland^39^ had the reverse effect of increased inequalities in hospitalisations.

Two integrative interventions (a holistic patient-centred programme^32^ and a telephone peer coaching intervention)^34^ led to reductions in socioeconomic inequalities. These studies were conducted in countries with different healthcare systems, the USA and Spain, suggesting that the principles of designing supportive interventions tailored to the needs of individuals from different socioeconomic groups are important irrespective of the funding model and structuring of healthcare provision in a country. However, not all integrative interventions had an impact on reducing hospitalisation inequalities.^33^ For example, while safety-net hospitals in the USA serving the most socioeconomically disadvantaged communities were more engaged in hospital-community partnerships, with local public health, local government, social services, charitable and voluntary sector and insurance companies, relative to their non-safety-net peers, these partnerships were not significantly related to reductions in readmission rates, thereby maintaining existing inequalities.^36^ All of the successful interventions identified for reducing socioeconomic inequalities in hospitalisations and readmissions within the health service domain were US-based studies, indicating that in a context where universal publicly funded healthcare is not in place there is scope for health service-based interventions to be effective.

Disentangling whether an intervention has a positive or negative impact on health equity can be complex. For example, one study reported that the impact on inequalities differed according to whether the outcome of initial hospitalisation or readmission was considered; while employer-mandated switches to high deductible health plan enrolment led to a significant decrease in index hospitalisations for non-specific chest pain inpatients from low-income neighbourhoods, it also resulted in an increase in 30-day readmissions for acute myocardial infarction in these neighbourhoods.^59^ In another example, no consistent trend in impact across socioeconomic strata was identified; an integrated care multidisciplinary team intervention led to an increase in emergency admissions a year after the intervention, both in the least deprived quintile and also in the fourth most deprived quintile with non-significant impacts across other quintiles.^33^

Vaccine studies included in the review reported differing equity impacts but, if implemented in ways consistent with principles of proportional universalism, provide an important tool to address health inequalities. Two studies (Gosselin *et al*^60^ ^61^), both reporting on the impact of a rotavirus vaccine implemented in Canada, found that the intervention increased relative inequalities across socioeconomic groups in hospitalisations. All socioeconomic categories showed a reduced hospitalisation rate in the postprogramme period, but the lowest relative reductions were observed in children living in neighbourhoods with higher rates of unemployment, low-income families and single mothers.^61^ The intervention did, however, decrease absolute inequalities on account of the higher prevalence of communicable disease in lower socioeconomic groups.^61^ Hungerford *et al*^62^ report that in England, the rotavirus vaccine reduced prevailing inequalities in acute gastroenteritis hospitalisations, having the greatest impact among the most deprived populations, despite lower vaccine uptake in these populations. Hungerford concludes that prioritising vaccine uptake in socioeconomically deprived communities should, therefore, give the greatest health benefit in terms of population disease burden.^62^

## Discussion

### Main findings

A commonality across the international evidence base was the failure of studies to evaluate equity impacts; despite the comprehensive scope of this review, only 36 studies met the inclusion criteria and reported the differential impact of interventions on hospitalisations or readmissions across socioeconomic groups. Many studies routinely report sociodemographic baseline characteristics of participants but do not examine whether an intervention had differential effectiveness according to characteristics such as socioeconomic status, making it impossible to ascertain if the intervention worked, and to what extent, for some groups compared with others within society at preventing hospitalisations or readmissions.

Socioeconomic disadvantage was variously categorised, measured and reported in studies making comparison of equity impacts across studies challenging. 13 studies reported decreases in inequalities in hospitalisations between socioeconomic groups, 7 maintained inequalities, 6 increased them, and 10 had mixed or inconclusive impacts. These mixed findings both within and across studies underscore the importance of understanding intervention components and implementation context in order to establish the equity impact of interventions.

There were examples of effective health service and integrative interventions for reducing socioeconomic inequalities in healthcare outcomes (hospitalisations and readmissions), but importantly many effective interventions were non-healthcare (eg, smoke-free legislation, raising minimum unit pricing for alcohol sales).

Interventions that reduced socioeconomic inequalities were those implemented and enforced across entire populations and systems or those which were supportive and tailored to the differing needs of individuals across socioeconomic groups in their design and delivery.

### Comparison to other studies

The lack and variability in reporting of equity impacts across intervention effectiveness literature for healthcare outcomes uncovered in our review adds weight to concerns over poor consistency and priority given to this area.^63^ Our finding that health equity promoting interventions are those which are supportive and tailored to the needs of individuals is consistent with a previous systematic review of interventions to prevent readmissions; although that review did not examine equity impacts and focused on healthcare interventions only, it did report that more effective interventions in preventing readmissions are complex in nature and support patient capacity for self-care.^14^ Our finding is also consistent with a previous systematic review of effectiveness of health service interventions on reducing inequalities, which reported that characteristics of successful interventions included a systematic, intensive and multidisciplinary approach, enhanced access and service utilisation, addressing the needs of the target populations with community involvement.^16 17 64^

Individuals experiencing socioeconomically marginalised circumstances tend to be less able to benefit from interventions, which require a high degree of self-agency to enact.^9 15^ Interventions directed to individuals, such as self-care interventions, therefore risk-introducing intervention-generated inequalities (IGIs).^15^ Interventions may also increase rather than decrease admissions in the short term, if this is the result of improved identification of unmet needs in different groups within the population through, for example, multidisciplinary teams.^33^ Our finding that enforced legislative interventions (for example, introducing^40^ or increasing minimum unit pricing of alcohol sales^38^ and water fluoridation)^46^ were effective at reducing inequalities in hospitalisations is consistent with the IGI literature and existing evidence base around what works to reduce health and care inequalities.^9 15^ The IGI literature also supports our finding that the nature and degree of support provided to individuals are critical to the extent to which any self-care intervention will reduce inequalities in healthcare outcome or at the very least mitigate against exacerbating prevailing inequity.^17^ Indeed, a previous review of interventions to reduce unplanned hospitalisations, which again did not examine equity impacts specifically, reported that there was evidence that self-management, exercise and telemedicine-type interventions appeared to work but *only* in selected patient populations.^13^

Finally, our finding that whole systems and cross-sectoral approaches including population-level public policy intervention, not just health service interventions alone, are required to reduce inequalities in hospital outcomes (hospitalisations and readmissions), is supported by established theory and evidence outlining the necessity to intervene to improve the social determinants of health^89 65^,^67^ in order to improve health equity.

### Strengths and limitations

Our equity review was comprehensive in scope; including any intervention across all domains of activity, examining the outcome hospitalisation or readmission for all or any cause, in any population within an OECD country over the past 24 years. Including papers since the millennium enabled a comprehensive examination of the literature, while ensuring the included studies are of relevance in today’s context, and that both the prepandemic and postpandemic periods were included. The rigorous international gold-standard PRISMA-E methodology was employed to undertake and report the equity-focused systematic review and a recently developed and validated comprehensive search strategy^24^ for identifying studies focused on equity issues was used. The review is, therefore, at the forefront of developments in equity-based systematic reviewing practice. Even though the search strategy was comprehensive, it may have missed studies, which did not make any reference to equity-related terms in either the title or abstract but did report on this in the main text.

Study quality was variable, and this, coupled with the limited number of primary papers identified which addressed our research questions, influences the degree of confidence in the conclusions drawn from this review. Nevertheless, in terms of understanding what interventions work to reduce inequalities, half of the included review papers showing reductions in inequalities were rated highly. We have used the EPHPP screening tool^26^ for quality assessment and screening, because our review includes a range of quantitative study types, including quasi-experimental methods papers, and this tool enables consistent quality assessment across a range of quantitative study designs. Our focus on hospitalisations and readmissions as the outcome of interest implies that reducing hospitalisations is always both possible and desirable. However, some hospitalisations are of course entirely justified and will be both necessary and appropriate to meet patients’ needs. It would have strengthened the equity review to consider a wider range of healthcare outcomes, but this was beyond the scope of the resources for this review. Inequalities are multiple, complex and intersectional,^8 68^ and it would have strengthened the paper to consider other equity dimensions such as ethnicity. A subgroup analysis of the socioeconomic patterning of intervention equity impacts by age, gender and ethnicity was planned, but this was not possible because not enough studies provided this information and for those that did, the reporting of subgroup analysis was not consistent.

In recognition of the wide discrepancies in healthcare system resources and arrangements, we only included interventions in OECD countries. Nevertheless, there are still considerable differences in health system arrangements across OECD countries. For example, more than half the studies included in the review were conducted in the USA, which has a very different healthcare system to many European countries. A potential limitation, therefore, is the generalisability of findings from this review to different contexts, including non-OECD country settings.

### Implications

The level and consistency of monitoring of equity impacts of interventions internationally must be improved given this review shows that what works to reduce hospitalisations and readmissions for some members of society may not work, or work less well, for others. Increased use of clear and consistent definitions and recording of socioeconomic status (at both an area and individual level) and reporting of outcome data according to these classifications would aid comparison and synthesis. The recent move by the National Institute for Health and Care Research in the UK to mandate consideration and monitoring of equity in all funded research studies is a promising step in this direction.^69^

Interventions implemented and enforced across entire populations and systems and those which are supportive and tailored to differing needs of individuals across socioeconomic groups in their design and delivery are likely to address the twin policy objectives of reducing hospital admissions and addressing health inequalities. Therefore, investment in population health and integrative activity addressing the social determinants of health, alongside health service interventions, is required to improve equity in hospital outcomes. Policymakers must redouble efforts to work across agencies and departments to meaningfully address health and care inequity.

## Supplementary material

10.1136/bmjph-2025-002595 online supplemental file 1

10.1136/bmjph-2025-002595 online supplemental file 2

10.1136/bmjph-2025-002595 online supplemental file 3

10.1136/bmjph-2025-002595 online supplemental file 4

10.1136/bmjph-2025-002595 online supplemental file 5

## Data Availability

Data sharing not applicable as no datasets generated and/or analysed for this study.
