# Supplementary material for: What works to reduce socioeconomic inequalities in hospitalisations and readmissions? Systematic review of the equity impacts of population-level, health service and integrative interventions
Source: BMJ Public Health. 2025 Sep 23;3(2):e002595. doi: 10.1136/bmjph-2025-002595 (PMC12458884; doi:10.1136/bmjph-2025-002595)
Supplement: online supplemental file 2 [file bmjph-3-2-s002.docx]

**Supplementary files**

- File 1: PRISMA ABSTRACT checklist, PRISMA-E checklist and SWIM reporting guideline
- File 2: search strategies (MEDLINE, Embase, CINAHL, Web of Knowledge)
- File 3: Reasons for exclusion after full-text review
- File 4: Table 1 – Study characteristics
- File 5: Table 2 – Study outcomes (impact of interventions on socioeconomic inequalities in hospitalisations or readmissions)

**Supplementary file 2: Search strategies (MEDLINE, Embase, CINAHL, Web of Knowledge)**

**MEDLINE search strategy**

| **#** | **Searches** |
| --- | --- |
| 1 | Hospitalization/ |
| 2 | Patient Readmission/ |
| 3 | Patient Admission/ |
| 4 | ((emergency or unplanned or unanticipated or unexpected or avoid*) adj3 (admission* or readmission* or hospitali#ation*)).ti,ab,kw. |
| 5 | (overnight stay adj3 admission*).ti,ab,kw. |
| 6 | (primary care adj3 admission*).ti,ab,kw. |
| 7 | (ambulatory care adj3 admission*).ti,ab,kw. |
| 8 | or/1-7 |
| 9 | ((program* or policy or policies or strateg* or scheme* or intervention* or project* or initiative*) adj5 (evaluat* or effect* or measur* or assess* or experiment* or impact*)).ti,ab,kw. |
| 10 | (comparative study or controlled clinical trial or evaluation studies or meta analysis or pragmatic clinical trial or randomized controlled trial or "systematic review").pt. |
| 11 | exp Clinical Trial/ or exp Randomized Controlled Trial/ or exp Randomization/ or Random Allocation/ or Double-Blind Method/ or Single-Blind Method/ or exp Cross-Over Studies/ or Program Evaluation/ |
| 12 | (RCT or randomi* or nonrandomi* or non randomi* or quasiexperiment* or quasi experiment* or quasirandomi* or quasi randomi* or pseudoexperiment* or pseudo experiment* or pseudorandomi* or pseudo randomi* or natural experiment* or pretest or pre test or posttest or post test or time series or repeat* measure* or systematic review*).ti,ab,kw. |
| 13 | (before adj1 after adj1 (stud* or trial* or design*)).ti,ab. |
| 14 | or/9-13 |
| 15 | 8 and 14 |
| 16 | (Algeria* or Egypt* or Liby* or Morocc* or Tunisia* or Western Sahara* or Angola* or Benin or Botswana* or Burkina Faso or Burundi or Cameroon or Cape Verde or Central African Republic or Chad or Comoros or Congo or Djibouti or Eritrea or Ethiopia* or Gabon or Gambia* or Ghana or Guinea or Keny* or Lesotho or Liberia or Madagasca* or Malawi or Mali or Mauritania or Mauritius or Mayotte or Mozambiq* or Namibia* or Niger or Nigeria* or Reunion or Rwand* or Saint Helena or Senegal or Seychelles or Sierra Leone or Somalia or South Africa* or Sudan or Swaziland or Tanzania or Togo or Ugand* or Zambia* or Zimbabw* or China or Chinese or Hong Kong or Macao or Mongolia* or Taiwan* or Belarus or Moldov* or Russia* or Ukraine or Afghanistan or Armenia* or Azerbaijan or Bahrain or Cyprus or Cypriot or Georgia* or Iran* or Iraq* or Israel* or Jordan* or Kazakhstan or Kuwait or Kyrgyzstan or Leban* or Oman or Pakistan* or Palestin* or Qatar or Saudi Arabia or Syria* or Tajikistan or Turkmenistan or United Arab Emirates or Uzbekistan or Yemen or Bangladesh* or Bhutan or British Indian Ocean Territory or Brunei Darussalam or Cambodia* or India* or Indonesia* or Lao or People's Democratic Republic or Malaysia* or Maldives or Myanmar or Nepal or Philippin* or Singapore or Sri Lanka or Thai* or Timor Leste or Vietnam or Albania* or Andorra or Bosnia* or Herzegovina* or Bulgaria* or Croatia* or Estonia or Faroe Islands or Greenland or Liechtenstein or Lithuani* or Macedonia or Malta or maltese or Romania or Serbia* or Montenegro or Slovenia or Svalbard or Argentina* or Belize or Bolivia* or Brazil* or chile or Chilean or Colombia* or Costa Rica* or Cuba or Ecuador or El Salvador or French Guiana or Guatemala* or Guyana or Haiti or Honduras or Jamaica* or Nicaragua* or Panama or Paraguay or Peru or Puerto Rico or Suriname or Uruguay or Venezuela or developing countr* or south America*).ti,sh. |
| 17 | 15 not 16 |
| 18 | limit 17 to (english language and humans and yr="1999 -Current") |
| 19 | Residence Characteristics/ |
| 20 | Environment design/ |
| 21 | exp Marital status/ |
| 22 | neighbo?rhood*.mp. |
| 23 | residential environment*.mp. |
| 24 | rural*.mp. |
| 25 | inner?city.mp. |
| 26 | housing instability.mp. |
| 27 | housing insecurity.mp. |
| 28 | housing strain.mp. |
| 29 | housing security.mp. |
| 30 | mortgage problems.mp. |
| 31 | foreclosure.mp. |
| 32 | eviction*.mp. |
| 33 | housing loss.mp. |
| 34 | home repossession*.mp. |
| 35 | home ownership.mp. |
| 36 | (repossess* adj3 hous*).mp. |
| 37 | (repossess* adj3 propert*).mp. |
| 38 | mortgage delinquency.mp. |
| 39 | mortgage arrears.mp. |
| 40 | mortgage debt*.mp. |
| 41 | overcrowding.mp. |
| 42 | (living adj1 (outside or inside or near* or adjacent)).mp. |
| 43 | (household adj2 size).mp. |
| 44 | (marital status or marriage status).mp. |
| 45 | (widow* or cohabit* or divorce* or single parent* or live* alone).mp. |
| 46 | or/19-45 |
| 47 | Occupations/ |
| 48 | Unemployment/ |
| 49 | occupations.mp. |
| 50 | unemployment.mp. |
| 51 | or/47-50 |
| 52 | exp Educational status/ |
| 53 | Education/ |
| 54 | Schooling.mp. |
| 55 | educational status.mp. |
| 56 | (education* adj2 level?).mp. |
| 57 | ((higher or better or worse or less) adj educated).mp. |
| 58 | ((higher or better or worse or less) adj level? of education).mp. |
| 59 | or/52-58 |
| 60 | Social determinants of Health/ |
| 61 | Psychosocial Deprivation/ |
| 62 | Sociological Factors/ |
| 63 | Working Poor/ |
| 64 | Hierarchy, Social/ |
| 65 | disparit*.mp. |
| 66 | inequalit*.mp. |
| 67 | inequit*.mp. |
| 68 | equity.mp. |
| 69 | deprivation.mp. |
| 70 | gini.mp. |
| 71 | concentration index.mp. |
| 72 | Socioeconomic Factors/ |
| 73 | Social Welfare/ |
| 74 | exp Social Class/ |
| 75 | exp Poverty/ |
| 76 | Income/ |
| 77 | Social class*.mp. |
| 78 | social determinants.mp. |
| 79 | social status.mp. |
| 80 | social position.mp. |
| 81 | social background.mp. |
| 82 | social circumstance*.mp. |
| 83 | socio-economic.mp. |
| 84 | socioeconomic.mp. |
| 85 | sociodemographic.mp. |
| 86 | socio-demographic.mp. |
| 87 | SES.mp. |
| 88 | disadvantaged.mp. |
| 89 | impoverished.mp. |
| 90 | poverty.mp. |
| 91 | economic level.mp. |
| 92 | assets index.mp. |
| 93 | income*.mp. |
| 94 | or/60-93 |
| 95 | Social Stigma/ |
| 96 | social capital/ |
| 97 | Social Control, Informal/ |
| 98 | exp Social Support/ |
| 99 | exp Social Environment/ |
| 100 | Trust/ |
| 101 | Social conditions/ |
| 102 | Social isolation/ |
| 103 | Social marginalization/ |
| 104 | Anomie/ |
| 105 | social participation/ |
| 106 | social exclusion.mp. |
| 107 | (social adj (capital or cohes* or organis* or organiz*)).mp. |
| 108 | (community adj3 (cohes* or participa*)).mp. |
| 109 | ((neighbourhood or neighborhood) adj cohes*).mp. |
| 110 | social relationships.mp. |
| 111 | social network*.mp. |
| 112 | collective efficacy.mp. |
| 113 | civil society.mp. |
| 114 | informal social control.mp. |
| 115 | neighbo*rhood disorder.mp. |
| 116 | social disorgani?ation.mp. |
| 117 | anomie.mp. |
| 118 | social support.mp. |
| 119 | social participation.mp. |
| 120 | trust.mp. |
| 121 | emotional support.mp. |
| 122 | psychosocial support.mp. |
| 123 | community capital.mp. |
| 124 | neighbo*rhood cohesion.mp. |
| 125 | social influence.mp. |
| 126 | (soci*context* or soci*-context*).mp. |
| 127 | or/95-126 |
| 128 | Health Status Disparities/ |
| 129 | Health Services Accessibility/ |
| 130 | Health Equity/ |
| 131 | health*care disparit*.mp. |
| 132 | health care disparit*.mp. |
| 133 | health status disparit*.mp. |
| 134 | health disparit*.mp. |
| 135 | health inequalit*.mp. |
| 136 | health inequit*.mp. |
| 137 | medically underserved.mp. |
| 138 | or/128-137 |
| 139 | potential determinants.mp. |
| 140 | significant correlates of.mp. |
| 141 | (independent correlates or independent association*).mp. |
| 142 | variables associated with.mp. |
| 143 | determinants of.mp. |
| 144 | factors associated with.mp. |
| 145 | identif* determinants.mp. |
| 146 | (more likely or less likely or just as likely).mp. |
| 147 | risk factors for.mp. |
| 148 | (significantly related to or significant predictor).mp. |
| 149 | (also adj2 associated with).mp. |
| 150 | (at increased risk or at decreased risk).mp. |
| 151 | association* between.mp. |
| 152 | (positively associated or negatively associated).mp. |
| 153 | differed by.mp. |
| 154 | (were high* amongst or were low* amongst).mp. |
| 155 | (inverse relationship with or inversely associated with or inversely related to).mp. |
| 156 | reverse association.mp. |
| 157 | differentially affects.mp. |
| 158 | evidence of a link between.mp. |
| 159 | (significantly adj3 likelihood of).mp. |
| 160 | protective factors for.mp. |
| 161 | (differ* adj2 according to).mp. |
| 162 | (inverse adj2 gradient).mp. |
| 163 | (positive adj2 gradient).mp. |
| 164 | (negative adj2 gradient).mp. |
| 165 | (trends were adj3 across).mp. |
| 166 | (related to adj3 variable*).mp. |
| 167 | (differences were adj3 explained by).mp. |
| 168 | (significant among or no# significant among).mp. |
| 169 | or/139-168 |
| 170 | 46 or 51 or 59 or 94 or 127 or 138 or 169 |
| 171 | 18 and 170 |

**EMBASE search strategy**

| 1 | demography/ |
| --- | --- |
| 2 | environmental planning/ |
| 3 | marriage/ |
| 4 | divorce/ |
| 5 | cohabitation/ |
| 6 | widow/ |
| 7 | exp "single (marital status)"/ |
| 8 | neighbo?rhood*.mp. |
| 9 | residential environment*.mp. |
| 10 | rural*.mp. |
| 11 | inner?city.mp. |
| 12 | housing instability.mp. |
| 13 | housing insecurity.mp. |
| 14 | housing strain.mp. |
| 15 | housing security.mp. |
| 16 | mortgage problems.mp. |
| 17 | foreclosure.mp. |
| 18 | eviction*.mp. |
| 19 | housing loss.mp. |
| 20 | home repossession*.mp. |
| 21 | home ownership.mp. |
| 22 | (repossess* adj3 hous*).mp. |
| 23 | (repossess* adj3 propert*).mp. |
| 24 | mortgage delinquency.mp. |
| 25 | mortgage arrears.mp. |
| 26 | mortgage debt*.mp. |
| 27 | overcrowding.mp. |
| 28 | (living adj1 (outside or inside or near* or adjacent)).mp. |
| 29 | (household adj2 size).mp. |
| 30 | (marital status or marriage status).mp. |
| 31 | (widow* or cohabit* or divorce* or single parent* or live* alone).mp. |
| 32 | or/1-31 |
| *33* | *exp cultural deprivation/* |
| *34* | *cultural factor/* |
| *35* | *cultural anthropology/* |
| *36* | *cultural diversity/* |
| *37* | *exp migrant/* |
| *38* | *minority group/* |
| *39* | *minority health/* |
| *40* | *prejudice/* |
| *41* | *exp social discrimination/* |
| *42* | *exp race relation/* |
| *43* | *exp ethnic group/* |
| *44* | *exp ancestry group/* |
| *45* | *exp refugee/* |
| *46* | *minorit*.mp.* |
| *47* | *migration background.mp.* |
| *48* | *racial.mp.* |
| *49* | *racism.mp.* |
| *50* | *ethnology.mp.* |
| *51* | *race.mp.* |
| *52* | *ethnic*.mp.* |
| *53* | *non?English.mp.* |
| *54* | *language other than.mp.* |
| *55* | *latino*.mp.* |
| *56* | *latina*.mp.* |
| *57* | *hispanic*.mp.* |
| *58* | *whites.mp.* |
| *59* | *caucasian*.mp.* |
| *60* | *non?white.mp.* |
| *61* | *Torres Strait Islander.mp.* |
| *62* | *aboriginal.mp.* |
| *63* | *native american.mp.* |
| *64* | *inuit.mp.* |
| *65* | *eskimo.mp.* |
| *66* | *first nation*.mp.* |
| *67* | *indigenous.mp.* |
| *68* | *english as a second language.mp.* |
| *69* | *foreign language.mp.* |
| *70* | *or/33-69* |
| 71 | exp employment status/ |
| 72 | job characteristics/ |
| 73 | occupations.mp. |
| 74 | unemployment.mp. |
| 75 | or/71-74 |
| *76* | *exp gender identity/* |
| *77* | *women's health/* |
| *78* | *sex difference/* |
| *79* | *(sex disparit* or sex difference?).mp.* |
| *80* | *gender identity.mp.* |
| *81* | *sex role.mp.* |
| *82* | *wom#n* role?.mp.* |
| *83* | *m#n* role?.mp.* |
| *84* | *gender* role?.mp.* |
| *85* | *servicewomen.mp.* |
| *86* | *or/76-85* |
| 87 | exp educational status/ |
| 88 | schooling.mp. |
| 89 | educational status.mp. |
| 90 | (education* adj2 level?).mp. |
| 91 | ((higher or better or worse or less) adj educated).mp. |
| 92 | ((higher or better or worse or less) adj level? of education).mp. |
| 93 | or/87-92 |
| *94* | *religion/* |
| *95* | *religi*.mp.* |
| *96* | *or/94-95* |
| 97 | "social determinants of health"/ |
| 98 | social aspect/ |
| 99 | working poor/ |
| 100 | exp social hierarchy/ |
| 101 | socioeconomics/ |
| 102 | disparit*.mp. |
| 103 | inequalit*.mp. |
| 104 | inequit*.mp. |
| 105 | equity.mp. |
| 106 | deprivation.mp. |
| 107 | gini.mp. |
| 108 | concentration index.mp. |
| 109 | social welfare/ |
| 110 | social class/ |
| 111 | poverty/ |
| 112 | social status/ |
| 113 | social background/ |
| 114 | social class*.mp. |
| 115 | social determinants.mp. |
| 116 | social status.mp. |
| 117 | social position.mp. |
| 118 | social background.mp. |
| 119 | social circumstance*.mp. |
| 120 | socio-economic.mp. |
| 121 | socioeconomic.mp. |
| 122 | sociodemographic.mp. |
| 123 | socio-demographic.mp. |
| 124 | SES.mp. |
| 125 | disadvantaged.mp. |
| 126 | impoverished.mp. |
| 127 | poverty.mp. |
| 128 | economic level.mp. |
| 129 | assets index.mp. |
| 130 | income*.mp. |
| 131 | or/97-130 |
| 132 | exp social isolation/ |
| 133 | social capital/ |
| 134 | social stigma/ |
| 135 | social support/ |
| 136 | social environment/ |
| 137 | trust/ |
| 138 | exp social exclusion/ |
| 139 | anomie/ |
| 140 | social participation/ |
| 141 | social exclusion.mp. |
| 142 | (social adj (capital or cohes* or organis* or organiz*)).mp. |
| 143 | (community adj3 (cohes* or participa*)).mp. |
| 144 | ((neighbourhood or neighborhood) adj cohes*).mp. |
| 145 | social relationships.mp. |
| 146 | social network*.mp. |
| 147 | collective efficacy.mp. |
| 148 | civil society.mp. |
| 149 | informal social control.mp. |
| 150 | neighbo*rhood disorder.mp. |
| 151 | social disorgani?ation.mp. |
| 152 | anomie.mp. |
| 153 | social support.mp. |
| 154 | social participation.mp. |
| 155 | trust.mp. |
| 156 | emotional support.mp. |
| 157 | psychosocial support.mp. |
| 158 | community capital.mp. |
| 159 | neighbo*rhood cohesion.mp. |
| 160 | social influence.mp. |
| 161 | (soci*context* or soci*-context*).mp. |
| 162 | or/132-161 |
| 163 | health disparity/ |
| 164 | health equity/ |
| 165 | health care access/ |
| 166 | health*care disparit*.mp. |
| 167 | health care disparit*.mp. |
| 168 | health status disparit*.mp. |
| 169 | health disparit*.mp. |
| 170 | health inequalit*.mp. |
| 171 | health inequit*.mp. |
| 172 | medically underserved.mp. |
| 173 | or/163-172 |
| 174 | 32 or 75 or 93 or 131 or 162 or 173 |
| 175 | potential determinants.mp. |
| 176 | significant correlates of.mp. |
| 177 | (independent correlates or independent association*).mp. |
| 178 | variables associated with.mp. |
| 179 | determinants of.mp. |
| 180 | factors associated with.mp. |
| 181 | identif* determinants.mp. |
| 182 | (more likely or less likely or just as likely).mp. |
| 183 | risk factors for.mp. |
| 184 | (significantly related to or significant predictor).mp. |
| 185 | (also adj2 associated with).mp. |
| 186 | (at increased risk or at decreased risk).mp. |
| 187 | association* between.mp. |
| 188 | (positively associated or negatively associated).mp. |
| 189 | differed by.mp. |
| 190 | (were high* amongst or were low* amongst).mp. |
| 191 | (inverse relationship with or inversely associated with or inversely related to).mp. |
| 192 | reverse association.mp. |
| 193 | differentially affects.mp. |
| 194 | evidence of a link between.mp. |
| 195 | (significantly adj3 likelihood of).mp. |
| 196 | protective factors for.mp. |
| 197 | (differ* adj2 according to).mp. |
| 198 | (inverse adj2 gradient).mp. |
| 199 | (positive adj2 gradient).mp. |
| 200 | (negative adj2 gradient).mp. |
| 201 | (trends were adj3 across).mp. |
| 202 | (related to adj3 variable*).mp. |
| 203 | (differences were adj3 explained by).mp. |
| 204 | (significant among or no# significant among).mp. |
| 205 | or/175-204 |
| 206 | 174 or 205 |
| 207 | Hospitalization/ |
| 208 | Patient Readmission/ |
| 209 | Patient Admission/ |
| 210 | ((emergency or unplanned or unanticipated or unexpected or avoid*) adj3 (admission* or readmission* or hospitali#ation*)).ti,ab,kw. |
| 211 | (overnight stay adj3 admission*).ti,ab,kw. |
| 212 | (primary care adj3 admission*).ti,ab,kw. |
| 213 | (ambulatory care adj3 admission*).ti,ab,kw. |
| 214 | or/207-213 |
| 215 | ((program* or policy or policies or strateg* or scheme* or intervention* or project* or initiative*) adj5 (evaluat* or effect* or measur* or assess* or experiment* or impact*)).ti,ab,kw. |
| 216 | (comparative study or controlled clinical trial or evaluation studies or meta analysis or pragmatic clinical trial or randomized controlled trial or "systematic review").ti,ab. |
| 217 | exp Clinical Trial/ or exp Randomized Controlled Trial/ or exp Randomization/ or Random Allocation/ or Double-Blind Method/ or Single-Blind Method/ or exp Cross-Over Studies/ or Program Evaluation/ |
| 218 | (RCT or randomi* or nonrandomi* or non randomi* or quasiexperiment* or quasi experiment* or quasirandomi* or quasi randomi* or pseudoexperiment* or pseudo experiment* or pseudorandomi* or pseudo randomi* or natural experiment* or pretest or pre test or posttest or post test or time series or repeat* measure* or systematic review*).ti,ab,kw. |
| 219 | (before adj1 after adj1 (stud* or trial* or design*)).ti,ab. |
| 220 | or/215-219 |
| 221 | 214 and 220 |
| 222 | (Algeria* or Egypt* or Liby* or Morocc* or Tunisia* or Western Sahara* or Angola* or Benin or Botswana* or Burkina Faso or Burundi or Cameroon or Cape Verde or Central African Republic or Chad or Comoros or Congo or Djibouti or Eritrea or Ethiopia* or Gabon or Gambia* or Ghana or Guinea or Keny* or Lesotho or Liberia or Madagasca* or Malawi or Mali or Mauritania or Mauritius or Mayotte or Mozambiq* or Namibia* or Niger or Nigeria* or Reunion or Rwand* or Saint Helena or Senegal or Seychelles or Sierra Leone or Somalia or South Africa* or Sudan or Swaziland or Tanzania or Togo or Ugand* or Zambia* or Zimbabw* or China or Chinese or Hong Kong or Macao or Mongolia* or Taiwan* or Belarus or Moldov* or Russia* or Ukraine or Afghanistan or Armenia* or Azerbaijan or Bahrain or Cyprus or Cypriot or Georgia* or Iran* or Iraq* or Israel* or Jordan* or Kazakhstan or Kuwait or Kyrgyzstan or Leban* or Oman or Pakistan* or Palestin* or Qatar or Saudi Arabia or Syria* or Tajikistan or Turkmenistan or United Arab Emirates or Uzbekistan or Yemen or Bangladesh* or Bhutan or British Indian Ocean Territory or Brunei Darussalam or Cambodia* or India* or Indonesia* or Lao or People's Democratic Republic or Malaysia* or Maldives or Myanmar or Nepal or Philippin* or Singapore or Sri Lanka or Thai* or Timor Leste or Vietnam or Albania* or Andorra or Bosnia* or Herzegovina* or Bulgaria* or Croatia* or Estonia or Faroe Islands or Greenland or Liechtenstein or Lithuani* or Macedonia or Malta or maltese or Romania or Serbia* or Montenegro or Slovenia or Svalbard or Argentina* or Belize or Bolivia* or Brazil* or chile or Chilean or Colombia* or Costa Rica* or Cuba or Ecuador or El Salvador or French Guiana or Guatemala* or Guyana or Haiti or Honduras or Jamaica* or Nicaragua* or Panama or Paraguay or Peru or Puerto Rico or Suriname or Uruguay or Venezuela or developing countr* or south America*).ti,sh. |
| 223 | 221 not 222 |
| 224 | limit 223 to (english language and humans and yr="1999 -Current") |
| 225 | 206 and 224 |

**CINAHL search strategy**

| S296 | S38 AND S293 |
| --- | --- |
| S295 | S38 AND S293 |
| S294 | S38 AND S293 |
| S293 | S231 OR S292 |
| S292 | S232 OR S233 OR S234 OR S235 OR S236 OR S237 OR S238 OR S239 OR S240 OR S241 OR S242 OR S243 OR S244 OR S245 OR S246 OR S248 OR S249 OR S250 OR S251 OR S252 OR S253 OR S254 OR S255 OR S256 OR S257 OR S258 OR S259 OR S260 OR S261 OR S262 OR S263 OR S264 OR S265 OR S266 OR S267 OR S268 OR S269 OR S270 OR S271 OR S272 OR S273 OR S274 OR S275 OR S276 OR S277 OR S278 OR S279 OR S280 OR S281 OR S282 OR S283 OR S284 OR S285 OR S286 OR S287 OR S288 OR S289 OR S290 OR S291 |
| S291 | TI "risk factors for" |
| S290 | AB ("significant among" or "no# significant among") |
| S289 | TI ("significant among" or "no# significant among") |
| S288 | AB (differences were N3 explained by) |
| S287 | TI (differences were N3 explained by) |
| S286 | AB (related to N3 variable*) |
| S285 | TI (related to N3 variable*) |
| S284 | AB (trends were N3 across) |
| S283 | TI (trends were N3 across) |
| S282 | AB (negative N2 gradient) |
| S281 | TI (negative N2 gradient) |
| S280 | AB (positive N2 gradient) |
| S279 | TI (positive N2 gradient) |
| S278 | AB (inverse N2 gradient) |
| S277 | TI (inverse N2 gradient) |
| S276 | AB (differ* N2 according to) |
| S275 | TI (differ* N2 according to) |
| S274 | AB "protective factors for" |
| S273 | TI "protective factors for" |
| S272 | AB (significantly N3 likelihood of) |
| S271 | TI (significantly N3 likelihood of) |
| S270 | AB "evidence of a link between" |
| S269 | TI "evidence of a link between" |
| S268 | AB "differentially affects" |
| S267 | TI "differentially affects" |
| S266 | AB "reverse association" |
| S265 | TI "reverse association" |
| S264 | AB "inverse relationship with" or "inversely associated with" or "inversely related to" |
| S263 | TI "inverse relationship with" or "inversely associated with" or "inversely related to" |
| S262 | AB "were high* amongst" or "were low* amongst" |
| S261 | TI "were high* amongst" or "were low* amongst" |
| S260 | AB "differed by" |
| S259 | TI "differed by" |
| S258 | AB (positively associated or negatively associated) |
| S257 | TI (positively associated or negatively associated) |
| S256 | AB "association* between" |
| S255 | TI "association* between" |
| S254 | AB (at increased risk or at decreased risk) |
| S253 | TI (at increased risk or at decreased risk) |
| S252 | AB (also N2 associated with) |
| S251 | TI (also N2 associated with) |
| S250 | AB (significantly related to or significant predictor) |
| S249 | TI (significantly related to or significant predictor) |
| S248 | AB "risk factors for" |
| S247 | AB (more likely or less likely or just as likely) |
| S246 | TI (more likely or less likely or just as likely) |
| S245 | AB "identif* determinants" |
| S244 | TI "identif* determinants" |
| S243 | AB "factors associated with" |
| S242 | TI "factors associated with" |
| S241 | AB "determinants of" |
| S240 | TI "determinants of" |
| S239 | AB "variables associated with" |
| S238 | TI "variables associated with" |
| S237 | AB (independent correlates or independent association*) |
| S236 | TI (independent correlates or independent association*) |
| S235 | AB "significant correlates of" |
| S234 | TI "significant correlates of" |
| S233 | AB "potential determinants" |
| S232 | TI "potential determinants" |
| S231 | S90 OR S95 OR S107 OR S160 OR S210 OR S230 |
| S230 | S211 OR S212 OR S213 OR S214 OR S215 OR S216 OR S217 OR S218 OR S219 OR S220 OR S221 OR S222 OR S223 OR S224 OR S225 OR S226 OR S227 OR S228 OR S229 |
| S229 | (MH "Medically Underserved Area") |
| S228 | (MH "Medically Underserved") |
| S227 | AB medically underserved |
| S226 | TI medically underserved |
| S225 | AB health inequit* |
| S224 | TI health inequit* |
| S223 | AB health inequalit* |
| S222 | TI health inequalit* |
| S221 | AB health disparit* |
| S220 | TI health disparit* |
| S219 | AB health status disparit* |
| S218 | TI health status disparit* |
| S217 | AB health care disparit* |
| S216 | TI health care disparit* |
| S215 | AB health*care disparit* |
| S214 | TI health*care disparit* |
| S213 | (MH "Health Services Accessibility") |
| S212 | (MH "Healthcare Disparities") |
| S211 | (MH "Health Status Disparities") |
| S210 | S161 OR S162 OR S163 OR S164 OR S165 OR S166 OR S167 OR S168 OR S169 OR S170 OR S171 OR S172 OR S173 OR S174 OR S175 OR S176 OR S177 OR S178 OR S179 OR S180 OR S181 OR S182 OR S183 OR S184 OR S185 OR S186 OR S187 OR S188 OR S189 OR S190 OR S191 OR S192 OR S193 OR S194 OR S195 OR S196 OR S197 OR S198 OR S199 OR S200 OR S201 OR S202 OR S203 OR S204 OR S205 OR S206 OR S207 OR S208 OR S209 |
| S209 | AB (soci*context* or soci*-context*) |
| S208 | TI (soci*context* or soci*-context*) |
| S207 | AB social influence |
| S206 | TI social influence |
| S205 | AB neighbo*rhood cohesion |
| S204 | TI neighbo*rhood cohesion |
| S203 | AB community capital |
| S202 | TI community capital |
| S201 | AB psychosocial support |
| S200 | TI psychosocial support |
| S199 | AB emotional support |
| S198 | TI emotional support |
| S197 | AB trust |
| S196 | TI trust |
| S195 | AB social participation |
| S194 | TI social participation |
| S193 | AB social support |
| S192 | TI social support |
| S191 | AB anomie |
| S190 | TI anomie |
| S189 | AB social disorgani?ation |
| S188 | TI social disorgani?ation |
| S187 | AB neighbo*rhood disorder |
| S186 | TI neighbo*rhood disorder |
| S185 | AB informal social control |
| S184 | TI informal social control |
| S183 | AB civil society |
| S182 | TI civil society |
| S181 | AB collective efficacy |
| S180 | TI collective efficacy |
| S179 | AB social network* |
| S178 | TI social network* |
| S177 | AB social relationships |
| S176 | TI social relationships |
| S175 | AB ((neighbourhood or neighborhood) N0 cohes*). |
| S174 | TI ((neighbourhood or neighborhood) N0 cohes*). |
| S173 | AB (community N0 (cohes* or participa*)) |
| S172 | TI (community N0 (cohes* or participa*)) |
| S171 | AB (social N0 (capital or cohes* or organis* or organiz*)) |
| S170 | TI (social N0 (capital or cohes* or organis* or organiz*)) |
| S169 | AB social exclusion |
| S168 | TI social exclusion |
| S167 | (MH "Social Participation") |
| S166 | (MH "Social Isolation") |
| S165 | (MH "Trust") |
| S164 | (MH "Social Environment+") |
| S163 | (MH "Social Control") |
| S162 | (MH "Social Capital") |
| S161 | (MH "Stigma") |
| S160 | S108 OR S109 OR S110 OR S111 OR S112 OR S113 OR S114 OR S115 OR S116 OR S117 OR S118 OR S119 OR S120 OR S121 OR S122 OR S123 OR S124 OR S125 OR S126 OR S127 OR S128 OR S129 OR S130 OR S131 OR S132 OR S133 OR S134 OR S135 OR S136 OR S137 OR S138 OR S139 OR S140 OR S141 OR S142 OR S143 OR S144 OR S145 OR S146 OR S147 OR S148 OR S149 OR S150 OR S151 OR S152 OR S153 OR S154 OR S155 OR S156 OR S157 OR S158 OR S159 |
| S159 | AB income* |
| S158 | TI income* |
| S157 | AB assets index |
| S156 | TI assets index |
| S155 | AB economic level |
| S154 | TI economic level |
| S153 | AB poverty |
| S152 | TI poverty |
| S151 | AB impoverished |
| S150 | TI impoverished |
| S149 | AB disadvantaged |
| S148 | TI disadvantaged |
| S147 | AB SES |
| S146 | TI SES |
| S145 | AB socio-demographic |
| S144 | TI socio-demographic |
| S143 | AB sociodemographic |
| S142 | TI sociodemographic |
| S141 | AB socioeconomic |
| S140 | TI socioeconomic |
| S139 | AB socio-economic |
| S138 | TI socio-economic |
| S137 | AB social circumstance* |
| S136 | TI social circumstance* |
| S135 | AB social background |
| S134 | TI social background |
| S133 | AB social position |
| S132 | TI social position |
| S131 | AB social status |
| S130 | TI social status |
| S129 | AB social determinants |
| S128 | TI social determinants |
| S127 | AB Social class* |
| S126 | TI Social class* |
| S125 | (MH "Social Welfare") |
| S124 | (MH "Socioeconomic Factors+") |
| S123 | AB concentration index |
| S122 | TI concentration index |
| S121 | AB gini |
| S120 | TI gini |
| S119 | AB deprivation |
| S118 | TI deprivation |
| S117 | AB equity |
| S116 | TI equity |
| S115 | AB inequit* |
| S114 | TI inequit* |
| S113 | AB inequalit* |
| S112 | TI inequalit* |
| S111 | AB disparit* |
| S110 | TI disparit* |
| S109 | (MH "Psychosocial Deprivation") |
| S108 | (MH "Social Determinants of Health") |
| S107 | S96 OR S97 OR S98 OR S99 OR S100 OR S101 OR S102 OR S103 OR S104 OR S105 OR S106 |
| S106 | AB ((higher or better or worse or less) N0 level? of education) |
| S105 | TI ((higher or better or worse or less) N0 level? of education) |
| S104 | AB ((higher or better or worse or less) N0 educated) |
| S103 | TI ((higher or better or worse or less) N0 educated) |
| S102 | AB education* N2 level? |
| S101 | TI education* N2 level? |
| S100 | AB educational status |
| S99 | TI educational status |
| S98 | AB schooling |
| S97 | TI schooling |
| S96 | (MH "Education") |
| S95 | S91 OR S92 OR S93 OR S94 |
| S94 | AB unemployment |
| S93 | TI unemployment |
| S92 | AB occupations |
| S91 | TI occupations |
| S90 | S39 OR S40 OR S41 OR S42 OR S43 OR S44 OR S45 OR S46 OR S47 OR S48 OR S49 OR S50 OR S51 OR S52 OR S53 OR S54 OR S55 OR S56 OR S57 OR S58 OR S59 OR S60 OR S61 OR S62 OR S63 OR S64 OR S65 OR S66 OR S67 OR S68 OR S69 OR S70 OR S71 OR S72 OR S73 OR S74 OR S75 OR S76 OR S77 OR S78 OR S79 OR S80 OR S81 OR S82 OR S83 OR S84 OR S85 OR S86 OR S87 OR S88 OR S89 |
| S89 | AB (widow* or cohabit* or divorce* or single parent* or live* alone) |
| S88 | TI (widow* or cohabit* or divorce* or single parent* or live* alone) |
| S87 | AB marital status or marriage status |
| S86 | TI marital status or marriage status |
| S85 | AB (household N2 size) |
| S84 | TI (household N2 size) |
| S83 | AB (living N1 (outside or inside or near* or adjacent)) |
| S82 | TI (living N1 (outside or inside or near* or adjacent)) |
| S81 | AB overcrowding |
| S80 | TI overcrowding |
| S79 | AB mortgage debt* |
| S78 | TI mortgage debt* |
| S77 | AB mortgage arrears |
| S76 | TI mortgage arrears |
| S75 | AB mortgage delinquency |
| S74 | TI mortgage delinquency |
| S73 | AB (repossess* N3 propert*). |
| S72 | TI (repossess* N3 propert*). |
| S71 | AB (repossess* N3 hous*) |
| S70 | TI (repossess* N3 hous*) |
| S69 | AB home ownership |
| S68 | TI home ownership |
| S67 | AB home repossession* |
| S66 | TI home repossession* |
| S65 | AB housing loss |
| S64 | TI housing loss |
| S63 | AB eviction* |
| S62 | TI eviction* |
| S61 | AB foreclosure |
| S60 | TI foreclosure |
| S59 | AB mortgage problems |
| S58 | TI mortgage problems |
| S57 | TI housing security |
| S56 | AB housing security |
| S55 | AB housing strain |
| S54 | TI housing strain |
| S53 | AB housing insecurity |
| S52 | TI housing insecurity |
| S51 | AB housing instability |
| S50 | TI housing instability |
| S49 | AB inner?city |
| S48 | TI inner?city |
| S47 | AB rural* |
| S46 | TI rural* |
| S45 | AB residential environment* |
| S44 | TI residential environment* |
| S43 | AB NEIGHBO?RHOOD* |
| S42 | TI NEIGHBO?RHOOD* |
| S41 | SU NEIGHBO?RHOOD* |
| S40 | (MH "Marital Status+") |
| S39 | (MH "Residence Characteristics+") |
| S38 | S33 NOT S36 |
| S37 | S33 NOT S36 |
| S36 | S34 OR S35 |
| S35 | SU Algeria* or Egypt* or Liby* or Morocc* or Tunisia* or Western Sahara* or Angola* or Benin or Botswana* or Burkina Faso or Burundi or Cameroon or Cape Verde or Central African Republic or Chad or Comoros or Congo or Djibouti or Eritrea or Ethiopia* or Gabon or Gambia* or Ghana or Guinea or Keny* or Lesotho or Liberia or Madagasca* or Malawi or Mali or Mauritania or Mauritius or Mayotte or Mozambiq* or Namibia* or Niger or Nigeria* or Reunion or Rwand* or Saint Helena or Senegal or Seychelles or Sierra Leone or Somalia or South Africa* or Sudan or Swaziland or Tanzania or Togo or Ugand* or Zambia* or Zimbabw* or China or Chinese or Hong Kong or Macao or Mongolia* or Taiwan* or Belarus or Moldov* or Russia* or Ukraine or Afghanistan or Armenia* or Azerbaijan or Bahrain or Cyprus or Cypriot or Georgia* or Iran* or Iraq* or Israel* or Jordan* or Kazakhstan or Kuwait or Kyrgyzstan or Leban* or Oman or Pakistan* or Palestin* or Qatar or Saudi Arabia or Syria* or Tajikistan or Turkmenistan or United Arab Emirates or Uzbekistan or Yemen or Bangladesh* or Bhutan or British Indian Ocean Territory or Brunei Darussalam or Cambodia* or India* or Indonesia* or Lao or People's Democratic Republic or Malaysia* or Maldives or Myanmar or Nepal or Philippin* or Singapore or Sri Lanka or Thai* or Timor Leste or Vietnam or Albania* or Andorra or Bosnia* or Herzegovina* or Bulgaria* or Croatia* or Estonia or Faroe Islands or Greenland or Liechtenstein or Lithuani* or Macedonia or Malta or maltese or Romania or Serbia* or Montenegro or Slovenia or Svalbard or Argentina* or Belize or Bolivia* or Brazil* or chile or Chilean or Colombia* or Costa Rica* or Cuba or Ecuador or El Salvador or French Guiana or Guatemala* or Guyana or Haiti or Honduras or Jamaica* or Nicaragua* or Panama or Paraguay or Peru or Puerto Rico or Suriname or Uruguay or Venezuela or developing countr* or south America* |
| S34 | TI Algeria* or Egypt* or Liby* or Morocc* or Tunisia* or Western Sahara* or Angola* or Benin or Botswana* or Burkina Faso or Burundi or Cameroon or Cape Verde or Central African Republic or Chad or Comoros or Congo or Djibouti or Eritrea or Ethiopia* or Gabon or Gambia* or Ghana or Guinea or Keny* or Lesotho or Liberia or Madagasca* or Malawi or Mali or Mauritania or Mauritius or Mayotte or Mozambiq* or Namibia* or Niger or Nigeria* or Reunion or Rwand* or Saint Helena or Senegal or Seychelles or Sierra Leone or Somalia or South Africa* or Sudan or Swaziland or Tanzania or Togo or Ugand* or Zambia* or Zimbabw* or China or Chinese or Hong Kong or Macao or Mongolia* or Taiwan* or Belarus or Moldov* or Russia* or Ukraine or Afghanistan or Armenia* or Azerbaijan or Bahrain or Cyprus or Cypriot or Georgia* or Iran* or Iraq* or Israel* or Jordan* or Kazakhstan or Kuwait or Kyrgyzstan or Leban* or Oman or Pakistan* or Palestin* or Qatar or Saudi Arabia or Syria* or Tajikistan or Turkmenistan or United Arab Emirates or Uzbekistan or Yemen or Bangladesh* or Bhutan or British Indian Ocean Territory or Brunei Darussalam or Cambodia* or India* or Indonesia* or Lao or People's Democratic Republic or Malaysia* or Maldives or Myanmar or Nepal or Philippin* or Singapore or Sri Lanka or Thai* or Timor Leste or Vietnam or Albania* or Andorra or Bosnia* or Herzegovina* or Bulgaria* or Croatia* or Estonia or Faroe Islands or Greenland or Liechtenstein or Lithuani* or Macedonia or Malta or maltese or Romania or Serbia* or Montenegro or Slovenia or Svalbard or Argentina* or Belize or Bolivia* or Brazil* or chile or Chilean or Colombia* or Costa Rica* or Cuba or Ecuador or El Salvador or French Guiana or Guatemala* or Guyana or Haiti or Honduras or Jamaica* or Nicaragua* or Panama or Paraguay or Peru or Puerto Rico or Suriname or Uruguay or Venezuela or developing countr* or south America* |
| S33 | S16 AND S32 |
| S32 | S17 OR S18 OR S19 OR S20 OR S21 OR S22 OR S23 OR S24 OR S25 OR S26 OR S27 OR S28 OR S29 OR S30 OR S31 |
| S31 | AB before N1 after N1 (stud* or trial* or design*) |
| S30 | TI before N1 after N1 (stud* or trial* or design*) |
| S29 | TI RCT or randomi* or nonrandomi* or non randomi* or quasiexperiment* or quasi experiment* or quasirandomi* or quasi randomi* or pseudoexperiment* or pseudo experiment* or pseudorandomi* or pseudo randomi* or natural experiment* or pretest or pre test or posttest or post test or time series or repeat* measure* or systematic review* |
| S28 | AB RCT or randomi* or nonrandomi* or non randomi* or quasiexperiment* or quasi experiment* or quasirandomi* or quasi randomi* or pseudoexperiment* or pseudo experiment* or pseudorandomi* or pseudo randomi* or natural experiment* or pretest or pre test or posttest or post test or time series or repeat* measure* or systematic review* |
| S27 | (MH "Health Services Research+") |
| S26 | (MH "Quasi-Experimental Studies+") |
| S25 | (MH "Nonexperimental Studies+") |
| S24 | (MH "Experimental Studies+") |
| S23 | (MH "Policy Studies+") |
| S22 | (MH "Epidemiological Research") |
| S21 | (MH "Evaluation Research") |
| S20 | (MH "Ecological Research") |
| S19 | (MH "Comparative Studies") |
| S18 | TI program* or policy or policies or strateg* or scheme* or intervention* or project* or initiative*) N5 (evaluat* or effect* or measur* or assess* or experiment* or impact*) |
| S17 | AB program* or policy or policies or strateg* or scheme* or intervention* or project* or initiative*) N5 (evaluat* or effect* or measur* or assess* or experiment* or impact*) |
| S16 | S1 OR S2 OR S3 OR S4 OR S5 OR S6 OR S7 OR S8 OR S9 OR S10 OR S11 OR S12 OR S13 OR S14 OR S15 |
| S15 | AB ambulatory care N3 admission* |
| S14 | AB primary care N3 admission* |
| S13 | AB overnight stay N3 admission* |
| S12 | AB (emergency or unplanned or unanticipated or unexpected or avoid*) N3 (admission* or readmission* or hospitali#ation*) |
| S11 | (MH "Aged, Hospitalized") |
| S10 | (MH "Adolescent, Hospitalized") |
| S9 | (MH "Child, Hospitalized") |
| S8 | (MH "Infant, Hospitalized") |
| S7 | TI ambulatory care N3 admission* |
| S6 | TI primary care N3 admission* |
| S5 | TI overnight stay N3 admission* |
| S4 | TI (emergency or unplanned or unanticipated or unexpected or avoid*) N3 (admission* or readmission* or hospitali#ation*) |
| S3 | (MH "Readmission") |
| S2 | (MH "Patient Admission") |
| S1 | (MH "Hospitalization") |

**Web of Knowledge search strategy**

|  | TS=(hospitali?ation* OR “patient readmission*” OR “patient admission*” OR (emergency or unplanned or unanticipated or unexpected or avoid*) NEAR/3 (admission* or readmission* or hospitali?ation*) OR (“overnight stay” NEAR/3 admission*) OR (“primary care” NEAR/3 admission*) OR (“ambulatory care” NEAR/3 admission*))  *****  TS=((program* or policy or policies or strateg* or scheme* or intervention* or project* or initiative*) NEAR/5 (evaluat* or effect* or measur* or assess* or experiment* or impact*))  OR  TS=(“comparative study” OR “controlled clinical trial” OR “evaluation studies” OR “meta analysis” OR “pragmatic clinical trial” OR “randomized controlled trial” OR "systematic review" OR RCT OR randomi* OR nonrandomi* OR “non randomi*” OR quasiexperiment* OR “quasi experiment*” OR quasirandomi* OR “quasi randomi*” OR pseudoexperiment* OR “pseudo experiment*” OR pseudorandomi* OR “pseudo randomi*” OR “natural experiment*” OR pretest OR “pre test” OR posttest OR “post test” OR “time series” OR “repeat* measure*” OR “systematic review*” OR “before after stud*” OR “before after trial*” OR “before after design*”)  *******  TS=(before NEXT/1 after NEXT/1 (stud* or trial* or design*))  To  “before after stud*” OR “before after trial*” OR “before after design*”)  ********  CU=(Algeria* or Egypt* or Liby* or Morocc* or Tunisia* or “Western Sahara*” or Angola* or Benin or Botswana* or Burkina Faso or Burundi or Cameroon or Cape Verde or “Central African Republic” or Chad or Comoros or Congo or Djibouti or Eritrea or Ethiopia* or Gabon or Gambia* or Ghana or Guinea or Keny* or Lesotho or Liberia or Madagasca* or Malawi or Mali or Mauritania or Mauritius or Mayotte or Mozambiq* or Namibia* or Niger or Nigeria* or Reunion or Rwand* or “Saint Helena” or Senegal or Seychelles or “Sierra Leone” or Somalia or “South Africa*” or Sudan or Swaziland or Tanzania or Togo or Ugand* or Zambia* or Zimbabw* or China or Chinese or “Hong Kong” or Macao or Mongolia* or Taiwan* or Belarus or Moldov* or Russia* or Ukraine or Afghanistan or Armenia* or Azerbaijan or Bahrain or Cyprus or Cypriot or Georgia* or Iran* or Iraq* or Israel* or Jordan* or Kazakhstan or Kuwait or Kyrgyzstan or Leban* or Oman or Pakistan* or Palestin* or Qatar or Saudi Arabia or Syria* or Tajikistan or Turkmenistan or “United Arab Emirates” or Uzbekistan or Yemen or Bangladesh* or Bhutan or “British Indian Ocean Territory” or Brunei Darussalam or Cambodia* or India* or Indonesia* or Lao or “People's Democratic Republic or Malaysia*” or Maldives or Myanmar or Nepal or Philippin* or Singapore or “Sri Lanka” or Thai* or “Timor Leste” or Vietnam or Albania* or Andorra or Bosnia* or Herzegovina* or Bulgaria* or Croatia* or Estonia or Faroe Islands or Greenland or Liechtenstein or Lithuani* or Macedonia or Malta or maltese or Romania or Serbia* or Montenegro or Slovenia or Svalbard or Argentina* or Belize or Bolivia* or Brazil* or chile or Chilean or Colombia* or Costa Rica* or Cuba or Ecuador or “El Salvador” or “French Guiana” or Guatemala* or Guyana or Haiti or Honduras or Jamaica* or Nicaragua* or Panama or Paraguay or Peru or “Puerto Rico” or Suriname or Uruguay or Venezuela or “developing countr*” or “south America*”  LINE 8  TS=("Residence characteristics" OR "marital status" OR neighbo?rhood* OR "residential environment" OR rural* OR “inner*city” OR "housing instability" OR "housing insecurity" OR "housing strain" OR "housing security" OR "mortgage problems" OR foreclosure OR eviction* OR "housing loss" OR "home repossession*" OR "home ownership" OR (repossess* NEAR/3 hous*) OR (repossess* NEAR/3 propert*) OR ("mortgage delinquency") OR "mortgage arrears" OR "mortgage debt*" OR "overcrowding" OR (living NEAR/1 (outside or inside or near* or adjacent)) OR (household NEAR/2 size) OR "marital status" OR "marriage status" OR (widow* or cohabit* or divorce* or single parent* or live* alone))  LINE 9  TS=(Occupations OR Unemployment OR occupations OR unemployment)  LINE 10  TS=(“Educational status” OR Education OR Schooling OR “educational status” OR (education* NEXT/2 level) OR ((higher or better or worse or less) NEXT educated) OR ((higher or better or worse or less) NEXT “level* of education”))  LINE 11  TS=( Social determinants of Health OR “Psychosocial Deprivation” OR “Sociological Factors” OR “Working Poor” OR “Hierarchy Social” OR disparit* OR inequalit* OR inequit* OR equity OR deprivation OR Gini OR “concentration index” OR “Socioeconomic Factors” OR “Social Welfare” OR “Social class” OR Poverty OR Income OR “Social class*” OR “Social position” OR “Social background” OR “Social circumstance*” or “Socio-economic” or “Socioeconomic” or “Sociodemographic” OR “Socio-demographic” OR “SES” or “disadvantaged” or “Impoverished” or “poverty” of “economic level” or “assets index” or “income*”)  LINE 12  TS=(“Social stigma” OR “social capital” or “Social Control” or “Social support” or “Social Environment” or “trust” or “social conditions” or “social isolation” of social marginalization” or “anomie” or “social participation” or “social exclusion” or (social adj (capital or cohes* or organis* or organiz*)) or (community adj3 (cohes* or participa*)) or ((neighbourhood or neighborhood) adj cohes*) or “social relationships” or “social network*” or “collective efficacy” or “civil society£ or “informal social control” or “neighbo*rhood disorder” OR “social disorgani?ation” OR “emotional support” or “psychosocial support” or “community capital” or “social influence” or soci*context* or soci*-context*)  LINE 13  TS=(“Health Status Disparities”” OR “Health Services Accessibility” OR “Health Equity” OR “health*care disparit*” OR “health care disparit*” OR “health status disparit*” OR “health disparit*” OR “health inequalit*” OR “health inequit* OR “medically underserved”)  LINE 14  TS=(“potential determinants” OR “significant correlates of” OR “independent correlates” or “independent association*” OR “variables associated with” OR “determinants of” OR “factors associated with” or “identif* determinants” or “more likely” or “less likely” or “just as likely” or “risk factors” or “significantly related to” or “significant predictor” or “also adj2 associated with” OR “at increased risk” OR “at decreased risk” OR “association* between” OR “positively associated” or “negatively associated” OR “differed by” OR “were high* amongst” or “were low* amongst” OR “inverse relationship with” or “inversely associated with” or “inversely related to” OR “reverse association” OR “differentially affects” OR “evidence of a link between” OR “significantly adj3 likelihood of” OR “protective factors for” OR (“differ* NEXT/2 “according to”) OR (inverse NEXT/2 gradient) OR “positive NEXT/2 gradient) OR (negative NEXT/2 gradient) OR (trends were NEXT/3 across) OR (“related to” NEXT/3 variable*) OR (“differences were” NEXT/3 “explained by”). OR “significant among” or “no# significant among”) |
| --- | --- |
