# Supplementary material for: What works to reduce socioeconomic inequalities in hospitalisations and readmissions? Systematic review of the equity impacts of population-level, health service and integrative interventions
Source: BMJ Public Health. 2025 Sep 23;3(2):e002595. doi: 10.1136/bmjph-2025-002595 (PMC12458884; doi:10.1136/bmjph-2025-002595)
Supplement: online supplemental file 3 [file bmjph-3-2-s003.docx]

**Supplementary file 3: Reasons for exclusion after full-text review**

Full text screened: n=563

Included studies: n=36

Exclude on study type: n=155

Exclude on outcome: n=231

Exclude on study period: n=4

Exclude on targeted intervention: n=137

Exclude on study type: n=155

| Crawford (2021) | A systematic review examining the clinical and health-care outcomes for congenital heart disease patients using home monitoring programmes | 2021 |
| --- | --- | --- |
| Adams (2014) | Implementation of the Re-Engineered Discharge (RED) toolkit to decrease all-cause readmission rates at a rural community hospital. | 2014 |
| Agarwal (2019) | Design and rationale for a pragmatic cluster randomized trial of the Cardiovascular Health Awareness Program (CHAP) for social housing residents in Ontario and Quebec, Canada | 2019 |
| Ajmera (2015) | Real-world observational study of association between statin medications and COPD-specific outcomes | 2015 |
| Al-Khazaali (2016) | Effective Strategies in Reducing Rehospitalizations in Patients With Heart Failure. | 2016 |
| Al-Lami (2019) | Testosterone Replacement Therapy and Rehospitalization in Older Men With Testosterone Deficiency in a Postacute Care Setting. | 2019 |
| Al-Shehhi (2017) | Rate and predictors of 1-year readmission in tertiary psychiatric hospitals | 2017 |
| Albritton (2018) | The Effect Of The Hospital Readmissions Reduction Program On Readmission And Observation Stay Rates For Heart Failure. | 2018 |
| Alcain (2019) | THE EFFECT OF A NURSE-DRIVEN PROGRAM UTILIZING IMPLANTABLE PULMONARY ARTERY PRESSURE MONITORING TO REDUCE HOSPITALIZATIONS IN LOW-SOCIOECONOMIC URBAN PATIENTS WITH HEART FAILURE | 2019 |
| Alende-Castro (2018) | Q fever in Spain: Description of a new series, and systematic review. | 2018 |
| Altice (2020) | Early Cardiac Rehab to Reduce Heart Failure Readmissions. | 2020 |
| Ancona (2004) | Differences in access to coronary care unit among patients with acute myocardial infarction in Rome: old, ill, and poor people hold the burden of inefficiency. | 2004 |
| Angelelli (2002) | Access to postacute nursing home care before and after the BBA. Balanced Budget Act. | 2002 |
| Anonymous (2019) | The Effect of a Nurse-Driven Program Utilizing Implantable Pulmonary Artery Pressure Monitoring to Reduce Hospitalizations in Low-Socioeconomic Urban Patients with Heart Failure | 2019 |
| Apter (2017) | A patient advocate to facilitate access and improve communication, care, and outcomes in adults with moderate or severe asthma: Rationale, design, and methods of a randomized controlled trial | 2017 |
| Apter (2022) | Clinic navigation and home visits to improve asthma care in low income adults with poorly controlled asthma: Before and during the pandemic. | 2022 |
| Aubry (2015) | Housing First for People With Severe Mental Illness Who Are Homeless: A Review of the Research and Findings From the At Home-Chez soi Demonstration Project | 2015 |
| Auger (2015) | Pediatric Asthma Readmission: Asthma Knowledge Is Not Enough? | 2015 |
| Beckman (2019) | Medicare Annual Wellness Visit Association With Healthcare Quality and Costs. | 2019 |
| Bekelis (2017) | Comparison of clipping and coiling in elderly patients with unruptured cerebral aneurysms. | 2017 |
| Bell (2014) | Associations of PM2.5 constituents and sources with hospital admissions: analysis of four counties in Connecticut and Massachusetts (USA) for persons >= 65 years of age. | 2014 |
| Bell (2015) | Effect of a pharmacist counseling intervention on healthcare utilization after hospital discharge: A randomized controlled trial | 2015 |
| Benenson (2017) | Factors influencing utilization of hospital services by adult sickle cell disease patients: a systematic review. | 2017 |
| Berger (2020) | Ambulatory Care–Sensitive Conditions Associated With Potentially Avoidable Hospital Admissions. | 2020 |
| Berkman (2011) | Health literacy interventions and outcomes: an updated systematic review. | 2011 |
| Borza (2017) | Association between hospital accountable care organization status and readmission following cystectomy and other major surgery | 2017 |
| Bracken (2003) | The impact of prenatal and postnatal home visiting on utilization of care. | 2003 |
| Callahan (2012) | Transitions in Care for Older Adults with and without Dementia | 2012 |
| Chambers (2016) | Reducing 30-day Readmission After Joint Replacement. | 2016 |
| Chan (2021) | Community-based interventions for childhood asthma using comprehensive approaches: a systematic review and meta-analysis | 2021 |
| Chen (2021) | Reducing asthma hospitalisations in at-risk children: A systematic review | 2021 |
| Chopra (2017) | Non-adherence to statins and antihypertensive medications and hospitalizations among elderly fee-for-service medicare beneficiaries with pre-existing coronary artery disease and incident cancer | 2017 |
| Conway (2006) | Maternal health: does prenatal care make a difference? | 2006 |
| Crowley (2020) | Intervention protocol: OPtimising thERapy to prevent avoidable hospital Admission in the Multi-morbid elderly (OPERAM): a structured medication review with support of a computerised decision support system | 2020 |
| Cusimano (2012) | Socioeconomic status and hospital admission of team sport-related brain injuries in Canadian youth | 2012 |
| Davis (2016) | Coordinating complex care-a high-risk residency clinic | 2016 |
| DeWalt (2009) | Comparison of a one-time educational intervention to a teach-to-goal educational intervention for self-management of heart failure: design of a randomized controlled trial | 2009 |
| Estrada (2017) | Drug-safety program impact in hospitalization persistent severe asthma patients | 2017 |
| Fann (2011) | Maximizing Health Benefits and Minimizing Inequality: Incorporating Local-Scale Data in the Design and Evaluation of Air Quality Policies | 2011 |
| Fick (2002) | Delirium superimposed on dementia: a systematic review. | 2002 |
| Field (2015) | Association of early post-discharge follow-up by a primary care physician and 30-day rehospitalization among older adults | 2015 |
| Fleishmann (1995) | Longitudinal patterns of medical service use and costs among people with AIDS | 1995 |
| Fleming (2017) | Educational and Health Outcomes of Children Treated for Attention-Deficit/Hyperactivity Disorder. | 2017 |
| Fleming (2019) | Educational and health outcomes of children treated for asthma: Scotland-wide record linkage study of 683716 children | 2019 |
| Flinn (2013) | Medical home for persons with disabilities: A target for the triple aim | 2013 |
| Folger (2017) | Evaluation of Early Childhood Home Visiting to Prevent Medically Attended Unintentional Injury | 2017 |
| Follath (2006) | Beta-blockade today: the gap between evidence and practice | 2006 |
| Fonarow (2004) | Organized program to initiate lifesaving treatment in hospitalized patients with heart failure (OPTIMIZE-HF): Rationale and design | 2004 |
| Fond (2013) | Self-reported major depressive symptoms at baseline impact abstinence prognosis in smoking cessation program. A one-year prospective study | 2013 |
| Foraker (2008) | Neighborhood income, health insurance, and prehospital delay for myocardial infarction: the atherosclerosis risk in communities study. | 2008 |
| Ford (2015) | Top ten risk factors for morbidity and mortality in patients with chronic systolic heart failure and elevated heart rate: The SHIFT Risk Model. | 2015 |
| Foster (2018) | Molecular Characterization of Staphylococcus aureus Isolates From Children With Periorbital or Orbital Cellulitis. | 2018 |
| Fox (2013) | Increased perioperative b-type natriuretic peptide associates with heart failure hospitalization or heart failure death after coronary artery bypass graft surgery | 2013 |
| France (2001) | Smoking cessation interventions among hospitalized patients: what have we learned?. | 2001 |
| Francis (2004) | Acute heart failure: patient management of a growing epidemic. | 2004 |
| Franklin (2020) | A modelling-based economic evaluation of primary-care-based fall-risk screening followed by fall-prevention intervention: a cohort-based Markov model stratified by older age groups. | 2020 |
| Freedman (2013) | Treatment of acute gastroenteritis in children: an overview of systematic reviews of interventions commonly used in developed countries. | 2013 |
| Freeman (2012) | Do sales of pseudoephedrine predict methamphetamine-related hospitalizations? | 2012 |
| French (2006) | National Veterans Health Administration hospitalizations for syncope compared to acute myocardial infarction, fracture, or pneumonia in community-dwelling elders: outpatient medication and comorbidity profiles. | 2006 |
| Frencher (2010) | A comparative analysis of serious injury and illness among homeless and housed low income residents of New York City. | 2010 |
| Friedman (2018) | Association Between Left Atrial Appendage Occlusion and Readmission for Thromboembolism Among Patients With Atrial Fibrillation Undergoing Concomitant Cardiac Surgery. | 2018 |
| Froman (2005) | Randomized study of stability and change in patients' advance directives. | 2005 |
| Fu (2018) | Mean medical costs associated with vaginal and vulvar cancers for commercially insured patients in the United States and Texas. | 2018 |
| Furre (2014) | Characteristics of adolescents subjected to restraint in acute psychiatric units in Norway: a case-control study. | 2014 |
| Gaalema (2019) | The effect of executive function on adherence with a cardiac secondary prevention program and its interaction with an incentive-based intervention | 2019 |
| Galan (2015) | Assessing the effects of the Spanish partial smoking ban on cardiovascular and respiratory diseases: methodological issues. | 2015 |
| Galbraith (2011) | Cost Analysis of a Falls-prevention Program in an Orthopaedic Setting | 2011 |
| Galbraith (2017) | Long-Term Impact of a Postdischarge Community Health Worker Intervention on Health Care Costs in a Safety-Net System. | 2017 |
| Gali (2019) | Anemia profile in heart failure: High prevalence of iron deficiencyand scope for correction. a performance improvement project | 2019 |
| Gallagher (2011) | Social support and self-care in heart failure. | 2011 |
| Galletly (2011) | Bed accessibility in a private psychiatric hospital. | 2011 |
| Galloway (2016) | Hospital Readmission Following Discharge From Inpatient Rehabilitation for Older Adults With Debility. | 2016 |
| Galvin (2017) | Adverse outcomes in older adults attending emergency departments: a systematic review and meta-analysis of the Identification of Seniors At Risk (ISAR) screening tool. | 2017 |
| Galyean (2009) | Previous cesarean section and the risk of postpartum maternal complications and adverse neonatal outcomes in future pregnancies. | 2009 |
| Gambassi (2000) | Management of heart failure among very old persons living in long-term care: Has the voice of trials spread? | 2000 |
| Gamble (2010) | Admission hypoglycemia and increased mortality in patients hospitalized with pneumonia. | 2010 |
| Gan (2006) | The timed up and go test does not predict length of stay on an acute geriatric ward | 2006 |
| Ganapathi (2014) | Frailty and risk in proximal aortic surgery. | 2014 |
| Gandhi (2014) | Emergency department visit classification using the NYU algorithm. | 2014 |
| Gant (2019) | Retrospective evaluation of factors influencing transfusion requirements and outcome in cats with pelvic injury (2009-2014): 122 cases | 2019 |
| Gao (2011) | Evaluation of Dronedarone Use in the US Patient Population Between 2009 and 2010: A Descriptive Study Using a Claims Database | 2011 |
| Gao (2016) | Multifaceted interventions reduce 30-day hospital readmissions in at-risk medicaid members from four states and the district of Columbia | 2016 |
| Gao (2020) | Predictive Value of the Acute-to-Chronic Glycemic Ratio for In-Hospital Outcomes in Patients With ST-Segment Elevation Myocardial Infarction Undergoing Percutaneous Coronary Intervention. | 2020 |
| Gardiner (2019) | Acute circulatory complications in people with diabetes mellitus type 2: How admission varies between urban and rural Victoria. | 2019 |
| Gardner (2002) | Improvement in the undertreatment of osteoporosis following hip fracture | 2002 |
| Gardner (2009) | Clinical signs of infection in diabetic foot ulcers with high microbial load | 2009 |
| Gaskin (2000) | Racial and ethnic differences in preventable hospitalizations across 10 states. | 2000 |
| Gates (2021) | Health inequities related to vaccination: An evidence map of potentially influential factors and systematic review of interventions | 2021 |
| Gay (2019) | Association of Extending Hospital Length of Stay With Reduced Pediatric Hospital Readmissions. | 2019 |
| Geelhoed (2012) | Emergency department overcrowding, mortality and the 4-hour rule in Western Australia. | 2012 |
| Gerald (2010) | Cost-effectiveness of school-based asthma screening in an urban setting | 2010 |
| Gerding (2018) | Bezlotoxumab for Prevention of Recurrent Clostridium difficile Infection in Patients at Increased Risk for Recurrence. | 2018 |
| Gerhardsson (2015) | Asthma and Chronic Obstructive Pulmonary Disease Overlap Syndrome: Doubled Costs Compared with Patients with Asthma Alone. | 2015 |
| Gerke (2014) | Association of hospitalizations for asthma with seasonal and pandemic influenza. | 2014 |
| Ghaderi (2019) | Hospitalization following influenza infection and pandemic vaccination in multiple sclerosis patients: a nationwide population-based registry study from Norway | 2019 |
| Ghanayem (2012) | Interstage mortality after the Norwood procedure: Results of the multicenter Single Ventricle Reconstruction trial | 2012 |
| Gheorghiade (2005) | Rationale and design of the multicenter, randomized, double-blind, placebo-controlled study to evaluate the Efficacy of Vasopressin antagonism in Heart Failure: outcome Study with Tolvaptan (EVEREST) | 2005 |
| Gianfrancesco (2007) | Hospitalization risks in the treatment of bipolar disorder: comparison of antipsychotic medications. | 2007 |
| Gibson (2013) | A systematic review of evidence on the association between hospitalisation for chronic disease related ambulatory care sensitive conditions and primary health care resourcing. | 2013 |
| Giday (2019) | Factors other than medical acuity that influence hospitalisation: A scoping review protocol | 2019 |
| Gidwani (2015) | Association Between Acute Medical Exacerbations and Consuming or Producing Web-Based Health Information: Analysis From Pew Survey Data. | 2015 |
| Gijsberts (2017) | Effect of Monocyte-to-Lymphocyte Ratio on Heart Failure Characteristics and Hospitalizations in a Coronary Angiography Cohort. | 2017 |
| Gill (2006) | Bathing disability and the risk of long-term admission to a nursing home. | 2006 |
| Gillard (2015) | Denial of risk: The effects of positive impression management on risk assessments for psychopathic and nonpsychopathic offenders | 2015 |
| Gillis (1997) | Factors associated with unplanned discharge from psychiatric day treatment programs - A multicenter study | 1997 |
| Gilotra (2015) | Reasons for heart failure hospitalization: Nonadherence versus worsening heart failure | 2015 |
| Gilstrap (2019) | Association Between Clinical Practice Group Adherence to Quality Measures and Adverse Outcomes Among Adult Patients With Diabetes. | 2019 |
| Givertz (2014) | Renal Function Trajectories and Clinical Outcomes in Acute Heart Failure | 2014 |
| Glasziou (2002) | Cholesterol-lowering therapy with pravastatin in patients with average cholesterol levels and established ischaemic heart disease: is it cost-effective?. | 2002 |
| Gleason (2017) | Effect of drinking water source on associations between gastrointestinal illness and heavy rainfall in New Jersey. | 2017 |
| Gleason (2020) | Health navigators are an effective strategy to bridge the gap for school-aged children at risk for asthma disparities | 2020 |
| Go (2006) | Hemoglobin level, chronic kidney disease, and the risks of death and hospitalization in adults with chronic heart failure - The anemia in chronic heart failure: Outcomes and Resource Utilization (ANCHOR) Study | 2006 |
| Goeree (2013) | Economic appraisal of a community-wide cardiovascular health awareness program | 2013 |
| Goff (2014) | Project Buena Salud: Evaluation of an enhanced primary care program for low-income hispanic patients with type 2 diabetes | 2014 |
| Goforth (2015) | Exertional Heat Stroke in Navy and Marine Personnel: A Hot Topic | 2015 |
| González (2022) | Colorectal Cancer Screening in Castilla La Mancha, Spain: The Influence of Social, Economic, Demographic and Geographic Factors. | 2022 |
| Greene (2018) | Outpatient use of intravenous diuretics for treatment of heart failure: Temporal trends and associations with 30-day mortality and readmission following hospitalization for heart failure | 2018 |
| Griffin (2000) | Nonsteroidal antiinflammatory drugs and acute renal failure in elderly persons | 2000 |
| Hilts (2021) | Hospital Partnerships for Population Health: A Systematic Review of the Literature | 2021 |
| Hungerford (2018) | Influenza-associated hospitalisation, vaccine uptake and socioeconomic deprivation in an English city region: an ecological study | 2018 |
| Ikram-Bashir (2012) | Designing a behavioural-educational intervention using intervention mapping to reduce the high rates of paediatric asthma hospital admissions in an inner-city area of birmingham | 2012 |
| Jiang (2021) | Impact of High Deductible Health Plans on Diabetes Care Quality and Outcomes: Systematic Review | 2021 |
| Kaneko (2019) | Associations of Patient Experience in Primary Care With Hospitalizations and Emergency Department Visits on Isolated Islands: A Prospective Cohort Study. | 2019 |
| Kessing (2011) | Investigating a TELEmedicine solution to improve MEDication adherence in chronic Heart Failure (TELEMED-HF): study protocol for a randomized controlled trial | 2011 |
| Kiil (2014) | How does copayment for health care services affect demand, health and redistribution? A systematic review of the empirical evidence from 1990 to 2011. | 2014 |
| LeBreton (2015) | Implementation of a validated health literacy tool with teach-back education in a super utilizer patient population. | 2015 |
| Li (2017) | An APN-led transitional care program to reduce 30-day readmissions in patients with heart failure | 2017 |
| Madden (2022) | Early implementation of the structured medication review in England: a qualitative study | 2022 |
| McWilliams (2007) | Use of health services by previously uninsured Medicare beneficiaries. | 2007 |
| Cantor (2006) | Medicaid Utilization and Spending among Homeless Adults in New Jersey: Implications for Medicaid-Funded Tenancy Support Services | 2006 |
| Metcalfe (2018) | Impact of public release of performance data on the behaviour of healthcare consumers and providers | 2018 |
| Miaozhen (2017) | Using an APN-Led Transitional Care Program to Reduce 30-Day Hospital Readmissions. | 2017 |
| Moledina (2021) | A comprehensive review of prioritised interventions to improve the health and wellbeing of persons with lived experience of homelessness | 2021 |
| Nelson (2016) | Peer support for achieving independence in diabetes (peeraid): Results of a randomized controlled trial of community health worker assisted self-management support among low-income adults with diabetes | 2016 |
| Obiagwu (2018) | Developing a targeted approach to 30-day chf readmissions | 2018 |
| Pare (2007) | Systematic review of home telemonitoring for chronic diseases: the evidence base. | 2007 |
| Yinan (2020) | Permanent Supportive Housing With Housing First to Reduce Homelessness and Promote Health Among Homeless Populations With Disability: A Community Guide Systematic Review. | 2020 |
| Reategui-Sokolova (2018) | A comprehensive care program reduces the number and length of hospitalizations in systemic lupus erythematosus patients | 2018 |
| Rice (2023) | Development and Implementation of a Maryland State Program Providing Hospital Payment Incentives for Reduction in Readmission Disparities. | 2023 |
| Riggs (2009) | The influence of home care nursing visit pattern on heart failure patient outcomes. | 2009 |
| Rogstad (2022) | Social Risk Adjustment In The Hospital Readmissions Reduction Program: A Systematic Review And Implications For Policy. | 2022 |
| Rust (2013) | Inhaled corticosteroid adherence and emergency department utilization among Medicaid-enrolled children with asthma | 2013 |
| Salkever (1999) | Assertive community treatment for people with severe mental illness: The effect on hospital use and costs | 1999 |
| Smith (2023) | Racialized economic segregation and potentially preventable hospitalizations among Medicaid/CHIP-enrolled children. | 2023 |
| Tyris (2021) | Social Risk Interventions and Health Care Utilization for Pediatric Asthma A Systematic Review and Meta-analysis | 2021 |
| Soones (2016) | The mobile acute care team: Preliminary outcomes of a bundled-payment hospital at home program | 2016 |
| Stockings (2018) | Whole‐of‐community interventions to reduce population‐level harms arising from alcohol and other drug use: a systematic review and meta‐analysis. | 2018 |
| Tabit (2017) | A bundled intervention including early consultation with a cardiologist in the emergency department to reduce re-hospitalizations and healthcare cost for high-risk urban patients with acute decompensated heart failure | 2017 |
| Telfar (2020) | Renting Poorer Housing: Ecological Relationships Between Tenure, Dwelling Condition, and Income and Housing-Sensitive Hospitalizations in a Developed Country. | 2020 |
| O'Neill (2022) | The Asthma Linking Project: community-based education and support for asthma in a culturally and linguistically diverse population. | 2022 |
| Thomas (2014) | Pharmacist-led interventions to reduce unplanned admissions for older people: a systematic review and meta-analysis of randomised controlled trials. | 2014 |
| Trachtenberg (2014) | Inequities in ambulatory care and the relationship between socioeconomic status and respiratory hospitalizations: a population-based study of a canadian city. | 2014 |
| Wang (2022) | Study on the Application of the Concept of Childlike Interest with Refined Nursing Intervention in the Treatment of Children with Severe Pneumonia. | 2022 |
| Xu (2023) | An Unsupervised Machine Learning Approach to Evaluating the Association of Symptom Clusters With Adverse Outcomes Among Older Adults With Advanced Cancer: A Secondary Analysis of a Randomized Clinical Trial. | 2023 |
| Youn (2022) | Disparities in diabetes-related avoidable hospitalization among diabetes patients with disability using a nationwide cohort study. | 2022 |

Exclude on outcome: n=231

| Balamurugan (2006) | Barriers to diabetes self-management education programs in underserved rural Arkansas: implications for program evaluation. | 2006 |
| --- | --- | --- |
| Bauer (2021) | Partnering with parents to remove barriers and improve influenza immunization rates for young children | 2021 |
| Berge (2022) | Gender specific early treatment for women with alcohol addiction (EWA): Impact on work related outcomes. A 25-year registry follow-up of a randomized controlled trial (RCT). | 2022 |
| Bhaumik (2019) | Community asthma initiative: Cost analyses using claims data from a Medicaid managed care organization | 2019 |
| Bindman (2001) | Primary and secondary care for mental illness: impact of a link worker service on admission rates and costs. | 2001 |
| Boylen (2020) | Impact of professional interpreters on outcomes for hospitalized children from migrant and refugee families with limited English proficiency: a systematic review. | 2020 |
| Bozzola (2020) | Hospitalization for acute cerebellitis in children affected by varicella: how much does it cost? | 2020 |
| Brogly (2018) | Neonatal Outcomes in a Medicaid Population With Opioid Dependence. | 2018 |
| Burley (2016) | Connecting Patients to Prescription Assistance Programs: Effects on Emergency Department and Hospital Utilization. | 2016 |
| Burton (2018) | Statin Use is Not Associated with Future Long-Term Care Admission: Extended Follow-Up of Two Randomised Controlled Trials. | 2018 |
| Davis (2022) | A Randomized Controlled Trial Evaluating the Effectiveness of Supported Employment Integrated in Primary Care. | 2022 |
| Desai (2016) | Association Between Hospital Penalty Status Under the Hospital Readmission Reduction Program and Readmission Rates for Target and Nontarget Conditions | 2016 |
| DeWalt (2004) | Development and pilot testing of a disease management program for low literacy patients with heart failure | 2004 |
| DeWalt (2006) | A heart failure self-management program for patients of all literacy levels: a randomized, controlled trial [ISRCTN11535170]. | 2006 |
| Fahey (2015) | Charitable pharmacy services: Impact on patient-reported hospital use, medication access, and health status. | 2015 |
| Forchuk (2008) | Developing and testing an intervention to prevent homelessness among individuals discharged from psychiatric wards to shelters and 'No Fixed Address' | 2008 |
| Fors (2016) | Effectiveness of person-centred care after acute coronary syndrome in relation to educational level: Subgroup analysis of a two-armed randomised controlled trial | 2016 |
| Frank (2006) | Heat or eat: The low income home energy assistance program and nutritional and health risks among children less than 3 years of age | 2006 |
| Fraser (2000) | Comparison of midwifery care to medical care in hospitals in the Quebec pilot projects study: clinical indicators. L'Equipe dEvaluation des Projets-Pilotes Sages-Femmes. | 2000 |
| Fretwell (1990) | The Senior Care Study. A controlled trial of a consultative/unit-based geriatric assessment program in acute care | 1990 |
| Fuji (2024) | Effect of a Financial Education and Coaching Program for Low-Income, Single Mother Households on Child Health Outcomes | 2024 |
| Gaskin (2018) | Racial and Ethnic Composition of Hospitals' Service Areas and the Likelihood of Being Penalized for Excess Readmissions by the Medicare Program | 2018 |
| Gate (2016) | Promoting lifestyle behaviour change and well-being in hospital patients: a pilot study of an evidence-based psychological intervention. | 2016 |
| Gattis (2004) | Predischarge initiation of carvedilol in patients hospitalized for decompensated heart failure | 2004 |
| Gelkopf (2012) | Nonmedication smoking reduction program for inpatients with chronic schizophrenia: a randomized control design study. | 2012 |
| Ghahramanlou-Holloway (2012) | Post-Admission Cognitive Therapy: A Brief Intervention for Psychiatric Inpatients Admitted After a Suicide Attempt | 2012 |
| Helge (2014) | Street football is a feasible health-enhancing activity for homeless men: Biochemical bone marker profile and balance improved | 2014 |
| Hellström (2023) | Predictors of Return to Work for People with Anxiety or Depression Participating in a Randomized Trial Investigating the Effect of a Supported Employment Intervention. | 2023 |
| Huckfeldt (2019) | Thirty-Day Postdischarge Mortality Among Black and White Patients 65 Years and Older in the Medicare Hospital Readmissions Reduction Program. | 2019 |
| Johnston (2021) | Association of Race and Ethnicity and Medicare Program Type With Ambulatory Care Access and Quality Measures | 2021 |
| Justvig (2022) | The Role of Social Determinants of Health in the Use of Telemedicine for Asthma in Children | 2022 |
| Kopanitsa (2023) | A systematic scoping review of primary health care service outreach for homeless populations. | 2023 |
| Higgins (2022) | Leveraging the cigarette purchase task to understand relationships between cumulative vulnerabilities, the relative reinforcing effects of smoking, and response to reduced nicotine content cigarettes. | 2022 |
| Hoffmann (2014) | Long-Term Effectiveness of Supported Employment: 5-Year Follow-Up of a Randomized Controlled Trial | 2014 |
| Lyhne (2022) | Interventions to Prevent Potentially Avoidable Hospitalizations: A Mixed Methods Systematic Review | 2022 |
| Mallick (2022) | The impact of co‐location employment partnerships within the Australian mental health service and policy context: A systematic review. | 2022 |
| Manickas-Hill (2019) | A Review of Bundled Payments in Total Joint Replacement | 2019 |
| Moffatt (2023) | Impact of a social prescribing intervention in North East England on adults with type 2 diabetes: the SPRING_NE multimethod study. | 2023 |
| Morphew (2013) | Mobile health care operations and return on investment in predominantly underserved children with asthma: the breathmobile program. | 2013 |
| Swann (2021) | Return on investment of self-management education and home visits for children with asthma | 2021 |
| Szanton (2021) | CAPABLE program improves disability in multiple randomized trials | 2021 |
| Walker (2014) | Pregnancy, prison and perinatal outcomes in New South Wales, Australia: a retrospective cohort study using linked health data. | 2014 |
| Wildman (2023) | Impact of a link worker social prescribing intervention on non-elective admitted patient care costs: A quasi-experimental study. | 2023 |
| Fenick (2020) | A Randomized Controlled Trial of Group Well-Child Care: Improved Attendance and Vaccination Timeliness | 2020 |
| Abbott (2003) | Hospitalized psychoses after renal transplantation in the United States: incidence, risk factors, and prognosis. | 2003 |
| Abel (2018) | Reducing emergency hospital admissions: a population health complex intervention of an enhanced model of primary care and compassionate communities. | 2018 |
| Adamson (2016) | Pulmonary Artery Pressure-Guided Heart Failure Management Reduces 30-Day Readmissions. | 2016 |
| Alesiani (2014) | Systems Training for Emotional Predictability and Problem Solving (STEPPS): Program efficacy and personality features as predictors of drop-out - An Italian study | 2014 |
| Bates (2014) | Applying STAAR Interventions in Incremental Bundles: Improving Post-CABG Surgical Patient Care. | 2014 |
| Auerbach (2002) | Implementation of a voluntary hospitalist service at a community teaching hospital: improved clinical efficiency and patient outcomes. | 2002 |
| Axon (2016) | Dual health care system use is associated with higher rates of hospitalization and hospital readmission among veterans with heart failure. | 2016 |
| Banerjee (2021) | Association between Medicare's Hospital Readmission Reduction Program and readmission rates across hospitals by medicare bed share. | 2021 |
| Barath (2020) | Accountable Care Organizations and Preventable Hospitalizations Among Patients With Depression. | 2020 |
| Benjamin-Chung (2020) | Evaluation of a city-wide school-located influenza vaccination program in Oakland, California, with respect to vaccination coverage, school absences, and laboratory-confirmed influenza: A matched cohort study | 2020 |
| Benjenk (2020) | Evidence of the Linkage between Hospital-based Care Coordination Strategies and Hospital Overall (Star) Ratings | 2020 |
| Benthien (2022) | Proactive Health Support: a randomised controlled trial of telephone-based self-management support for persons at risk of hospital admission | 2022 |
| Benthien (2023) | Who benefits from self-management support? Results from a randomized controlled trial | 2023 |
| Bhandari (2023) | Interventions to Reduce Hospital Readmissions in Older African Americans: A Systematic Review of Studies Including African American Patients. | 2023 |
| Birman-Deych (2006) | Use and effectiveness of warfarin in medicare beneficiaries with atrial fibrillation | 2006 |
| Boege (2014) | Effectiveness of hometreatment in light of clinical elements, chances, and limitations | 2014 |
| Borup (2019) | Healthcare use before and after changing disability pension policy: a regional Danish cohort study. | 2019 |
| Townshend (2022) | BReATHE interventions (Beating Regional Asthma Through Health Education)-an innovative approach to children's asthma care in the North East and North Cumbria, UK: an interventional study | 2022 |
| Brooker (2007) | Admission decisions following contact with an emergency mental health assessment and intervention service. | 2007 |
| Butz (2010) | Influence of Caregiver and Provider Communication on Symptom Days and Medication Use for Inner-City Children With Asthma | 2010 |
| Capomolla (2004) | Heart failure case disease management program: a pilot study of home telemonitoring versus usual care | 2004 |
| Catov (2005) | Asthma home teaching: two evaluation approaches | 2005 |
| Chaiyachati (2018) | Changes to Racial Disparities in Readmission Rates After Medicare's Hospital Readmissions Reduction Program Within Safety-Net and Non-Safety-Net Hospitals. | 2018 |
| Chambers (2023) | Reducing unplanned hospital admissions from care homes: a systematic review. | 2023 |
| Charles (2020) | Improving transitions from acute care to home among complex older adults using the LACE Index and care coordination. | 2020 |
| Chartrand (2023) | Patient- and family-centred care transition interventions for adults: a systematic review and meta-analysis of RCTs. | 2023 |
| Chen (2019) | Hospital Readmissions Reduction Program: Intended and Unintended Effects | 2019 |
| Chirila (2017) | Health care resource use analysis of paliperidone palmitate 3 month injection from two phase 3 clinical trials | 2017 |
| Chovanec (2021) | Association of Discharge Disposition with Outcomes. | 2021 |
| Clar (2003) | Routine hospital admission versus out-patient or home care in children at diagnosis of type 1 diabetes mellitus | 2003 |
| Cleland (2005) | Noninvasive home telemonitoring for patients with heart failure at high risk of recurrent admission and death: the Trans-European Network-Home-Care Management System (TEN-HMS) study | 2005 |
| Clement (2018) | Urban-Rural Differences in Skilled Nursing Facility Rehospitalization Rates. | 2018 |
| Coombes (2023) | Discharge interventions for First Nations people with a chronic condition or injury: a systematic review | 2023 |
| Dai (2022) | Family Practices in Transforming Clinical Practice Initiative Showed No Changes in Medicare Costs or Utilization | 2022 |
| Dale (2003) | An evaluation of the west Surrey telemedicine monitoring project | 2003 |
| Davis (2015) | Innovative Care Models for High-Cost Medicare Beneficiaries: Delivery System and Payment Reform to Accelerate Adoption | 2015 |
| de Oliveira (2023) | Psychoeducational Intervention for Reducing Heart Failure Patients' Rehospitalizations and Promoting Their Quality of Life and Posttraumatic Growth at the 1-Year Follow-Up: A Randomized Clinical Trial. | 2023 |
| Deek (2016) | Family-centred approaches to healthcare interventions in chronic diseases in adults: a quantitative systematic review | 2016 |
| Rotenstein (2022) | Development of a Primary Care Transitions Clinic in an Academic Medical Center | 2022 |
| Doherty (2022) | Minding the gap-an examination of a pharmacist case management medicines optimisation intervention for older people in intermediate care settings | 2022 |
| Dols (2020) | Relationship of Nurse-Led Education Interventions to Liver Transplant Early Readmission. | 2020 |
| Duminy (2022) | Complex community health and social care interventions - Which features lead to reductions in hospitalizations for ambulatory care sensitive conditions? A systematic literature review. | 2022 |
| Dummit (2016) | Association Between Hospital Participation in a Medicare Bundled Payment Initiative and Payments and Quality Outcomes for Lower Extremity Joint Replacement Episodes. | 2016 |
| Dunleavy (2019) | Medical, Mental Health, and Social Service Linkage Predicts Better HIV Outcomes: A Network Analytic Approach. | 2019 |
| Dunn (2021) | The impact of community nurse-led interventions on the need for hospital use among older adults: An integrative review. | 2021 |
| Dye (2018) | Improving Chronic Disease Self-Management by Older Home Health Patients through Community Health Coaching. | 2018 |
| Edwards (2017) | Preventing Hospitalization with Veterans Affairs Home-Based Primary Care: Which Individuals Benefit Most? | 2017 |
| Eisenstein (2002) | Vasopeptidase inhibitor reduces inhospital costs for patients with congestive heart failure: results from the IMPRESS trial. Inhibition of Metallo Protease by BMS-186716 in a Randomized Exercise and Symptoms Study in Subjects With Heart Failure | 2002 |
| Eisenstein (2009) | Long-term clinical and economic analysis of the Endeavor drug-eluting stent versus the Driver bare-metal stent: 4-year results from the ENDEAVOR II trial (Randomized Controlled Trial to Evaluate the Safety and Efficacy of the Medtronic AVE ABT-578 Eluting Driver Coronary Stent in De Novo Native Coronary Artery Lesions). | 2009 |
| El-Gamil (2017) | What is the best setting for receiving dialysis vascular access repair and maintenance services?. | 2017 |
| Ell (2010) | Collaborative Depression Treatment in Older and Younger Adults With Physical Illness: Pooled Comparative Analysis of Three Randomized Clinical Trials | 2010 |
| Epstein (2001) | The role of public clinics in preventable hospitalizations among vulnerable populations. | 2001 |
| Esslinger (2014) | Exploratory analysis of the relationship between home health agency engagement in a national campaign and reduction in acute care hospitalization in US home care patients | 2014 |
| Wilcox (2018) | Evaluation of a Hospital: Community Partnership to Reduce 30-Day Readmissions. | 2018 |
| Finlayson (2018) | Transitional care interventions reduce unplanned hospital readmissions in high-risk older adults | 2018 |
| Fish-Trotter (2018) | Design and rationale of a randomized trial: Using short stay units instead of routine admission to improve patient centered health outcomes for acute heart failure patients (SSU-AHF). | 2018 |
| Fitchett (2018) | Effects of empagliflozin on risk for cardiovascular death and heart failure hospitalization across the spectrum of heart failure risk in the EMPA-REG OUTCOME® trial | 2018 |
| Fitzpatrick (2021) | Palivizumab's real-world effectiveness: a population-based study in Ontario, Canada, 1993-2017 | 2021 |
| Fleming (1997) | Brief physician advice for problem alcohol drinkers - A randomized controlled trial in community-based primary care practices | 1997 |
| Fleming (2018) | Early Ambulation Among Hospitalized Heart Failure Patients Is Associated With Reduced Length of Stay and 30-Day Readmissions | 2018 |
| Forbes (2006) | Evaluation of a MS specialist nurse programme. | 2006 |
| Fornaro (2022) | Homelessness and health-related outcomes: an umbrella review of observational studies and randomized controlled trials. | 2022 |
| Froelicher (2004) | Women's Initiative for Nonsmoking-VII: evaluation of health service utilization and costs among women smokers with cardiovascular disease. | 2004 |
| Fry (2018) | Comparison of Risk-Adjusted Outcomes in Medicare Open versus Laparoscopic Cholecystectomy. | 2018 |
| Fung (2013) | Adverse clinical events among medicare beneficiaries using antipsychotic drugs: linking health insurance benefits and clinical needs. | 2013 |
| Gaalema (2019) | Financial Incentives to Increase Cardiac Rehabilitation Participation Among Low-Socioeconomic Status Patients A Randomized Clinical Trial | 2019 |
| Gaillard (2022) | Does integrated care mean fewer hospitalizations? An evaluation of a French field experiment | 2022 |
| Ganapathy (2017) | Continuing care with nebulized bronchodilators after hospital discharge and impact on readmissions: analysis of medicare COPD beneficiaries receiving arformoterol vs nebulized short-acting agents | 2017 |
| George (1999) | A comprehensive educational program improves clinical outcome measures in inner-city patients with asthma | 1999 |
| Ghani (2016) | Needle disinfectant technique during prostate biopsy is associated with less infection-related hospitalization: Results from a surgical collaborative | 2016 |
| Gheorghiade (2013) | Effect of oral digoxin in high-risk heart failure patients: a pre-specified subgroup analysis of the DIG trial. | 2013 |
| Gilman (2014) | Effectiveness of a post-emergency department automated telephone call on follow-up appointment compliance and association of compliance with subsequent hospitalization | 2014 |
| Goff (2017) | Effects of an Enhanced Primary Care Program on Diabetes Outcomes. | 2017 |
| Golberstein (2015) | Effect of the Affordable Care Act's Young Adult Insurance Expansions on Hospital-Based Mental Health Care | 2015 |
| Gomis-Pastor (2023) | Does an eHealth Intervention Reduce Complications and Healthcare Resources? A mHeart Single-Center Randomized-Controlled Trial | 2023 |
| Gorostiza (2021) | Dynamic evaluation of the comparative effectiveness of an integrated program for heart failure care | 2021 |
| Gruneir (2007) | Hospitalization of nursing home residents with cognitive impairments: the influence of organizational features and state policies. | 2007 |
| Guo (2001) | Assessing the impact of community-based mobile crisis services on preventing hospitalization. | 2001 |
| Gupta (2018) | The Hospital Readmissions Reduction Program: Evidence for Harm. | 2018 |
| Hahn (2014) | Fewer Hospitalizations for Chronic Obstructive Pulmonary Disease in Communities With Smoke-Free Public Policies | 2014 |
| Hallgren (2013) | The Swedish six-community alcohol and drug prevention trial: effects on youth drinking | 2013 |
| Harrop (2024) | Effects of a culturally informed model of care for Aboriginal and Torres Strait Islander patients with acute coronary syndrome in a tertiary hospital in Australia: a pre-post, quasi-experimental, interventional study. | 2024 |
| Hatfield (2016) | Survival and Toxicity After Cisplatin Plus Etoposide Versus Carboplatin Plus Etoposide for Extensive-Stage Small-Cell Lung Cancer in Elderly Patients. | 2016 |
| Havers (2016) | Case-Control Study of Vaccine Effectiveness in Preventing Laboratory-Confirmed Influenza Hospitalizations in Older Adults, United States, 2010-2011 | 2016 |
| Hollinghurst (2022) | Do home adaptation interventions help to reduce emergency fall admissions? A national longitudinal data-linkage study of 657,536 older adults living in Wales (UK) between 2010 and 2017. | 2022 |
| Horner (2016) | Enhancing Asthma Self-Management in Rural School-Aged Children: A Randomized Controlled Trial | 2016 |
| Ibrahim (2018) | Emergency Surgery for Medicare Beneficiaries Admitted to Critical Access Hospitals. | 2018 |
| Inampudi (2014) | Spironolactone use and higher hospital readmission for Medicare beneficiaries with heart failure, left ventricular ejection fraction <45%, and estimated glomerular filtration rate <45 ml/min/1.73 m(2.). | 2014 |
| Ingber (2017) | Initiative To Reduce Avoidable Hospitalizations Among Nursing Facility Residents Shows Promising Results | 2017 |
| Inohara (2018) | Association of Renin-Angiotensin Inhibitor Treatment with Mortality and Heart Failure Readmission in Patients with Transcatheter Aortic Valve Replacement | 2018 |
| Duckett (2016) | Investigating a Multistakeholder Alliance Approach to Reducing Hospital Readmissions. | 2016 |
| IsHak (2024) | Comparative Effectiveness of Psychotherapy vs Antidepressants for Depression in Heart Failure: A Randomized Clinical Trial. | 2024 |
| Jäckel (2017) | Effects of Sustained Competitive Employment on Psychiatric Hospitalizations and Quality of Life | 2017 |
| Janevic (2022) | Analysis of State Medicaid Expansion and Access to Timely Prenatal Care Among Women Who Were Immigrant vs US Born. | 2022 |
| Jennings (2019) | Health Care Utilization and Cost Outcomes of a Comprehensive Dementia Care Program for Medicare Beneficiaries | 2019 |
| Jha (2007) | Performance measures, vaccinations, and pneumonia rates among high-risk patients in Veterans Administration health care. | 2007 |
| Joo (2014) | Community-Based Case Management, Hospital Utilization, and Patient-Focused Outcomes in Medicare Beneficiaries. | 2014 |
| Kane (2017) | Effects of an Intervention to Reduce Hospitalizations From Nursing Homes: A Randomized Implementation Trial of the INTERACT Program. | 2017 |
| Khazanie (2014) | Trends in the use and outcomes of ventricular assist devices among medicare beneficiaries, 2006 through 2011. | 2014 |
| Kilburn (2017) | Home Visiting and Use of Infant Health Care: A Randomized Clinical Trial. | 2017 |
| Kinsey (2023) | Impact of interventions to improve recovery of older adults following planned hospital admission on quality-of-life following discharge: linked-evidence synthesis. | 2023 |
| Klug (2014) | North Dakota assistance program for dementia caregivers lowered utilization, produced savings, and increased empowerment. | 2014 |
| Koniak-Griffin (2002) | Public health nursing care for adolescent mothers: Impact on infant health and selected maternal outcomes at 1 year postbirth | 2002 |
| Kosiborod (2005) | Anemia and outcomes in patients with heart failure: a study from the National Heart Care Project. | 2005 |
| Kronman (2008) | Can primary care visits reduce hospital utilization among Medicare beneficiaries at the end of life?. | 2008 |
| Krska (2001) | Pharmacist-led medication review in patients over 65: a randomized, controlled trial in primary care | 2001 |
| Kuo (2013) | Association between proportion of provider clinical effort in nursing homes and potentially avoidable hospitalizations and medical costs of nursing home residents. | 2013 |
| Kuo (2015) | Potentially Preventable Hospitalizations in Medicare Patients With Diabetes: A Comparison of Primary Care Provided by Nurse Practitioners Versus Physicians. | 2015 |
| Leary (2023) | Building an inpatient addiction medicine consult service in Sudbury, Canada: preliminary data and lessons learned in the era of COVID-19 | 2023 |
| Leckcivilize (2021) | Impact of an anticipatory care planning intervention on unscheduled acute hospital care using difference-in-difference analysis. | 2021 |
| Lee (2018) | Spillover effects of the hospital readmission reduction program on radical cystectomy readmissions | 2018 |
| Li (2018) | Does Medicare Advantage Reduce Racial Disparity in 30-Day Rehospitalization for Medicare Beneficiaries?. | 2018 |
| Lichtman (2009) | Stroke Patient Outcomes in US Hospitals Before the Start of the Joint Commission Primary Stroke Center Certification Program | 2009 |
| Lindstroem (2021) | The effect of the employment of experienced physicians in the Emergency Department on quality of care and equality-a quasi-experimental retrospective cohort study | 2021 |
| Lutfiyya (2017) | Does primary care diabetes management provided to Medicare patients differ between primary care physicians and nurse practitioners? | 2017 |
| Mann (2021) | Impact of an integrated community-based model of care for older people with complex conditions on hospital emergency presentations and admissions: a step-wedged cluster randomized trial. | 2021 |
| Marafino (2021) | Evaluation of an intervention targeted with predictive analytics to prevent readmissions in an integrated health system: observational study. | 2021 |
| Masoudi (2014) | Comparative effectiveness of cardiac resynchronization therapy with an implantable cardioverter-defibrillator versus defibrillator therapy alone: a cohort study. | 2014 |
| McDermott (2001) | Improving diabetes care in the primary healthcare setting: a randomised cluster trial in remote Indigenous communities | 2001 |
| McWilliams (2013) | Changes in Health Care Spending and Quality for Medicare Beneficiaries Associated With a Commercial ACO Contract | 2013 |
| Miller (2017) | Management of Heart Failure in a Rural Community. | 2017 |
| Momesso (2023) | Effect of removing the 4-hour access standard in the ED: a retrospective observational study. | 2023 |
| Morello (2016) | A Telephone Support Program to Reduce Costs and Hospital Admissions for Patients at Risk of Readmissions: Lessons from an Evaluation of a Complex Health Intervention. | 2016 |
| Moreno (2021) | Connecting Provider to home: A home-based social intervention program for older adults | 2021 |
| Murphy (2019) | 2018 John Charnley Award: Analysis of US Hip Replacement Bundled Payments: Physician-initiated Episodes Outperform Hospital-initiated Episodes. | 2019 |
| Musich (2014) | CLINICAL. Personalized Preventive Care Reduces Healthcare Expenditures Among Medicare Advantage Beneficiaries. | 2014 |
| Nguyen (2023) | Effect of the population health inpatient Medicare Advantage pharmacist intervention on hospital readmissions: A quasi-experimental controlled study. | 2023 |
| Nguyen (2023) | Thirty-Day Unplanned Readmissions Following Elective and Acute Percutaneous Coronary Intervention | 2023 |
| North (2019) | Design, Implementation, and Assessment of a Public Comprehensive Specialty Care Program for Early Psychosis | 2019 |
| Oliva (2008) | The impact of RN case management on inpatient and ED utilization in a chronically ill, older adult, community-dwelling population. | 2008 |
| Ong (2017) | A Community-Partnered, Participatory, Cluster-Randomized Study of Depression Care Quality Improvement: Three-Year Outcomes | 2017 |
| Orzol (2018) | The Impact of a Health Information Technology-Focused Patient-centered Medical Neighborhood Program Among Medicare Beneficiaries in Primary Care Practices: The Effect on Patient Outcomes and Spending. | 2018 |
| Pandolfi (2017) | Associations between nursing home performance and hospital 30-day readmissions for acute myocardial infarction, heart failure and pneumonia at the healthcare community level in the United States. | 2017 |
| Peikes (2009) | Effects of Care Coordination on Hospitalization, Quality of Care, and Health Care Expenditures Among Medicare Beneficiaries 15 Randomized Trials | 2009 |
| Portnoy (2006) | Utilization patterns in an asthma intervention. | 2006 |
| Reddy (2020) | Association of High-Cost Health Care Utilization With Longitudinal Changes in Patient-Centered Medical Home Implementation. | 2020 |
| Riera-Molist (2023) | A Brief Psychoeducation Intervention to Prevent Rehospitalization in Severe Mental Disorder Inpatients. | 2023 |
| Riley (2015) | Program evaluation of remote heart failure monitoring: healthcare utilization analysis in a rural regional medical center. | 2015 |
| Roberts (2018) | The Value-Based Payment Modifier: Program Outcomes and Implications for Disparities. | 2018 |
| Roy (2023) | Association Between Hospital Participation in Value-Based Programs and Timely Initiation of Post-Acute Home Health Care, Functional Recovery, and Hospital Readmission After Joint Replacement. | 2023 |
| Rymer (2018) | Advanced Practice Provider Versus Physician-Only Outpatient Follow-Up After Acute Myocardial Infarction. | 2018 |
| Saleh (2012) | An effectiveness and cost-benefit analysis of a hospital-based discharge transition program for elderly Medicare recipients. | 2012 |
| Sandhu (2019) | Comparison of the change in heart failure readmission and mortality rates between hospitals subject to hospital readmission reduction program penalties and critical access hospitals. | 2019 |
| Schermerhorn (2008) | Endovascular vs. open repair of abdominal aortic aneurysms in the medicare population | 2008 |
| Schley (2008) | Early intervention with difficult to engage, 'high-risk' youth: evaluating an intensive outreach approach in youth mental health. | 2008 |
| Schmidt-Kraepelin (2009) | Prevention of rehospitalization in schizophrenia: results of an integrated care project in Germany. | 2009 |
| Schraeder (2001) | The effects of a collaborative model of primary care on the mortality and hospital use of community-dwelling older adults. | 2001 |
| Segal (2016) | Economic evaluation of Indigenous health worker management of poorly controlled type 2 diabetes in north Queensland. | 2016 |
| Shafi (2017) | Antihypertensive medications and risk of death and hospitalizations in US hemodialysis patients Evidence from a cohort study to inform hypertension treatment practices | 2017 |
| Shah (2016) | COPD Readmissions: Addressing COPD in the Era of Value-based Health Care. | 2016 |
| Shin (2013) | Effect of antihypertensive medication adherence on hospitalization for cardiovascular disease and mortality in hypertensive patients | 2013 |
| Sibille (2023) | Benzodiazepine Receptor Agonists Use and Cessation Among Multimorbid Older Adults with Polypharmacy: Secondary Analysis from the OPERAM Trial. | 2023 |
| Smith (2016) | Treatment patterns, overall survival, healthcare resource use and costs in elderly Medicare beneficiaries with chronic myeloid leukemia using second-generation tyrosine kinase inhibitors as second-line therapy | 2016 |
| Smith (2022) | Impactability Modeling for Reducing Medicare Accountable Care Organization Payments and Hospital Events in High-Need High-Cost Patients: Longitudinal Cohort Study | 2022 |
| Som (2017) | Improving Dialysis Adherence for High Risk Patients Using Automated Messaging: proof of Concept | 2017 |
| Sommer (2011) | Children's Hospital Boston Community Asthma Initiative: Partnerships and Outcomes Advance Policy Change | 2011 |
| Spangler (2023) | The Impact of the Swedish Care Coordination Act on Hospital Readmission and Length-of-Stay among Multi- Morbid Elderly Patients: A Controlled Interrupted Time Series Analysis | 2023 |
| Spitzer (2020) | A geographic analysis of racial disparities in use of pulmonary rehabilitation after hospitalization for COPD exacerbation | 2020 |
| Stas (2023) | Implications of a Reduced Length of Postpartum Hospital Stay on Maternal and Neonatal Readmissions, an Observational Study. | 2023 |
| Stergiopoulos (2015) | Effectiveness of Housing First with Intensive Case Management in an Ethnically Diverse Sample of Homeless Adults with Mental Illness: A Randomized Controlled Trial | 2015 |
| Stuart (2010) | Impact of maintenance therapy on hospitalization and expenditures for Medicare beneficiaries with chronic obstructive pulmonary disease. | 2010 |
| Betancourt (2022) | Substance Use Relapse Among Veterans at Termination of Treatment for Substance Use Disorders. | 2022 |
| Swankoski (2023) | Intensive care management for high-risk veterans in a patient-centered medical home - do some veterans benefit more than others?. | 2023 |
| Swanson (2022) | Association between primary care appointment lengths and subsequent ambulatory reassessment, emergency department care, and hospitalization: a cohort study | 2022 |
| Takaku (2016) | Effects of reduced cost-sharing on children's health: Evidence from Japan | 2016 |
| Tantipinichwong (2017) | Impact on 30-day hospital readmissions of post-discharge medication reconciliation in a medicare advantage patient population | 2017 |
| Ten (2019) | The Impact of a Heart Failure Management Program in a Medicare Advantage Population | 2019 |
| Thomas (2009) | High-dose inhaled corticosteroids versus add-on long-acting beta-agonists in asthma: an observational study. | 2009 |
| Thompson (2018) | Community Navigators Reduce Hospital Utilization in Super-Utilizers. | 2018 |
| Timbie (2017) | Implementation of Medical Homes in Federally Qualified Health Centers. | 2017 |
| Tran (2024) | The Australian Health Care Homes trial: quality of care and patient outcomes. A propensity score-matched cohort study | 2024 |
| Unoki (2024) | Exploring the influence of a financial incentive scheme on early mobilization and rehabilitation in ICU patients: an interrupted time-series analysis. | 2024 |
| Unruh (2017) | Hospitalization event notifications and reductions in readmissions of Medicare fee-for-service beneficiaries in the Bronx, New York | 2017 |
| Unruh (2018) | Hospital participation in Meaningful Use and racial disparities in readmissions. | 2018 |
| van Loon-van (2021) | Telephone follow-up to reduce unplanned hospital returns for older emergency department patients: A randomized trial. | 2021 |
| Vergara (2021) | Predictors for Telephone Outreach Post-hospital Discharge. | 2021 |
| Vohr (2017) | Impact of a Transition Home Program on Rehospitalization Rates of Preterm Infants. | 2017 |
| Wadhera (2019) | Association of State Medicaid Expansion With Quality of Care and Outcomes for Low-Income Patients Hospitalized With Acute Myocardial Infarction. | 2019 |
| Wang (2010) | Impact of Drug Cost Sharing on Service Use and Adverse Clinical Outcomes in Elderly Receiving Antidepressants | 2010 |
| Welch (2009) | Assessment of the impact of medication therapy management delivered to home-based Medicare beneficiaries | 2009 |
| Whitcomb (2019) | Association of Decision Support for Hospital Discharge Disposition With Outcomes. | 2019 |
| Wong (2018) | Effects of the VA patient centered medical home initiative on healthcare utilization: Results after four years | 2018 |
| Xu (2022) | The impact of community nursing program on healthcare utilization: A program evaluation. | 2022 |
| Xu (2022) | Does the abolition of copayment increase ambulatory care utilization?: a quasi-experimental study in Germany | 2022 |
| Zabawa (2018) | Thirty-day rehospitalizations among elderly patients with acute myocardial infarction Impact of postdischarge ambulatory care | 2018 |
| Zillich (2013) | A randomized, controlled pragmatic trial of telephonic medication therapy management to reduce hospitalization in home health patients | 2013 |
| Zillich (2014) | A Randomized, Controlled Pragmatic Trial of Telephonic Medication Therapy Management to Reduce Hospitalization in Home Health Patients | 2014 |

Exclude study period: n=4

| Ahring (1992) | Telephone modem access improves diabetes control in those with insulin-requiring diabetes | 1992 |
| --- | --- | --- |
| Gadomski (1998) | Impact of a Medicaid primary care provider and preventive care on pediatric hospitalization | 1998 |
| GAZIANO (1994) | IS IT TIME TO REASSESS THE RISK FOR THE GROWTH-RETARDED FETUS WITH NORMAL DOPPLER VELOCIMETRY OF THE UMBILICAL ARTERY | 1994 |
| Geddes (1994) | Prediction of outcome following a first episode of schizophrenia. A follow-up study of Northwick Park first episode study subjects | 1994 |

Exclude targeted intervention: n=137

| Burns (2007) | The effectiveness of supported employment for people with severe mental illness: a randomised controlled trial | 2007 |
| --- | --- | --- |
| Forget (2011) | The Town with No Poverty: The Health Effects of a Canadian Guaranteed Annual Income Field Experiment | 2011 |
| Jackson (2011) | Reduced acute hospitalisation with the healthy housing programme | 2011 |
| Rodgers (2018) | Emergency hospital admissions associated with a non-randomised housing intervention meeting national housing quality standards: a longitudinal data linkage study. | 2018 |
| de Cuba (2023) | Child Care Feeding Programs Associated With Food Security and Health for Young Children From Families With Low Incomes | 2023 |
| Pollack (2021) | Using the Moving to Opportunity Experiment to Investigate the Long-Term Impact of Neighborhoods on Healthcare Use by Specific Clinical Conditions and Type of Service | 2021 |
| Watkins (2001) | Changes in mental health and service use after termination of SSI benefits | 2001 |
| Kackin (2020) | A Study on Decreasing Asthma Attacks and Hospitalization: Discharge Training and Home Monitoring By Nurses. | 2020 |
| Gazey (2019) | The Cottage: providing medical respite care in a home-like environment for people experiencing homelessness. | 2019 |
| Goldzahl (2022) | The effects of multi-disciplinary integrated care on healthcare utilization: Evidence from a natural experiment in the UK. | 2022 |
| Malden (2023) | Reducing hospital readmissions amongst people experiencing homelessness: a mixed-methods evaluation of a multi-disciplinary hospital in-reach programme. | 2023 |
| Norris (2023) | Impact of removing prescription co-payments on the use of costly health services: a pragmatic randomised controlled trial. | 2023 |
| Ress (2024) | The impact of integrated care on health care utilization and costs in a socially deprived urban area in Germany: A difference-in-differences approach within an event-study framework. | 2024 |
| Salvalaggio (2022) | Impact of an addiction medicine consult team intervention in a Canadian inner city hospital on acute care utilization: a pragmatic quasi-experimental study. | 2022 |
| Bischoff (2013) | Advance care planning and the quality of end-of-life care in older adults. | 2013 |
| Blumenthal (2017) | Association Between Treatment by Locum Tenens Internal Medicine Physicians and 30-Day Mortality Among Hospitalized Medicare Beneficiaries. | 2017 |
| Brown (2012) | Six Features Of Medicare Coordinated Care Demonstration Programs That Cut Hospital Admissions Of High-Risk Patients | 2012 |
| Castro (2003) | Asthma intervention program prevents readmissions in high healthcare users | 2003 |
| Chan (2023) | Ambulatory Intensive Care for Medically Complex Patients at a Health Care Clinic for Individuals Experiencing Homelessness: The SUMMIT Randomized Clinical Trial. | 2023 |
| Gallo (2023) | Mi Puente (My Bridge) Care Transitions Program for Hispanic/Latino Adults with Multimorbidity: Results of a Randomized Controlled Trial. | 2023 |
| Guo (2005) | Impact of school-based health centers on children with asthma | 2005 |
| Harrison-Long (2023) | The impact of the baby friendly hospital initiative on healthcare utilization among newborns insured by Medicaid in Delaware | 2023 |
| Heisler (2022) | Impact on Health Care Utilization and Costs of a Medicaid Community Health Worker Program in Detroit, 2018-2020: A Randomized Program Evaluation. | 2022 |
| Jackson (2013) | Transitional care cut hospital readmissions for North Carolina Medicaid patients with complex chronic conditions. | 2013 |
| Lavallee (2023) | Supplemental Nutrition Assistance Program Emergency Allotments and Food Security, Hospitalizations, and Hospital Capacity. | 2023 |
| Lob (2000) | Case management: a controlled evaluation of persons with diabetes. | 2000 |
| Mainardi (2023) | Reducing asthma exacerbations in vulnerable children through a medical-legal partnership. | 2023 |
| Mattison (2023) | Effectiveness of a community-driven, asthma intervention: project asthma in-home response | 2023 |
| Msw (2023) | A descriptive study of screening and navigation on health-related social needs in a safety-net hospital emergency department. | 2023 |
| Pantell (2022) | Association of 2 Social Needs Interventions With Child Emergency Department Use and Hospitalizations A Secondary Analysis of a Randomized Clinical Trial | 2022 |
| Park-Clinton (2023) | A Targeted Discharge Planning for High-Risk Readmissions: Focus on Patients and Caregivers. | 2023 |
| Quinton (2023) | Differential Impact of a Plan-Led Standardized Complex Care Management Intervention on Subgroups of High-Cost High-Need Medicaid Patients | 2023 |
| Rabito (2017) | A single intervention for cockroach control reduces cockroach exposure and asthma morbidity in children | 2017 |
| Robinson (2008) | The impact of literacy enhancement on asthma-related outcomes among underserved children. | 2008 |
| Roth (2023) | Evaluation of an Integrated Intervention to Address Clinical Care and Social Needs Among Patients with Type 2 Diabetes. | 2023 |
| Sabbatini (2022) | Impact of a statewide Emergency Department Information Exchange on health care use and expenditures | 2022 |
| Smith (2023) | The impacts of the 340B Program on health care quality for low-income patients. | 2023 |
| Spoelstra (2022) | Results of a multi-site pragmatic hybrid type 3 cluster randomized trial comparing level of facilitation while implementing an intervention in community-dwelling disabled and older adults in a Medicaid waiver | 2022 |
| Steinman (2023) | Can a Home-Based Collaborative Care Model Reduce Health Services Utilization for Older Medicaid Beneficiaries Living with Depression and Co-occurring Chronic Conditions? A Quasi-experimental Study. | 2023 |
| Tavares (2023) | The effect of the right care, right place, right time (R3) initiative on Medicare health service use among older affordable housing residents | 2023 |
| Yang (2022) | Did the Hospital Readmissions Reduction Program Reduce Readmissions without Hurting Patient Outcomes at High Dual-Proportion Hospitals Prior to Stratification?. | 2022 |
| Zhao (2024) | Evaluating the impact of the Medicaid expansion program on diabetes hospitalization. | 2024 |
| Adesanya (2005) | Impact of a crisis assessment and treatment service on admissions into an acute psychiatric unit. | 2005 |
| Kim (2018) | The Effects of Health Coverage Schemes on Length of Stay and Preventable Hospitalization in Seoul. | 2018 |
| Lichtl (2019) | Effects of introducing a walk-in clinic on ambulatory care sensitive hospitalisations among asylum seekers in Germany: a single-centre pre-post intervention study using medical records | 2019 |
| Lopez (2006) | Randomized clinical trial of a postdischarge pharmaceutical care program vs regular follow-up in patients with heart failure. | 2006 |
| Allaire (2023) | Does access to free medication reduce health system costs? An evaluation of the Dispensary of Hope program | 2023 |
| Aparasu (2014) | Risk of hospitalization and use of first- versus second-generation antipsychotics among nursing home residents. | 2014 |
| Apter (2020) | Patient Advocates for Low-Income Adults with Moderate to Severe Asthma: A Randomized Clinical Trial | 2020 |
| Balamurugan (2006) | Diabetes self-management education program for medicaid recipients - A continuous quality improvement process | 2006 |
| Bera (2014) | Hospitalization resource utilization and costs among Medicaid insured patients with schizophrenia with different treatment durations of long-acting injectable antipsychotic therapy. | 2014 |
| Bollinger (2010) | The Breathmobile program: a good investment for underserved children with asthma | 2010 |
| Brown (2020) | Impact of the Affordable Care Act Medicaid Expansion on Access to Care and Hospitalization Charges for Lupus Patients. | 2020 |
| Carter (2021) | Effect of Community Health Workers on 30-Day Hospital Readmissions in an Accountable Care Organization Population A Randomized Clinical Trial | 2021 |
| Castellanos (2016) | Home Monitoring Program Reduces Mortality in High-Risk Sociodemographic Single-Ventricle Patients | 2016 |
| Chung (2014) | Impact of a Clinical Pharmacy Program on Changes in Hemoglobin A1c, Diabetes-Related Hospitalizations, and Diabetes-Related Emergency Department Visits for Patients with Diabetes in an Underserved Population | 2014 |
| Davidson (2003) | Evaluation of access, a primary care program for indigent patients: Inpatient and emergency room utilization | 2003 |
| Dayal (2019) | Hospital Utilization Among Rural Children Served by Pediatric Neurology Telemedicine Clinics. | 2019 |
| de la Vega (2023) | A Pharmacy Liaison-Patient Navigation Intervention to Reduce Inpatient and Emergency Department Utilization Among Primary Care Patients in a Medicaid Accountable Care Organization: A Nonrandomized Controlled Trial | 2023 |
| Dreisbach (2023) | Improving childhood asthma outcomes in East Harlem: the East Harlem Asthma Center of Excellence's Asthma Counselor Program. | 2023 |
| Duggan (2004) | Randomized trial of a statewide home visiting program: impact in preventing child abuse and neglect | 2004 |
| Duru (2020) | Evaluation of a National Care Coordination Program to Reduce Utilization Among High-cost, High-need Medicaid Beneficiaries With Diabetes | 2020 |
| Dush (2001) | Reducing psychiatric hospital use of the rural poor through intensive transitional acute care. | 2001 |
| Eakin (2012) | Asthma in Head Start children: Effects of the Breathmobile program and family communication on asthma outcomes | 2012 |
| Edge (2022) | Breaking the Cycle Care Coordination Interventions and Sickle Cell Readmissions | 2022 |
| Pantell (2020) | Effects of In-Person Navigation to Address Family Social Needs on Child Health Care Utilization: A Randomized Clinical Trial. | 2020 |
| Englander (2014) | The care transitions innovation (C-train) for socioeconomically disadvantaged adults, results of a clustered randomized controlled trial | 2014 |
| Fine (2023) | Health Care Utilization among Homeless-Experienced Adults Who Were Seen by a Mobile Addiction Health Clinic in Boston, Massachusetts: A Quasi-Experimental Study. | 2023 |
| Fritz (2022) | Association of Race and Neighborhood Disadvantage with Patient Engagement in a Home-Based COVID-19 Remote Monitoring Program | 2022 |
| Gill (2003) | Does having an outpatient visit after hospital discharge reduce the likelihood of readmission?. | 2003 |
| Goldman (2014) | Support from hospital to home for elders: a randomized trial. | 2014 |
| Gurewich (2011) | Comparative performance of community health centers and other usual sources of primary care. | 2011 |
| Healy-Collier (2016) | Medicaid managed care reduces readmissions for youths with type 1 diabetes. | 2016 |
| Hefei (2019) | Prescription Drug Monitoring Program Mandates: Impact On Opioid Prescribing And Related Hospital Use. | 2019 |
| Houck (2006) | Asthma prevention in urbanites. | 2006 |
| Karnick (2007) | The pediatric asthma intervention: A comprehensive cost-effective approach to asthma management in a disadvantaged inner-city community | 2007 |
| Kelley (2020) | PATIENT NAVIGATION TO REDUCE EMERGENCY DEPARTMENT (ED) UTILIZATION AMONG MEDICAID INSURED, FREQUENT ED USERS: A RANDOMIZED CONTROLLED TRIAL | 2020 |
| Kelly (2000) | Outcomes evaluation of a comprehensive intervention program for asthmatic children enrolled in Medicaid | 2000 |
| Kercsmar (2017) | Association of an Asthma Improvement Collaborative With Health Care Utilization in Medicaid-Insured Pediatric Patients in an Urban Community. | 2017 |
| Klinnert (2005) | Short-term impact of a randomized multifaceted intervention for wheezing infants in low-income families | 2005 |
| Lu (2011) | Association Between Prior Authorization for Medications and Health Service Use by Medicaid Patients With Bipolar Disorder | 2011 |
| Mackinney (2013) | Does providing care for uninsured patients decrease emergency room visits and hospitalizations?. | 2013 |
| Matone (2012) | Emergency department visits and hospitalizations for injuries among infants and children following statewide implementation of a home visitation model | 2012 |
| Murray (2007) | Pharmacist intervention to improve medication adherence in heart failure: a randomized trial | 2007 |
| Nelson (2011) | A randomized controlled trial of parental asthma coaching to improve outcomes among urban minority children. | 2011 |
| Nelson (2021) | Environmental Health Consults in Children Hospitalized with Respiratory Infections | 2021 |
| Ni (2017) | Impact of a pharmacy-based transitional care program on hospital readmissions. | 2017 |
| Rothkopf (2011) | Medicaid patients seen at federally qualified health centers use hospital services less than those seen by private providers. | 2011 |
| Saloner (2020) | Specialty Substance Use Disorder Treatment Admissions Steadily Increased In The Four Years After Medicaid Expansion. | 2020 |
| Scheeres (2020) | Changes in voluntary admission and restraint use after a comprehensive tobacco-free policy in inpatient psychiatric health facilities. | 2020 |
| Scott (2011) | Achieving and maintaining asthma control in inner-city children | 2011 |
| Shah (2011) | Evaluation of care management for the uninsured. | 2011 |
| Shepard (2002) | Managed care and the quality of substance abuse treatment. | 2002 |
| Smith (2004) | Improving follow-up for children with asthma after an acute emergency department visit | 2004 |
| Swaminathan (2020) | Association of Medicaid-Focused or Commercial Medicaid Managed Care Plan Type With Outpatient and Acute Care. | 2020 |
| Szilagyi (2000) | Evaluation of New York State's Child Health Plus: children who have asthma. | 2000 |
| Szilagyi (2000) | Evaluation of a state health insurance program for low-income children: Implications for State Child Health Insurance Programs | 2000 |
| Szilagyi (2006) | Improved asthma care after enrollment in the state children's health insurance program in New York | 2006 |
| Tabaei (2020) | Impact of a Telephonic Intervention to Improve Diabetes Control on Health Care Utilization and Cost for Adults in South Bronx, New York | 2020 |
| Unruh (2013) | Medicaid bed-hold policies and hospitalization of long-stay nursing home residents. | 2013 |
| Valdovinos (2020) | The association of Medicaid expansion and racial/ethnic inequities in access, treatment, and outcomes for patients with acute myocardial infarction | 2020 |
| Van Dorn (2010) | Continuing Medication and Hospitalization Outcomes After Assisted Outpatient Treatment in New York | 2010 |
| Vasan (2020) | Effects of a standardized community health worker intervention on hospitalization among disadvantaged patients with multiple chronic conditions: A pooled analysis of three clinical trials | 2020 |
| Vohra (2018) | Community health workers reduce hospitalizations and emergency department visits for low-socioeconomic urban patients with heart failure | 2018 |
| Wilson (2001) | A controlled trial of an environmental tobacco smoke reduction intervention in low-income children with asthma | 2001 |
| Woods (2012) | Community asthma initiative: evaluation of a quality improvement program for comprehensive asthma care. | 2012 |
| Wu (2009) | Asthma self-assessment in a Medicaid population. | 2009 |
| Zerzan (2007) | The demise of oregon's medically needy program: Effects of losing prescription drug coverage | 2007 |
| Zogg (2019) | Impact of Affordable Care Act Insurance Expansion on Pre-Hospital Access to Care: Changes in Adult Perforated Appendix Admission Rates after Medicaid Expansion and the Dependent Coverage Provision. | 2019 |
| Zolotor (2007) | Effectiveness of a practice-based, multimodal quality improvement intervention for gastroenteritis within a medicaid managed care network | 2007 |
| Castriotta (2020) | Measuring the impact of a social programme on healthcare: a 10-year retrospective cohort study in Trieste, Italy | 2020 |
| Downing (2019) | Impact of a community-based cardiovascular disease service intervention in a highly deprived area | 2019 |
| Horwitz (2021) | The effect of a coaching program on asthma control and health care utilization in children with asthma | 2021 |
| Hwang (2011) | Health status, quality of life, residential stability, substance use, and health care utilization among adults applying to a supportive housing program | 2011 |
| Somers (2013) | Vancouver At Home: pragmatic randomized trials investigating Housing First for homeless and mentally ill adults | 2013 |
| Ferrer (2013) | Advanced primary care in San Antonio: linking practice and community strategies to improve health - PubMed | 2013 |
| Bailey (2019) | Effect of Intensive Interdisciplinary Transitional Care for High-Need, High-Cost Patients on Quality, Outcomes, and Costs: a Quasi-Experimental Study | 2019 |
| Bartholomew (2006) | Partners in school asthma management: Evaluation of a self-management program for children with asthma | 2006 |
| Bauer (2012) | Leaving before discharge from a homeless Medical Respite program: predisposing factors and impact on selected outcomes. | 2012 |
| Burns (2014) | Feasibility and evaluation of a pilot community health worker intervention to reduce hospital readmissions. | 2014 |
| Caskey (2019) | Effect of Comprehensive Care Coordination on Medicaid Expenditures Compared With Usual Care Among Children and Youth With Chronic Disease: A Randomized Clinical Trial. | 2019 |
| Castillo (2018) | Improving Depression Care for Adults With Serious Mental Illness in Underresourced Areas: community Coalitions Versus Technical Support | 2018 |
| Chisolm (2020) | A Community Development Program and Reduction in High-Cost Health Care Use. | 2020 |
| Di Masso (2001) | The clubhouse model: an outcome study on attendance, work attainment and status, and hospitalization recidivism. | 2001 |
| Finkelstein (2020) | Health Care Hotspotting - A Randomized, Controlled Trial. | 2020 |
| Gary (2009) | The Effects of a Nurse Case Manager and a Community Health Worker Team on Diabetic Control, Emergency Department Visits, and Hospitalizations Among Urban African Americans With Type 2 Diabetes Mellitus A Randomized Controlled Trial | 2009 |
| Gill (2005) | Impact of providing a medical home to the uninsured: evaluation of a statewide program. | 2005 |
| Gomez (2017) | A Cost-Benefit Analysis of a State-Funded Healthy Homes Program for Residents With Asthma: Findings From the New York State Healthy Neighborhoods Program | 2017 |
| Liu (2018) | Transition Home Plus Program Reduces Medicaid Spending and Health Care Use for High-Risk Infants Admitted to the Neonatal Intensive Care Unit for 5 or More Days. | 2018 |
| Martinez (2006) | Impact of permanent supportive housing on the use of acute care health services by homeless adults | 2006 |
| Mehta (2017) | Community Partners in Care: 6-Month Outcomes of Two Quality Improvement Depression Care Interventions in Male Participants. | 2017 |
| Rose (2016) | A Population Health Approach to Clinical Social Work with Complex Patients in Primary Care. | 2016 |
| Rubin (2019) | Association of a Targeted Population Health Management Intervention with Hospital Admissions and Bed-Days for Medicaid-Enrolled Children. | 2019 |
| Sadowski (2009) | Effect of a Housing and Case Management Program on Emergency Department Visits and Hospitalizations Among Chronically Ill Homeless Adults A Randomized Trial | 2009 |
| Szanton (2018) | Medicaid Cost Savings of a Preventive Home Visit Program for Disabled Older Adults | 2018 |
| Tinland (2020) | Effectiveness of a housing support team intervention with a recovery-oriented approach on hospital and emergency department use by homeless people with severe mental illness: a randomised controlled trial | 2020 |
| Balaban (2020) | Using a Social Worker Transition Coach to Improve Hospital-to-Home Transitions for High-Risk Nonelderly Patients. | 2020 |
