# Supplementary material for: What works to reduce socioeconomic inequalities in hospitalisations and readmissions? Systematic review of the equity impacts of population-level, health service and integrative interventions
Source: BMJ Public Health. 2025 Sep 23;3(2):e002595. doi: 10.1136/bmjph-2025-002595 (PMC12458884; doi:10.1136/bmjph-2025-002595)
Supplement: online supplemental file 4 [file bmjph-3-2-s004.pdf]

Supplementary file 4: Table 1 – Study characteristics (N=36)

Author (year)	Country	Study type	Dataset/numbers of participants included in the analysis	Data collection timescale	Domain	Intervention	Measure of socioeconomic status the outcome is assessed differentially against	Measure of Outcome
1. Angraal (2018)[1]	USA	Cross-sectional time-series	NRD database: nationally representative data reported from 18-27 states during 2010-2015. The sample includes nearly half of all US hospitalisations each year. In 2015 the NRD represented 57.8% of the total population hospitalised in the US.	6 separate years (annual reporting 2010-2015)	Health service	Hospital Readmissions Reduction Program (established under Affordable Care Act): hospital performance financial penalty scheme based on three target conditions (Myocardial infarction (MI), heart failure (HF) and pneumonia).	Insurance type of patient (Medicare, Medicaid, private).	30 day readmission for three targeted conditions: MI, HF and pneumonia and a composite measure of non-intervention targeted conditions. Risk adjusted odds ratio for yearly change in readmissions.
2. Almquist (2022)[2]	Sweden	Quasi-experimental – regression discontinuity modelling, cross-sectional time-series	Longitudinal population and medical registries data covering total registered population in Sweden	2001-2012	Population health and public policy	2006 Swedish unemployment insurance reform – reducing level and coverage of unemployment insurance	Education level: low-educated (primary and secondary education only), high-educated (received tertiary education, eith longer vocational training or academic degrees)<br>Employment status: employed, unemployed.	Hospitalisation due to alcohol-related disorders

Author (year)	Country	Study type	Dataset/numbers of participants included in the analysis	Data collection timescale	Domain	Intervention	Measure of socioeconomic status the outcome is assessed differentially against	Measure of Outcome
3. Bell (2016)[3]	USA	RCT	851 adult participants (aged 18 and over) hospitalized for acute coronary syndrome or acute decompensated heart failure.	Participants enrolled May 2008 and September 2009.	Health service	Pharmacist led intervention immediately prior to discharge to educate patients on new medication regimes plus telephone follow-up.<br>Comparison: usual care.	Health literacy of patient.<br>The 36-item short form of the Test of Functional Health Literacy in Adults (s-TOFHLA)<br>Scores can range from 0 to 36, with higher scores indicating higher health literacy.<br>Scores grouped into ordered categorical 'inadequate, marginal and adequate' health literacy.	All adults hospitalised with acute coronary syndrome (ACS) or acute decompensated health failure (AHDF) within 30 days of intervention (readmissions).
4. Blanchard (2018)[4]	USA	Cohort	383,334 index hospitalisations involving opioid-related diagnoses across 13 sample states from the Agency for Healthcare Research and Quality Healthcare Cost and Utilization Project. Retrospective	April 2013 - June 2015	Health service	Three state policy approaches designed to provide opioid treatment options – note only intervention 3 reports sub-analysis of intervention effect by SES grouping:	Insurance type of patient: Medicare, Medicaid, private insurance, uninsured/self-pay, other	Within a 90-day period following discharge from an opioid related hospitalisation, readmission with an opioid-related principal diagnosis or an opioid-related accidental poisoning or ad-

Author (year)	Country	Study type	Dataset/numbers of participants included in the analysis	Data collection timescale	Domain	Intervention	Measure of socioeconomic status the outcome is assessed differentially against	Measure of Outcome
			longitudinal sample of patients aged 18 years and older with an opioid-related index hospitalisation and no preceding opioid-related hospitalisation within 90 days.			(1) expanded availability of naloxone to reverse overdose.<br>(2) Good Samaritan laws intended to protect individuals who attempt to provide or obtain emergency services for someone experiencing an opioid overdose.<br>(3) expanded coverage of medication-assisted treatment (MAT) for individuals with opioid abuse or dependence. Did not have the exact dates of implementation for Medicaid methadone or MAT coverage policies. Study		verse effect diagnosis.

Author (year)	Country	Study type	Dataset/numbers of participants included in the analysis	Data collection timescale	Domain	Intervention	Measure of socioeconomic status the outcome is assessed differentially against	Measure of Outcome
						relied on general implementation information for states for the range of years during study's data collection period.		
5. Capdevila (2023)[5]	Spain	Cohort	77 554 patients discharged alive with a diagnosis of HF: 3 396 were exposed to the HUB-DELTA (intervention) health care area and 74 158 were exposed to the rest of the health care areas in Catalonia.	2015-2019	Integrative	HUB-DELTA Heart Failure program - nurse-based multidisciplinary, transitional care HF program integrating hospital, primary care and community resources for patients	4 income categories according to individual annual income: an annual income > €100 000 was considered "high" income, €18 000 to 100 000 was considered "medium" income, and < €18 000 was considered "low" income. These 3 categories included both active workers as well as retired individuals receiving a retirement pension. Finally, individuals who received welfare support by the Government were considered to have	Admission to hospital with at least one ICD-9-CM code for HF as the primary diagnosis.

Author (year)	Country	Study type	Dataset/numbers of participants included in the analysis	Data collection timescale	Domain	Intervention	Measure of socioeconomic status the outcome is assessed differentially against	Measure of Outcome
							"very low income". For the analysis, patients with medium and high income were grouped into a single category.	
6. Cheon (2020)[6]	USA	Cohort	1979 hospitals. Linked dataset; data across four major datasets (AHA annual survey, IPPS, CMS Hospital Compare, and CHRN)	2015-2016	Integrative	Hospital-community partnerships existence and level of engagement in this intervention.	Safety-net hospital status	All cause hospital readmission within 30 days
7. Chou (2021)[7]	USA	Interrupted time-series	557,501 patients in HDHP intervention group, 5,861,990 in control group. Large commercial and Medicare advantage claims database. Adults who had at least 2 years of continuous enrolment in the database between 19 and	2003 - 2014	Health service	High-deductible health plan (HDHP)	Based on patient residential address. Neighbourhood poverty level - % of residents in neighbourhood living below the federal poverty level Lower-poverty neighbourhoods (<10%) Higher-poverty neighbourhoods (>=10%)	Hospital admission for non-specific chest pain as a result of the emergency department evaluation Hospital readmission for acute myocardial infarction

Author (year)	Country	Study type	Dataset/numbers of participants included in the analysis	Data collection timescale	Domain	Intervention	Measure of socioeconomic status the outcome is assessed differentially against	Measure of Outcome
			63 years old in the first year.					
8. Colla (2012)[8]	USA	Quasi-experimental	990,177 patients receiving PGPD intervention, 7,514,453 not receiving intervention.	Analysis compared preintervention (2001-2004) and postintervention (2005-2009)	Health service	Medicare Physician Group Practice Demonstration (PGPD) - participating physician groups received bonus payments if they achieved lower cost growth than local controls and met quality targets.	Dual registered (Medicare and Medicaid) versus single registered (Medicare). Dually eligible patients are 'overwhelmingly poor.'	30-day medical readmissions. 30-day surgical readmissions.
9. Connell (2020)[9]	USA	Interrupted time-series	4-17 year olds. 1874 encounters in the pre-ACA period and 2186 encounters in the post-ACA period. patients discharged from the inpatient psychiatric unit at a tertiary care, university-affiliated, 403-bed children's hospital with a dedicated paediatric ED in the	October 1, 2011, to September 30, 2016.	Health service	2014 Affordable Care Act (ACA) expansion of healthcare insurance provision.	Insurance type of patient: Medicaid or non-medicaid	Re-admission to hospital within 30 days of discharge from inpatient psychiatric unit across 24 months post ACA.

Author (year)	Country	Study type	Dataset/numbers of participants included in the analysis	Data collection timescale	Domain	Intervention	Measure of socioeconomic status the outcome is assessed differentially against	Measure of Outcome
			northwestern United States. As of 2019, there were 41 psychiatric unit beds available.					
10. DeWalt (2012)[10]	USA	RCT	605 patients with clinical diagnosis of heart failure recruited from general internal medicine and cardiology clinics	Recruitment 2007-2009, outcomes reported at 6 and 12 months	Health service	Self-care training single session vs multiple sessions	Literacy as measured by Short test of Functional Health Literacy in Adults	Incidence rate of heart failure related hospitalisation
11. Elmer (2014)[11]	UK	Cross-sectional (ecological)	Patients aged 0-19 admitted for dental extraction due to caries or disease	2006-2009	Population health and public policy	Water fluoridation – comparison of fluoridated/non-fluoridated Primary Care Trusts (PCT) areas across England	Index of multiple deprivation by PCTs	Directly standardised rates of admission for dental extraction by PCT
12. Garbutt (2015)[12]	USA	Cluster RCT	948 families with a child of 3-12 years with a diagnosis of asthma in a community-based primary care practice providing asthma	Recruitment March 2009 to May 2011, outcomes reported 12 and 24 months	Integrative	Telephone peer coaching for parents of children with asthma	Used Medicaid insurance as a surrogate for the low-income, high-risk group	Child asthma hospitalisations

Author (year)	Country	Study type	Dataset/numbers of participants included in the analysis	Data collection timescale	Domain	Intervention	Measure of socioeconomic status the outcome is assessed differentially against	Measure of Outcome
			care to 40 or more children					
13. Gosselin A (2016)[13]	Canada	Retrospective cohort	5033 vaccinated and 1239 unvaccinated children under 3yrs in post-universal vaccination period. Study also includes 6436 children from pre-universal period.	2008-2013	Health service	Rotavirus vaccine	The rate of low-income families (i.e. families having an annual income below the low-income cut-off), the unemployment rate among persons >=25 years of age, the rate of single mothers (i.e., not living with a partner) and the proportion of mothers without a high school diploma (i.e. <11 school years completed), derived from the National Census (2006) and the Live Births File (2002–2010) data, were measured at dissemination area (DA)-level. DA is the smallest geostatistical unit available from the census (approximately 400 to 700 persons by	Acute gastro-enteritis hospitalisations

Author (year)	Country	Study type	Dataset/numbers of participants included in the analysis	Data collection timescale	Domain	Intervention	Measure of socioeconomic status the outcome is assessed differentially against	Measure of Outcome
							DA). The 6-digit residential postal codes at birth, provided by CIRESSS, were geocoded in order to assign a DA to each participant (total of 512 DA). These ecological variables were then categorized in tertiles (T1, T2, T3), T3 representing the highest rate or proportion of poor socioeconomic indicators.	
14. Gosselin B (2016)[14]	Canada	Retrospective cohort	37,757 children born between June 1999 and May 2014 analysed at under 5 years	2004-2014	Health service	Rotavirus vaccine	Population density, low-income families, youth unemployment, single mothers, mothers without a high school diploma; categorised into 3 tertiles with T3 representing the most deprived	Hospitalisation rates due to acute gastroenteritis
15. Grotting (2020)[15]	Norway	Quasi-experimental	892,908 individuals in the registered	2007-2008	Population health and public policy	Statutory retirement age	Education level (higher vs lower)	All-cause hospitalisation

Author (year)	Country	Study type	Dataset/numbers of participants included in the analysis	Data collection timescale	Domain	Intervention	Measure of socioeconomic status the outcome is assessed differentially against	Measure of Outcome
			sample (all Norwegian adults) – this data used for hospitalisations. Also 4,619 individuals in the NorLAG sample (representative sample of Norwegian older adults)					
16. Herrtua (2015)[16]	Finland	Ecological/time series	People 30-79 years between 1 January 2000 and 31 December 2007 from the Statistics Finland Labour Market Data file	2001-2007	Population health and public policy	Average 33% tax reduction on alcohol in 2004 leading to reduction of minimal alcohol prices.	Educational level: upper tertiary, lower tertiary, secondary, basic	Alcohol related hospitalisations across 4 year period pre and 4 years post alcohol price reduction. Incidence rate ratio relative to upper tertiary education level.
17. Hungerford (2018)[17]	UK	Interrupted time-series	Hospital episode statistics, walk-in centre attendance records, GP records, NHS Trust records. Number not reported for AGE hospitalisations.	July 2004-June 2016	Health service	Rotavirus vaccine from July 2013	Deprivation quintile using Index of Multiple Deprivation	All-cause hospitalisation for acute gastroenteritis (AGE)

Author (year)	Country	Study type	Dataset/numbers of participants included in the analysis	Data collection timescale	Domain	Intervention	Measure of socioeconomic status the outcome is assessed differentially against	Measure of Outcome
18. Lu (2016)[18]	USA	Cross sectional time series	3395 hospitals	2013-2015	Health service	Hospital Readmission Reduction Programme (HRRP)	Safety net hospital; proportion of Medicaid or Medicare patients	30-day inpatient readmissions for pneumonia, acute myocardial infarction and heart failure
19. MacKay (2021)[19]	UK	Interrupted time-series	32,342 emergency admissions of children under 16 years	2000-2018	Population health and public policy	Smoke-free vehicle legislation	Scottish Index of Multiple Deprivation (SIMD)	Monthly incidence of hospital admission for exacerbation of asthma in children under the age of 16
20. Madden (2002)[20]	USA	Interrupted time-series	20,366 mother–infant pairs with normal vaginal deliveries	1990-1998	Health service	Reduced Obstetrical Length of Stay Programme (ROLOS – one night); minimum length of stay legislation (48 hours)	Medicaid, low income, low level of education	Hospital readmissions for newborns within the first 10 days of life
21. Meyers (2019)[21]	USA	Quasi-experimental	322,408 patients from 94 practices	2011-2015	Integrative	Reorganisation of clinical care and team building practices in primary care centres	Eligible for Medicaid	All cause hospitalisation and ambulatory care-sensitive hospitalisation

Author (year)	Country	Study type	Dataset/numbers of participants included in the analysis	Data collection timescale	Domain	Intervention	Measure of socioeconomic status the outcome is assessed differentially against	Measure of Outcome
22. Millett (2013)[22]	UK	Interrupted time-series	217,381 hospital admissions. All non-planned (emergency) hospital admissions for childhood asthma (aged 14 years old and under) in England from Hospital Episode Statistics (HES).	No. of admissions per day between April 1 2002 and November 30 2010	Population health and public policy	Comprehensive smoke-free legislation covering all enclosed public places and workplaces in England (July 2007).	Area-based measure: Quintiles of Index of Multiple Deprivation 2007 based on patient postcode.	Children (aged 14 and under) having emergency hospital admission with asthma 3 years post legislation introduction. Admission rate ratios: ratio of the actual admission rate in relation to the rate projected by the underlying trend. Estimated the number of admissions prevented in the 3 years after implementation of intervention by estimating number of admissions if there were no intervention (the counterfactual), then adding the differences in

Author (year)	Country	Study type	Dataset/numbers of participants included in the analysis	Data collection timescale	Domain	Intervention	Measure of socioeconomic status the outcome is assessed differentially against	Measure of Outcome
								numbers of admissions each month between the actual admissions and counterfactual estimate.
23. Murty (2016)[23]	USA	Retrospective cross sectional	141083 in-patient hospitalisations	2009-2011	Health service	Availability of primary care safety net clinics and health insurance status	Whether patients were uninsured	Preventable hospitalisations
24. Petousis (2019)[24]	New Zealand	Cohort	344 020 children from the 2006 census; 375 720 children from the 2013 census.	2006-2013	Health service	Pneumococcal conjugate vaccine	New Zealand Deprivation index	Hospitalisations for invasive pneumococcal disease, all-cause pneumonia, otitis media
25. Pimentel (2017)[25]	USA	Cross sectional time series	Annualised volume of 399,310 visits	2013-2015	Health service	Federal and state policy changes - clinical revision of Medicare waiver and creation of a global budget revenue model for acute care hospitals	Median income	Hospital admissions
26. Pressley (2009)[26]	USA	Cohort	37,246 children 3-8 years old	2003	Population health and public policy	Booster seat legislation	Median income	Motor vehicle occupant injury hospitalisations

Author (year)	Country	Study type	Dataset/numbers of participants included in the analysis	Data collection timescale	Domain	Intervention	Measure of socioeconomic status the outcome is assessed differentially against	Measure of Outcome
27. Piroddi (2022)[27]	UK	Quasi-experimental	Each Integrated care team services a population of 30,00 to 50,000, 19 teams covering the population of city of Liverpool (approx. 500,000 population)	Intervention: Jan 2017-Feb 2019	Integrative	Integrated Care Teams (ICTs): included new clinical care-coordinator role and staff from NHS, council social workers and voluntary sector professionals. Jointly funded service by NHS and local council.	Deprivation quintiles using English Index of Multiple Deprivation linked to patient record based on neighbourhood of residence.	Emergency admissions: non-elective admissions to hospital.
28. Rezansoff (2015)[28]	Canada	Cohort	A total of 631 individuals (aged 18 to 67) were available for inclusion in the analysis.	Participants were included if they were enrolled in the DTCV between its inception on December 4th, 2001 and March 31st, 2011. We restricted inclusion to individuals who exited the DTCV by March 31st, 2011, ensuring at least 12 months of follow-up data	Integrative	Drug Treatment Court participation (DTCV)	Individual measure: Education level Grade 9 or less, Grade 10/11, Grade 12, vocational/university	Acute hospital admission

Author (year)	Country	Study type	Dataset/numbers of participants included in the analysis	Data collection timescale	Domain	Intervention	Measure of socioeconomic status the outcome is assessed differentially against	Measure of Outcome
				for each participant.				
29. Rose (2021)[29]	UK	Quasi-experimental	108 intervention re and 540 control neighbourhoods were analysed over the period, giving a total sample size of 8424 neighbourhood-years	2005 to 2017	Population health and public policy	Declaring an Air Quality Management Area (AQMA) and Implementing a Local Air Quality Management system (LAQM). 47 AQMAs declared between 2006 and 2016 in the North West Coast of England.	Area-based measure: Three groupings; Least, middle and most income deprived areas from Index of Multiple Deprivation 2015	Emergency hospital admissions for respiratory conditions
30. Salerno (2017)[30]	USA	Interrupted Time Series	3254 US hospitals 52,516,213 index admissions. The initial sample included 55,313,070 index admissions at 5131 hospitals, eligible for the hospital-wide readmission measure. After exclusions, final cohort	January 2008 to June 2015	Health service	HRRP Hospital Readmission Reduction Program – part of ACA.<br><br>As part of the Affordable Care Act (ACA) in April 2010, the US Congress passed the Hospital Readmission Reduction Program	Safety net versus non-safety net hospitals. Safety net hospitals care for a larger proportion of patients with low socioeconomic status (SES). Among the 25% of hospitals defined as safety net hospitals using the AHRQ SES index, the mean percent of	Mean hospital-level, all-condition, 30-day risk-adjusted standardised unplanned readmission rate, measured quarterly, along with quarterly rate of change.

Author (year)	Country	Study type	Dataset/numbers of participants included in the analysis	Data collection timescale	Domain	Intervention	Measure of socioeconomic status the outcome is assessed differentially against	Measure of Outcome
			comprised 52,516,213 admissions at 3254 hospitals.			(HRRP), starting in October 2012, the Centres for Medicare and Medicaid Services (CMS) began Financially penalising hospitals that perform worse than the national average on risk standardised read-mission rates for Medicare patients.	patients with low SES served at safety net hospitals was 58.0% (15.3), whereas the mean percent was 17.1% (10.4) at non-safety net hospitals.	
31. Sankaran (2019)[31]	USA	Quasi-experimental	3238 acute care hospitals. Acute care hospitals were included if they had been assigned a total score for hospital acquired conditions in fiscal year 2015, were not located in Maryland or Puerto Rico, had	23 July 2014 and 30 November 2016.	Health service	Financial penalty Hospital Acquired Condition Reduction Program (HACRP) Part of ACA. Hospital receipt of a penalty in the first year of the HACRP.	Lowest versus highest quartile of disproportionate share hospitals (measuring socioeconomic disadvantage). Disproportionate Share Hospitals serve a significantly disproportionate number of low-income patients and receive payments	30 day readmission rate

Author (year)	Country	Study type	Dataset/numbers of participants included in the analysis	Data collection timescale	Domain	Intervention	Measure of socioeconomic status the outcome is assessed differentially against	Measure of Outcome
			data available from at least one American Hospital Association annual survey from 2012 to 2014, and had at least one eligible patient discharge during the study period.				from the Centres for Medicaid and Medicare Services to cover the costs of providing care to uninsured patients.	
32. Soto-Gordoa (2019)[32]	Spain	Cohort	The study included all individuals more than 65 years old with multimorbidity, which was defined as having two or more of three chronic diseases (DM, HF and COPD).4 The overall multimorbid population was 8239 patients in 2012 and 8364 patients in 2014.	2012-2014	Integrative	Integrated programme for multimorbid patients consisted of the deployment of multidisciplinary teams that included new roles such as the liaison nurse, case manager, advanced-skills nurse, reference internist, and an extensive infrastructure of information and communication technologies	Each study participant was assigned the socioeconomic deprivation index of his or her small area of residence. Afterwards, the socioeconomic deprivation index was divided into quintiles, with the first being the least disadvantaged and the fifth the most disadvantaged. However, in order to simplify interpretation, compared those in	All cause hospitalisation

Author (year)	Country	Study type	Dataset/numbers of participants included in the analysis	Data collection timescale	Domain	Intervention	Measure of socioeconomic status the outcome is assessed differentially against	Measure of Outcome
			Data were collected from the anonymized Basque Health Service corporate database.			based on the electronic health record (EHR) and electronic prescriptions. Moreover, patients had access to other programmes such as telehealth and patient empowerment.	the most deprived two quintiles (Q4–Q5) with the rest of the population (Q1–Q3).	
33. Turner (2020)[33]	Scotland – UK	Interrupted time-series	All hospital admissions in Scotland between 2000 and 2018 for children aged younger than 16 years.<br>740 055 total child admissions<br>32342 asthma admissions	Jan 1, 2000 to Dec 31, 2018	Population health and public policy	1: public health mass-media campaign – smoke free homes intervention<br>2: 2006 smoke free legislation	Scottish index of multiple deprivation. Those who live in the quintile of communities with greatest deprivation [SIMD 1] vs those who live in the intermediate [SIMD 3] and least deprived [SIMD 5] quintiles	Hospital admission for exacerbation of asthma in children aged under 16
34. Wharam (2018)[34]	USA	Interrupted time-series	Commercially insured members in the Optum database (Eden Prairie, MN).	1 January 2003 to 31 December 2012.	Health service	Intervention is being moved from a low to High-Deductable Insurance Plan (HDHP).	Area-based income measure.<br>Low income group: patient resides in a neighbourhood where 10% or more	All-cause hospitalisations, 12month period after switching to HDHP

Author (year)	Country	Study type	Dataset/numbers of participants included in the analysis	Data collection timescale	Domain	Intervention	Measure of socioeconomic status the outcome is assessed differentially against	Measure of Outcome
			23,493 HDHP members with diabetes, aged 12–64, insured through a large national health insurer from 2003 to 2012. HDHP members from low-income neighbourhoods (n = 8,453) were a subgroup of interest.			Intervention group: HDHP members were enrolled for 1 year in a low-deductible (less than or equal to $500) plan, followed by 1 year in an HDHP (more than or equal to $1,000 deductible) after an employer-mandated switch.<br>HDHPs require potential annual out-of-pocket payments of ;$1,000–$6,000 for most non-preventive services.<br><br>Control group: low-to-low deduction healthcare plan continuation among control group members was employer	of its residents are below the poverty level.<br>High income group: patient resides in a neighbourhood where less than 10% of its residents are below the poverty level.	(excluding birth hospitalisations). Direct hospital admissions – admissions not through the emergency department.

Author (year)	Country	Study type	Dataset/numbers of participants included in the analysis	Data collection timescale	Domain	Intervention	Measure of socioeconomic status the outcome is assessed differentially against	Measure of Outcome
						mandated, minimizing self-selection.		
35. Wyper (2023)[35]	Scotland - UK	Interrupted time series	Scottish population. Control group English population.	Data on hospitalisations before the legislation was implemented (1 Jan 2012 -30 April 2018) and 32 months thereafter (1 May 2019 - 31st December 2020).	Population health and public policy	Introducing minimum unit pricing for alcoholic drink sales	Deciles of the Scottish Index of Multiple Deprivation (intervention area) Deciles of the English Index of Multiple Deprivation (control area).	Hospitalisations for causes wholly attributable to alcohol consumption.
36. Zhao (2017)[36]	Canada	Cross-sectional time-series	All 89 Local Health Areas in British Columbia. Population data for each LHA used to calculate the quarterly rates of admissions per 100000 population. During study period 239, 022 alcohol-attributable admissions were estimated.	2022-2013 (48 quarters). Alcohol attributable hospitalisations from 2002 (start of minimum pricing) until 2013.	Population health and public policy	Increasing minimum unit pricing for alcoholic drink sales	Regional mean family income Low income (C$41,678-< 65,000) Medium income (C$65,000-<75,000) High income (C$75,000-180,434)	Estimated alcohol-attributable (AA) completed hospital admissions by applying population AA fractions to admission data for 60 categories or disease and injury. Percentage change in the rate of acute and chronic AA

Author (year)	Country	Study type	Dataset/numbers of participants included in the analysis	Data collection timescale	Domain	Intervention	Measure of socioeconomic status the outcome is assessed differentially against	Measure of Outcome
								hospital admissions 2002-2013. Percentage change in the rate of total chronic (100% and partial AA hospital admissions) 2022-2013 at quarterly lags of 1 year or more after price changes. Percentage change in the rate of chronic 100% AA hospital admissions 2022-2013 at quarterly lags of 1 year or more after price changes. Percentage change in the rate of chronic partial AA hospital admissions 2022-2013 at

Author (year)	Country	Study type	Dataset/numbers of participants included in the analysis	Data collection timescale	Domain	Intervention	Measure of socioeconomic status the outcome is assessed differentially against	Measure of Outcome
								quarterly lags of 1 year or more after price changes.

References – supplementary file 4: Table 1

1. Angraal S, Khera R, Zhou S, et al. Trends in 30-Day Readmission Rates for Medicare and Non-Medicare Patients in the Era of the Affordable Care Act. *The American Journal of Medicine* 2018;131(11):1324-31.e14. doi: 10.1016/j.amjmed.2018.06.013
2. Almquist YB, Miething A. The impact of an unemployment insurance reform on incidence rates of hospitalisation due to alcohol-related disorders: a quasi-experimental study of heterogeneous effects across ethnic background, educational level, employment status, and sex in Sweden. *BMC Public Health* 2022;22(1):1847. doi: 10.1186/s12889-022-14209-2
3. Bell SP, Schnipper JL, Goggins K, et al. Effect of Pharmacist Counseling Intervention on Health Care Utilization Following Hospital Discharge: A Randomized Control Trial. *Journal of General Internal Medicine* 2016;31(5):470-77. doi: 10.1007/s11606-016-3596-3
4. Blanchard J, Weiss AJ, Barrett ML, et al. State variation in opioid treatment policies and opioid-related hospital readmissions. *BMC Health Serv Res* 2018;18(1):971. doi: 10.1186/s12913-018-3703-8
5. Capdevila Aguilera C, Vela Vallespín E, Clèries Escayola M, et al. Population-based evaluation of the impact of socioeconomic status on clinical outcomes in patients with heart failure in integrated care settings. *Revista Española de Cardiología (English Edition)* 2023;76(10):803-12. doi: https://doi.org/10.1016/j.rec.2023.03.009
6. Cheon O, Baek J, Kash BA, et al. An exploration of community partnerships, safety-net hospitals, and readmission rates. *Health Services Research* 2020;55(4):531-40. doi: https://doi.org/10.1111/1475-6773.13287
7. Chou S-C, Hong AS, Weiner SG, et al. Impact of High-Deductible Health Plans on Emergency Department Patients With Nonspecific Chest Pain and Their Subsequent Care. *Circulation* 2021;144(5):336-49. doi: doi:10.1161/CIRCULATIONAHA.120.052501
8. Colla CH, Wennberg DE, Meara E, et al. Spending Differences Associated With the Medicare Physician Group Practice Demonstration. *JAMA* 2012;308(10):1015-23. doi: 10.1001/2012.jama.10812
9. Connell SK, Rutman LE, Whitlock KB, et al. Health Care Reform, Length of Stay, and Readmissions for Child Mental Health Hospitalizations. *Hospital Pediatrics* 2020;10(3):238-45. doi: 10.1542/hpeds.2019-0197
10. DeWalt DA, Schillinger D, Ruo B, et al. Multisite randomized trial of a single-session versus multisession literacy-sensitive self-care intervention for patients with heart failure. *Circulation* 2012;125(23):2854-62. doi: 10.1161/circulationaha.111.081745 [published Online First: 20120509]

11. Elmer TB, Langford JW, Morris AJ. An alternative marker for the effectiveness of water fluoridation: hospital extraction rates for dental decay, a two-region study. *Br Dent J* 2014;216(5):E10-E10. doi: 10.1038/sj.bdj.2014.180
12. Garbutt JM, Yan Y, Highstein G, et al. A cluster-randomized trial shows telephone peer coaching for parents reduces children's asthma morbidity. *Journal of Allergy and Clinical Immunology* 2015;135(5):1163-70.e2. doi: https://doi.org/10.1016/j.jaci.2014.09.033
13. Gosselin V, Généreux M, Gagneur A, et al. Effectiveness of rotavirus vaccine in preventing severe gastroenteritis in young children according to socioeconomic status. *Human Vaccines & Immunotherapeutics* 2016;12(10):2572-79. doi: 10.1080/21645515.2016.1189038
14. Gosselin V, Petit G, Gagneur A, et al. Trends in severe gastroenteritis among young children according to socio-economic characteristics before and after implementation of a rotavirus vaccination program in Quebec. *Canadian Journal of Public Health* 2016;107(2):e161-e67. doi: 10.17269/cjph.107.5286
15. Grøtting MW, Lillebø OS. Health effects of retirement: evidence from survey and register data. *Journal of Population Economics* 2020;33(2):671-704. doi: 10.1007/s00148-019-00742-9
16. Herttua K, Mäkelä P, Martikainen P. Educational inequalities in hospitalization attributable to alcohol: a population-based longitudinal study of changes during the period 2000–07. *Addiction* 2015;110(7):1092-100. doi: https://doi.org/10.1111/add.12933
17. Hungerford D, Vivancos R, Read JM, et al. Rotavirus vaccine impact and socioeconomic deprivation: an interrupted time-series analysis of gastrointestinal disease outcomes across primary and secondary care in the UK. *BMC Medicine* 2018;16(1):10. doi: 10.1186/s12916-017-0989-z
18. Lu N, Huang KC, Johnson JA. Reducing excess readmissions: promising effect of hospital readmissions reduction program in US hospitals. *Int J Qual Health Care* 2016;28(1):53-8. doi: 10.1093/intqhc/mzv090 [published Online First: 20151115]
19. Mackay DF, Turner SW, Semple SE, et al. Associations between smoke-free vehicle legislation and childhood admissions to hospital for asthma in Scotland: an interrupted time-series analysis of whole-population data. *Lancet Public Health* 2021;6(8):e579-e86. doi: 10.1016/s2468-2667(21)00129-8 [published Online First: 20210716]
20. Madden JM, Soumerai SB, Lieu TA, et al. Effects of a law against early postpartum discharge on newborn follow-up, adverse events, and HMO expenditures. *N Engl J Med* 2002;347(25):2031-8. doi: 10.1056/NEJMsa020408
21. Meyers DJ, Chien AT, Nguyen KH, et al. Association of Team-Based Primary Care With Health Care Utilization and Costs Among Chronically Ill Patients. *JAMA Internal Medicine* 2019;179(1):54-61. doi: 10.1001/jamainternmed.2018.5118
22. Millett C, Lee JT, Laverty AA, et al. Hospital Admissions for Childhood Asthma After Smoke-Free Legislation in England. *Pediatrics* 2013;131(2):e495-e501. doi: 10.1542/peds.2012-2592
23. Murty S, Begley CE, Franzini L, et al. Primary Care Availability, Safety Net Clinics, and Health Insurance Coverage: The Association of These Access Factors With Preventable Hospitalizations. *J Ambul Care Manage* 2016;39(3):253-63. doi: 10.1097/jac.0000000000000115
24. Petousis-Harris H, Howe AS, Paynter J, et al. Pneumococcal Conjugate Vaccines Turning the Tide on Inequity: A Retrospective Cohort Study of New Zealand Children Born 2006-2015. *Clin Infect Dis* 2019;68(5):818-26. doi: 10.1093/cid/ciy570
25. Pimentel L, Anderson D, Golden B, et al. Impact of Health Policy Changes on Emergency Medicine in Maryland Stratified by Socioeconomic Status. *West J Emerg Med* 2017;18(3):356-65. doi: 10.5811/westjem.2017.1.31778 [published Online First: 20170313]
26. Pressley JC, Trieu L, Barlow B, et al. Motor vehicle occupant injury and related hospital expenditures in children aged 3 years to 8 years covered versus uncovered by booster seat legislation. *J Trauma* 2009;67(1 Suppl):S20-9. doi: 10.1097/TA.0b013e3181951a90
27. Piroddi R, Downing J, Duckworth H, et al. The impact of an integrated care intervention on mortality and unplanned hospital admissions in a disadvantaged community in England: A difference-in-differences study. *Health Policy* 2022;126(6):549-57. doi: https://doi.org/10.1016/j.healthpol.2022.03.009
28. Rezansoff SN, Moniruzzaman A, Clark E, et al. Beyond recidivism: changes in health and social service involvement following exposure to drug treatment court. *Substance Abuse Treatment, Prevention, and Policy* 2015;10(1):42. doi: 10.1186/s13011-015-0038-x

29. Rose TC, Daras K, Cloke J, et al. Impact of local air quality management policies on emergency hospitalisations for respiratory conditions in the North West Coast region of England: a longitudinal controlled ecological study. *International Journal for Equity in Health* 2021;20(1):254. doi: 10.1186/s12939-021-01598-w
30. Salerno AM, Horwitz LI, Kwon JY, et al. Trends in readmission rates for safety net hospitals and non-safety net hospitals in the era of the US Hospital Readmission Reduction Program: a retrospective time series analysis using Medicare administrative claims data from 2008 to 2015. *BMJ Open* 2017;7(7):e016149. doi: 10.1136/bmjopen-2017-016149
31. Sankaran R, Sukul D, Nuliyalu U, et al. Changes in hospital safety following penalties in the US Hospital Acquired Condition Reduction Program: retrospective cohort study. *BMJ* 2019;366:l4109. doi: 10.1136/bmj.l4109
32. Soto-Gordoa M, Arrospide A, Millán E, et al. Gender and socioeconomic inequalities in the implementation of the Basque programme for multimorbid patients. *European Journal of Public Health* 2019;29(4):681-86. doi: 10.1093/eurpub/ckz071
33. Turner S, Mackay D, Dick S, et al. Associations between a smoke-free homes intervention and childhood admissions to hospital in Scotland: an interrupted time-series analysis of whole-population data. *The Lancet Public Health* 2020;5(9):e493-e500. doi: 10.1016/S2468-2667(20)30178-X
34. Wharam JF, Zhang F, Eggleston EM, et al. Effect of High-Deductible Insurance on High-Acuity Outcomes in Diabetes: A Natural Experiment for Translation in Diabetes (NEXT-D) Study. *Diabetes Care* 2018;41(5):940-48. doi: 10.2337/dc17-1183 [published Online First: 20180130]
35. Wyper GMA, Mackay DF, Fraser C, et al. Evaluating the impact of alcohol minimum unit pricing on deaths and hospitalisations in Scotland: a controlled interrupted time series study. *The Lancet* 2023;401(10385):1361-70. doi: 10.1016/S0140-6736(23)00497-X
36. Zhao J, Stockwell T. The impacts of minimum alcohol pricing on alcohol attributable morbidity in regions of British Colombia, Canada with low, medium and high mean family income. *Addiction* 2017;112(11):1942-51. doi: https://doi.org/10.1111/add.13902
