# Supplementary material for: What works to reduce socioeconomic inequalities in hospitalisations and readmissions? Systematic review of the equity impacts of population-level, health service and integrative interventions
Source: BMJ Public Health. 2025 Sep 23;3(2):e002595. doi: 10.1136/bmjph-2025-002595 (PMC12458884; doi:10.1136/bmjph-2025-002595)
Supplement: online supplemental file 5 [file bmjph-3-2-s005.pdf]

Supplementary file 5: Table 2 – Study outcomes (impact of interventions on socioeconomic inequalities in hospitalisations or readmissions)

Author (year)	Selected Quantitative findings	Further sub-analysis (e.g. by age, gender, ethnicity) following SES breakdown	Interpretation
Angraal (2018)[1]	Aged 65 years and more, readmission decreased annually:<br>• Acute myocardial infarction (risk-adjusted odds ratio OR [95% confidence interval] among Medicare patients, 0.94 [0.94-0.95], among Medicaid patients, 0.93 [0.90-0.97], and among patients with private-insurance, 0.95 [0.93-0.97]);<br>• Heart failure (ORs, 0.96 [0.96-0.97], 0.96 [0.940.98], and 0.97 [0.96-0.99], for the 3 payers, respectively),<br>• Pneumonia (ORs, 0.96 [0.96-0.97), 0.94 [0.92-0.96], and 0.96 [0.95-0.97], respectively).<br>Aged <65 years, readmission decreased annually:<br>• Acute myocardial infarction (ORs: Medicare 0.97 [0.96-0.98], Medicaid 0.94 [0.92-0.95], and private insurance 0.93 [0.92-0.94]),<br>• Heart failure (ORs, 0.98 [0.97-0.98]: 0.96 [0.96-0.97], and 0.97 [0.95-0.98], for the 3 payers, respectively),<br>• Pneumonia (ORs, 0.98 [0.97-0.99], 0.98 [0.97-0.99], and 0.98 [0.971.00], respectively).<br>Readmission rates also decreased significantly for non-target conditions for all insurance groups.	Yes by age. Compared readmission rates for those aged 65 years or more and those aged less than 65 years by insurance status (Medicare, Medicaid or private insurance).	Relative effect of intervention broadly consistent across Medicaid, medicare and private subgroups. Intervention will maintain the status quo of relative levels of inequality between the three insurance status groups.
Almquist (2022)[2]	Both the high education level group and the low education level group demonstrate a small decrease in incidence rates around the time of the unemployment insurance reform which reduced the level and coverage of unemployment insurance (low education: -0.47, p < 0.001; high education: -0.43, p < 0.001).<br>For the results stratified by employment status, there was a slight decrease in incidence rates for employed individuals (-0.56, p < 0.001) compared to a relatively sharp increase among unemployed in 2007 when the reform was implemented (1.04, p < 0.001).<br>Sub-analysis of the unemployed sub-population only stratified by education level (low or high). For the low-education sub-group, there is also a notable increase in incidence around the time of the reform (2.50, p < 0.001), but a decrease for high-educated individuals in the unemployed sub-group (-1.52, p < 0.001).	Unemployed sub-population stratified by education level (low or high).	Mixed.<br>No difference in the impact of the intervention according to education status (low or high). However, difference in impact of the intervention according to employment status (employed or unemployed).<br>Within the sub-population of unemployed, stratified by education. Those who were unemployed and low-education level there was an increase in hospitalisations at the time the intervention was implemented, but a decrease for the unemployed high-educated sub-group.

Author (year)	Selected Quantitative findings	Further sub-analysis (e.g. by age, gender, ethnicity) following SES breakdown	Interpretation
Bell (2016)[3]	Adjusted treatment effect of intervention – 30 day hospital admission hazard ratio HR [95% confidence interval]<br>Inadequate health literacy (n=87): HR 0.48 [0.15-1.48]<br>Marginal health literacy (n=76): HR 1.44 [0.40-5.15]<br>Adequate health literacy (n=688): HR 0.91 [0.61-1.35]	No	Intervention does not work to reduce the 30-day readmission outcome in any of the literacy groups. Therefore the intervention will maintain the current status quo with respect to socioeconomic inequalities in 30 day readmissions for acute coronary syndrome (ACS) or acute decompensated health failure (AHDF).
Blanchard (2018)[4]	By insurance group, odds ratio (OR) [95% Confidence Interval] of readmissions for patients in states with Medicaid methadone coverage versus states without:<br>Medicaid patients: OR 1.23 [0.97-1.56]<br>Medicare patients: OR 1.40 [1.15–1.72]<br>Privately insured patients: 0R 1.87 [1.44–2.41]<br>Uninsured patients: OR 1.57 [1.06–2.33]<br>Other insurance/missing: OR 0.99 [0.56-1.72]<br><br>By insurance group, odds ratio (OR) [95% Confidence Interval] of readmissions for patients in states with more generous Medicaid MAT coverage versus states with less generous Medicaid MAT coverage:<br>Medicaid patients: OR 1.28 [1.11–1.48]<br>Medicare patients: OR 0.80 [0.70–0.91]<br>Privately insured patients: OR 0.74 [0.63–0.85]<br>Uninsured patients: OR 1.20 [0.95-1.51]<br>Other insurance/missing: OR 1.14 [0.86-1.50]	No	Mixed.<br>This analysis showed associations of varying magnitude and significance, depending on insurance coverage, between opioid-related readmission and Medicaid coverage of methadone and Medicaid MAT coverage generosity.<br>Methadone coverage did not have a statistically significant association with readmission in the Medicaid population. However, in all other payer populations, individuals treated in states with Medicaid methadone coverage had higher odds of readmission.<br>Medicaid MAT coverage generosity was associated with lower odds of an opioid-related readmission among individuals covered by Medicare or private insurance but higher odds of readmission for those covered by Medicaid.
Capdevila (2023)[5]	The intervention appeared effective at reducing admissions across all SES groups.<br>The interaction term SES strata by health care area was not statistically significant in the consolidations period for clinically-related readmissions ([health care area*SES strata]:HR, 1.049; 95%CI, 0.806-1.366; P = .721), or for HF hospitalizations ([health care area*SES strata]: HR, 1.139, 95%CI, 0.808-1.605; P = .459).<br>However the effective size of the reduction in hospitalisations was greater among patients with medium/high SES.<br>In this stratum, the effect ranged from HR, 0.559; 95%CI, 0.345-0.904; P = .018 for clinically-related readmission to 0.407, 95%CI, 0.202-	No	Implementation of an intensive transitional care nurse-based HF management program integrating hospital, primary care, and community resources for patients with HF at a high risk of events translated into benefits in outcomes regardless of the SES of patients exposed to the program.<br>Increases inequalities: However, the benefit in terms of hospitalization was observed in all SES strata but the size effect was more prominent among patients with medium or high SES.

Author (year)	Selected Quantitative findings	Further sub-analysis (e.g. by age, gender, ethnicity) following SES breakdown	Interpretation
	0.819; P = .012 for HF readmission in 2019. On the other hand, size effects were substantially lower among the low or very low SES stratum: the best results were obtained in 2019 for clinically-related readmission HR in 2018 (HR, 0.861; 95%CI, 0.760-0.976; P = .019) and in 2018 for HF hospitalization (HR, 0.818; 95%CI, 0.702-0.954; P = .010).		
Cheon (2020)[6]	An increase in readmission rate is described: 0.537 (0.057) p< 0.001 for the effect on readmissions in safety-net hospitals from community partnerships, however when analysed for interaction between baseline characteristics and the effect of the intervention there was no evidence of true difference in the magnitude of the effect between the groups: Overall partnership × Safety net hospitals (D): 0.055 (0.030).	No	Maintain status quo. No significant reduction in disease-specific nor hospital-wide readmission at safety-net hospitals compared to non-safety net hospitals, despite their efforts to engage in community partnerships. Merely partnering with community organisations for population health improvement initiatives may not significantly reduce 30-day readmissions.
Chou (2021)[7]	When stratified by neighbourhood poverty rates,<br>Members living in higher-poverty neighbourhoods (>=10%) experienced reduction in index ED hospitalisations (–15.7% [95% CI, –19.2% to –12.3%]; absolute change, –2.8 per 10 000 person-years [95% CI, –3.4 to –2.1]<br>Members living in lower-poverty neighbourhoods (<10%) change in index ED hospitalisations (0.5% [95% CI, -4.5% to 5.6%]; absolute change, 0.06 per 10,000 person-years [95% CI, -0.5 to 07])<br><br>The increase in 30-day post-ED AMI admission among members living in neighbourhoods with higher poverty rates was statistically significant. The HDHP switch was associated with an increase of AMI hospitalisation in the 30 days after ED visit for nonspecific chest pain by 29.4% [95% CI, 13.3% to 45.6%]; absolute change, 0.6 per 10 000 person-years [95% CI, 0.3 to 0.9].<br><br>Members living in lower-poverty neighbourhoods (<10%) change in 30-day post-ED AMI admission: 6.9% [-20.7% to 34.5%], absolute change, 0.1 per 10 000 person-years [95% CI, -0.3 to 0.5]	No	Mixed impacts on socioeconomic inequalities. Reduces socioeconomic inequalities in index hospitalisations but increases inequalities in readmissions.<br><br>There was only a statistically significant reduction in index ED hospitalisations among members living in neighbourhoods with higher poverty areas. However, only members living in neighbourhoods with higher poverty rates experienced statistically significant increases in 30-day post-emergency department acute myocardial infarction admissions.

Author (year)	Selected Quantitative findings	Further sub-analysis (e.g. by age, gender, ethnicity) following SES breakdown	Interpretation
Colla (2012)[8]	Estimated change in 30 day medical readmission rate associated with PGPD intervention % [95% confidence interval]:<br>All patients: -0.67% [-1.11% to -0.23%]<br>Dually eligible (low socioeconomic status patients): -1.07% [-1.73% to -0.41%]<br>Non-dually eligible: -0.58% [-1.08% to -0.07%]<br><br>Estimated change in 30 day surgical readmission rate associated with PGPD intervention % [95% confidence interval]:<br>All patients: -0.17% [-0.59% to 0.25%]<br>Dually eligible (low socioeconomic status patients): -2.21% [-3.07% to -1.34%]<br>Non-dually eligible: 0.14% [-0.29% to 0.57%]<br>Surgical readmissions decreased only for the dully eligible (more disadvantaged socioeconomic status patients).	No	PGPD intervention reduces socioeconomic inequalities in readmissions.<br>Surgical readmissions decreased only for those in most socio-economically disadvantaged group (dually eligible) and medical readmissions also decreased in dually eligible.
Connell (2020)[9]	When stratified by insurance type, for those on Medicaid (more disadvantaged socio-economic status patients) observed a probable change in the slope direction (increasing pre-ACA to decreasing post-ACA) and a net decrease in the change in readmissions over time that did not reach statistical significance (pre-ACA versus post-ACA slope difference -0.4%; [95% CI -0.8% to 0.03%]). For those not on Medicaid, we saw the inverse, with a probable change in slope direction from decreasing pre-ACA to increasing post-ACA, and a net increase in the change in readmissions over time that did not reach statistical significance (pre-ACA versus post-ACA slope difference 0.2%; [95% CI -0.01% to 0.50%]).	No	Unclear impact of ACA intervention on socioeconomic inequalities in readmission for mental health conditions; converse trends may indicate a reduction in inequalities in readmissions but the trends did not reach statistical significance. There was an increase in 30 day inpatient readmissions for those initially admitted for mental health conditions overall and a non-significant increase in the average proportion of non-Medicaid patients readmitted, but a non-significant decrease for those on Medicaid. For those on Medicaid, the observed trajectory, if real, could potentially reflect better outpatient care, increased insurance denials, or a change in the covered population's illness severity.
DeWalt (2012)[10]	Overall, there was no difference in hospitalisations for heart failure patients receiving multi-session intervention compared to single session. However, the multisession intervention was more effective for those with low literacy.	No	Multi session intervention reduced inequalities: intervention decreased hospitalisation ratio for those with lower literacy rates.

Author (year)	Selected Quantitative findings	Further sub-analysis (e.g. by age, gender, ethnicity) following SES breakdown	Interpretation
	Adjusted incidence rate ratio for all patients hospitalisation<br>0.90 (0.70–1.15).<br>Adjusted incidence rate ratio (significant difference)<br>Inadequate literacy<br>0.48 (0.24–0.92)<br>Adequate literacy<br>1.34 (0.87–2.07)		
Elmer (2014)[11]	There was a statistically significant difference between the directly standardised rates (DSR) of admission of the PCTs in the West Midlands (mostly fluoridated, lower DSR) and the North West (mostly unfluoridated, higher DSR) for each year under examination. DSR for most deprived PCTS in West Midlands varied between 4.17 and 4.91 per 10,000; for similarly deprived areas in North West DSR varied between 51.51 and 112.58 per 10,000.	No	Reduces inequalities: Fluoridising water supplies reduces associated hospitalisations across the board but has greater effect on more disadvantaged socioeconomic groups.
Garbutt (2015)[12]	About 4 hours of peer training delivered by telephone over 12 months reduced asthma impairment for children from both the general and high-risk asthma populations. Medicaid subgroup had significant reductions in hospitalisations to 24mo, more so than other sources of insurance. Hospitalisations/child for Medicaid group at 12mo -0.16 (-0.30 to -0.01) Medicaid group at 24mo -0.11 (-0.22 to -0.004)	No	Reduces inequalities: Telephone delivered peer training reduces hospitalisations for low income, high risk asthma patients
Gosselin A (2016)[13]	Stratified analyses according to SES showed lower and non-significant vaccine effectiveness (VE) against acute gastro-enteritis (AGE) hospitalisation in most deprived subgroups (T3) as measured with rates of low-income families, unemployment and single mothers. This observation was particularly true for the analysis based on low-income family rates, as children from disadvantaged neighbourhoods (T3) had a twice lower VE (30% [95% CI: -40% -65%]) than those living in less deprived neighbourhoods (T1 and T2), who had a VE of 78% (95% CI: 52%-89%) (p = 0.027). For children living in neighbourhoods with higher rates of unemployment and single mothers, VE was also lower but not significantly different from other socioeconomic subgroups. On the other hand, children living in neighbourhoods with	No	Increases relative inequalities: rotavirus vaccine appears to be relatively less effective in preventing severe gastroenteritis among the most disadvantaged subgroups.

Author (year)	Selected Quantitative findings	Further sub-analysis (e.g. by age, gender, ethnicity) following SES breakdown	Interpretation
	a high proportion of low-educated mothers had higher VE than others, although these differences did not reach significance level.		
Gosselin B (2016)[14]	For socio-economic ecological-level variables, the most disadvantaged subgroups generally showed the lowest rate reductions in post-program years. This may have been caused by factors other than vaccination itself as no significant difference in vaccine coverage according to socio-economic subgroups was observed in the present study.<br>The lowest relative reductions in hospitalisations for acute gastroenteritis (AGE) were generally observed among the most urban and the most socio-economically disadvantaged areas (T3).<br>% rate reduction (95% CI):<br>Low-income family rate: T3 (high) 26 (4–42), T2 58 (43–69), T1 (low) 43 (23–58).<br>Unemployment rate: T3 (high) 35 (15-51), T2 45 (26-59), T1 (low) 46 (29-59)<br>Proportion of mothers without high school diploma rate: T3 (high) 50 (33-63), T2 26 (3-43), T1 (low) 50 (34-63)	No	Increases relative inequalities. The gap in hospitalisation rates from the least to the most deprived population tertiles increased post vaccine introduction whilst falling in absolute terms for both, specifically in terms of dividing SES groups by low income proportion.<br>This may have been caused by factors other than vaccination itself as no significant difference in vaccine coverage according to socio-economic subgroups was observed in the present study.
Grotting (2020)[15]	No effects of retirement on acute hospitalisations in general. However, results suggest that retirement leads to reduced likelihood of hospitalisations for individuals with low socioeconomic status. Retirement leads to a 0.6 percentage point reduction in the likelihood of acute hospitalisations for the low SES group. As the incidence of acute hospitalisations is 14%, this amounts to a 4% reduction in the likelihood of acute hospitalisations. The effect is significant at the 5% level. For the high SES group, we find an effect of 0.3, yet this is not significantly different from zero. Only suggestive evidence of a retirement effect on reduced likelihood of hospitalisations for the low SES group; the result does not pass robustness checks and must therefore be interpreted with caution.	No	Suggestive evidence of reducing inequalities in hospitalisations: the study reports a significant reduction in hospitalisation only in the lower SES group but this finding is not robust to sensitivity analyses.
Herrtua (2015)[16]	We observed a clear educational gradient among men and women both before and after the reduction in alcohol prices. We observed a relative increase in hospitalisations after the 2004 price reduction among men with a basic, secondary and lower tertiary education. Alcohol-attributable hospitalisations were unaffected by these price	Yes by gender	Increases inequalities: Reducing minimum alcohol prices increases hospitalisations related to alcohol along a gradient with the greatest impact on those in the lowest SES groups and no impact on those groups with the highest levels of education.

Author (year)	Selected Quantitative findings	Further sub-analysis (e.g. by age, gender, ethnicity) following SES breakdown	Interpretation
	changes among men with an upper tertiary education and among women, irrespective of the educational level. The greatest absolute increase in hospitalisation rates in lower levels of education.<br>Before price reduction, compared to upper tertiary level:<br>lower tertiary 1.66 (95% CI = 1.25, 2.19)<br>secondary 4.15 (95% CI = 3.20, 5.39)<br>basic 5.06 (95% CI = 3.88, 6.60)<br>After price reduction:<br>lower tertiary 2.15 (95% CI = 1.66, 2.80)<br>secondary 5.28 (95% CI = 4.14, 6.75)<br>basic 6.18 (95% CI = 4.82, 7.92)		
Hungerford (2018)[17]	Children aged < 12 months, number of all-cause AGE hospitalisations averted per 1,000 first-dose rotavirus vaccines delivered:<br>Most deprived populations, 2014/15<br>28 (95% CI 25–31)<br>Most deprived populations, 2015/16<br>26 (95% CI 23–30)<br>Least deprived populations, 2014/15<br>15 (95% CI 12–17)<br>Least deprived populations, 2015/16<br>13 (95% CI 11–16)<br><br>For children aged <12 months the rate of AGE hospitalisations averted per 1,000 first doses of vaccine was higher among infants in the most deprived communities compared to the least deprived in 2014/15 (28; 95% CI 25–31 vs. 15; 95% CI 12–17) and in 2015/16 (26; 95% CI 23–30 vs. 13; 95% CI 11–16).	Yes by age group (<12 months, 12-23 months)	Reduced inequalities. Vaccine impact was greatest among the most deprived populations, despite lower vaccine uptake.<br>Universal intervention benefitting all, but with increasing benefits of intervention across the social gradient.
Lu (2016)[18]	Stronger response of safety-net hospitals to the effect of HRRP on excess readmissions for AMI (P <0.05). However, the effect of HRRP on excess readmissions for PN and HF did not differ according to whether a hospital was safety-net hospital or not.	No	Mixed impact on inequalities: May lead to reductions in socioeconomic inequalities in readmissions (as measured by safety net status but not in terms of Medicaid status) in readmissions rates for acute myocardial infarction but not for pneumonia or heart failure.

Author (year)	Selected Quantitative findings	Further sub-analysis (e.g. by age, gender, ethnicity) following SES breakdown	Interpretation
	No evidence that the effect of HRRP was associated with the proportion of Medicare or Medicaid patients admitted to a hospital for any of the three conditions under study ($P > 0.05$).		
MacKay (2021)[19]	The relative reduction in monthly admissions to hospital was greatest among the most affluent quintile, and smaller but still significant in the middle quintile, but there was no significant change in the most deprived quintile:<br>SIMD 1 (most deprived): 0.14% (-1.42 to 1.72), p=0.86<br>SIMD 3: -1/72% (-3.23 to -0.18), p=0.028<br>SIMD 5 (least deprived): -2.27% (-4.41 to -0.07), p=0.042<br>However, the absolute reductions across quintiles were similar. Among the most deprived quintile, incidence fell from a mean of 0·87 admissions to hospital per 100, 000 population per month over the study period before the legislation to a mean of 0·72 admissions to hospital per 100 000 population per month over the months following the introduction of the legislation, and among the least deprived quintile from 0·32 per 100 000 population per month to 0·21 per 100 000 population per month, with an absolute reduction of 0·15 per 100 000 per month in both groups (95% CI 0·10–0·21 in the most deprived quintile and 0·11–0·18 in the least deprived quintile).	No	Increases relative inequality: no change in most deprived group with greater relative reduction in hospitalisations in least deprived group. Maintains absolute inequalities: absolute reduction in hospitalisation rates the same in both affluent and deprived groups.
Madden (2002)[20]	In the vulnerable subgroup, the overall rates of emergency department visits and rehospitalisation were 1.0 percent and 1.4 percent, respectively, but the numbers were too small to permit statistical time-series analysis.	No	Maintain status quo. Equally ineffective intervention at reducing newborn readmissions both overall and in the disadvantaged SES sub-group.
Meyers (2019)[21]	For full dataset (across all SES groups) In the chronically ill strata, we found clinically relevant reductions in hospitalisations and emergency department visits 2 years after a collaborative primary care transformation effort was initiated, and in a healthier strata, we found statistically significant increases in outpatient and hospital utilization.<br>However, in Medicaid only. No sig impact of intervention on full sample, or in the sub analysis of those with 2 or more chronic conditions.	Yes – sample with 2 or more chronic conditions	Mixed impact on inequalities. Overall the intervention increased hospitalisations in the group with less than two chronic conditions but decreased them in those with more than two chronic conditions. However, the intervention appeared ineffective for all of the Medicaid sub-group of patients, regardless of whether they had more or less than two chronic conditions.

Author (year)	Selected Quantitative findings	Further sub-analysis (e.g. by age, gender, ethnicity) following SES breakdown	Interpretation
	i.e. the statistically sig impacts found on full dataset were not found in the Medicaid sub analysis. So the intervention worked differently (not at all) in the Medicaid group unlike the full sample which included those across the socioeconomic spectrum and where it was seen as beneficial for comorbid chronically ill patients and detrimental in not chronically ill patients.<br><br>For Medicaid enrolees<br>Regression data:<br>Inpatient hospitalisations: AIC group Pre: 322.7, Post: 248.4<br>Comparison group Pre: 102.1, Post: 120.02<br>Inpatient hospitalisation Diff in Diff: 2.7<br><br>Effect as % of intervention baseline relative to comparisons 23% (p=0.9411) So not significant difference. I.e. the intervention didn't have an impact on changing the admissions rates for Medicaid patients.<br><br>Ambulatory sensitive hospitalisations: AIC Pre: 127.1, Post: 67.6<br>Comparison group Pre: 44.5, Post 34.0.<br>ACS hospitalisation Diff in Diff -11.3<br><br>Effect as % of intervention baseline relative to comparisons 19% (p=0.5283). Not significant difference. I.e. the intervention didn't have an impact on changing the admissions rates for Medicaid patients.<br><br>For a sub-set of patients with 2 or more chronic conditions who are Medicaid, there is still no significance impact of the intervention on outcome hospitalisations.		
Millett (2013)[22]	Hospital Admission rates for childhood asthma (aged 14 and under): Comparing the actual number of admissions with the counterfactual (predicted) admission rate had there been no intervention.	No	Intervention will maintain the status quo of relative levels of inequality across socioeconomic groups. Relative effect of intervention broadly consistent across IMD quintiles. Similar reductions in asthma

Author (year)	Selected Quantitative findings	Further sub-analysis (e.g. by age, gender, ethnicity) following SES breakdown	Interpretation
	Hospital admission rate ratios RR [95% Confidence interval] immediately following law:<br>Quintile 1 (least deprived): RR 0.94 [0.89-0.99]<br>Quintile 2: RR 0.91 [0.87-0.96]<br>Quintile 3: RR 0.86 [0.82-0.90]<br>Quintile 4: RR 0.92 [0.87-0.96]<br>Quintile 5 (most deprived): RR 0.93 [0.89-0.98]<br><br>Time after smoke-free law (per y) hospital admission rate ratios RR [95% Confidence interval]:<br>Quintile 1 (least deprived): RR 0.96 [0.93-0.98]<br>Quintile 2: RR 0.97 [0.95-0.99]<br>Quintile 3: RR 0.97 [0.95-0.99]<br>Quintile 4: RR 0.96 [0.94-0.98]<br>Quintile 5 (most deprived): RR 0.96 [0.94-0.98]		admissions among children from different socioeconomic status groups.
Murty (2016)[23]	An increase in safety net availability by 1 clinic in a 5-mile radius per 1000 population was associated with only a 4% reduction in odds of preventable hospitalisation in the insured, whereas the same level of expansion could lead to almost a 23% decline in preventable hospitalisation risk among the uninsured.	No	Taking uninsured status are a maker of low SES, safety net clinics appear to reduce inequalities because they have more impact on preventable hospitalisation risk in the uninsured relative to the insured group.
Petousis (2019)[24]	For IPD and otitis media, children in higher deprivation areas had higher rates of initial hospitalisations but the discrepancy lessened over time.<br>For pneumonia, hospitalisation rates reduced across all deprivation levels, no difference.<br>Figure 3 shows non-specific drop in inequality for initial invasive pneumococcal disease hospitalisation, no exact data provided.<br>Figure 5 (all cause pneumonia) shows no change in inequality, higher rates in more deprived groups maintained.<br>Figure 7 (otitis media) shows decrease in inequality but unclear without raw data.	No	Mixed evidence; for IPD and otitis media the vaccines decrease inequalities, for pneumonia maintains the status quo.

Author (year)	Selected Quantitative findings	Further sub-analysis (e.g. by age, gender, ethnicity) following SES breakdown	Interpretation
Pimentel (2017)[25]	The interaction of median catchment area income and ACA/GBR implementation was statistically significant in each model. The changes in each outcome are more pronounced for ED populations with lower median incomes.<br>Admission rates decreased the most at poorer hospitals, ranging from a decrease of 22% to no significant change.<br>Figure 5 - graph showing highest decrease in expected admittance rate for lowest SES households ranging to a small increase in highest SES households.	No	Reduces inequalities. Decreases in hospitalisations were more pronounced for emergency department populations with lower median incomes.
Pressley (2009)[26]	Covered (by booster legislation) children in all income categories demonstrated a tendency toward lower MV occupant injury/total all-cause injury than uncovered children, with larger differences noted for those residing in zip codes with incomes above the median.<br>Below median:<br>-13% ($p = 0.07$)<br>Above median:<br>-26% ($p = 0.001$)	No	Increases inequalities. There is a bigger difference in impact in areas with above-median income.
Piroddi (2022)[27]	Change in change of emergency admissions per 100 people per month [95% CI] of the intervention cohort relative to the control cohort:<br>Quintile 1 (least deprived): 20.2 [1.5-38.9] ($p<0.1$)<br>Quintile 2: 5.5 [-19.3-30.4] ($p=0.6$)<br>Quintile 3: 5.8 [-16.4-28.1] ($p=0.6$)<br>Quintile 4: 33.6 [8.9-58.2] ($p<0.01$)<br>Quintile 5 (most deprived): -1.2 [-25.0-22.5] ($p=0.9$)	No	Mixed.<br>The increase in emergency admission rate was highest among the 4th most deprived group, and to a lesser extent the least deprived group. The other groups experienced no significant effect.
Rezansoff (2015)[28]	Adjusted rate ratio [95% CI] by education level for post-treatment effect on acute hospital admissions<br>Grade 9 or less: 1.39 [0.72-2.68]<br>Grade 10/11: 0.97 [0.53-1.77]<br>Grade 12: 1.05 [0.55-2.00]	No	Maintains inequalities<br>No association across education groups in Drug Treatment Court participation benefit.
Rose (2021)[29]	There are statistically significant differential effects of declaring an AQMA across deprivation subgroups. In the least deprived subgroup, no statistically significant effect of declaring an AQMA. However, in the middle deprivation subgroup emergency admissions	No	Reduces inequalities.<br>There was a larger decrease in hospitalisation rates following the

Author (year)	Selected Quantitative findings	Further sub-analysis (e.g. by age, gender, ethnicity) following SES breakdown	Interpretation
	for respiratory conditions decreased in the intervention neighbourhoods by 184 per 100,000 per year [95% CI 68 to 301] after an AQMA was declared relative to the control neighbourhoods, and in the most deprived subgroup admissions decreased by 200 per 100,000 per year [95% CI 95 to 304].		declaration of an AQMA in more compared to less income deprived neighbourhoods.<br>Results suggest the LAQM system has contributed to a reduction in emergency hospitalisations for respiratory conditions, and may represent an effective strategy to reduce inequalities in health.
Salerno (2017)[30]	The mean safety net hospital standardised readmission rate declined from 17.0% (SD 3.7) to 13.6% (SD 3.6), whereas the mean non-safety net hospital declined from 15.4% (SD 3.0) to 12.7% (SD 2.5). The absolute difference in mean SRR (standardised readmission rate) between safety net and non-safety net hospitals declined from 1.6% (95% CI 1.3 to 1.9) in the first quarter of 2006 to 0.9% (0.7 to 1.2) in the second quarter of 2015, a 43% relative reduction in the gap between safety net hospitals and non-safety net hospitals. The quarterly decline in standardised readmission rates was 0.03 percentage points (95% CI 0.03 to 0.02, p<0.001) greater among safety net hospitals over the entire study period, and no differential change among safety net and non-safety net hospitals was found after either HRRP was passed or penalties enacted.	No	Intervention reduces socioeconomic inequalities in hospitalisations.<br><br>These results indicate that the gap in performance between safety net and non-safety net hospitals has narrowed over the years spanning HRRP's enactment and implementation.
Sankaran (2019)[31]	Rate of readmission at 30 days:<br>Lowest quarter DSH: -0.65 (-2.17 to 0.88)<br>Highest quarter DHS: -0.27 (-1.58 to 1.05)	No	Maintains status quo and prevailing inequalities.<br>Penalization intervention was not associated with a significant overall change in important clinical outcomes, including 30 day readmission.
Soto-Gordoa (2019)[32]	While the probability of in-hospitalisations decreased among men irrespective of their socioeconomic status (OR = 0.88, CI = 0.79–0.99 for the least deprived and OR = 0.79, CI = 0.70–0.89 for the most deprived), this effect was observed only in the most deprived women (OR = 0.85, CI = 0.73–0.98).<br><br>Odds ratios in hospitalisation between intervention (4225 in 2014) and control (3558 in 2012) groups<br>Less deprived (Q1, Q2, Q3)<br>Male: OR 0.88 [95% CI 0.79-0.99]	Yes - gender	Intervention reduces socioeconomic inequalities in hospitalisations.<br>This programme reduced in-hospitalisations by 2014 in all the selected patients, except for the least deprived women. The success was especially noteworthy among the most socioeconomically deprived men (OR= 0.79, CI= 0.70–0.89), who were the most disadvantaged group in 2012.

Author (year)	Selected Quantitative findings	Further sub-analysis (e.g. by age, gender, ethnicity) following SES breakdown	Interpretation
	Female: OR 1.23 [1.07-1.47]<br><br>Most deprived (Q4, Q5)<br>Male: OR 0.79 [0.70-0.89]<br>Female: OR 0.85 [0.73-0.98]		
Turner (2020)[33]	Smoke free legislation:<br>Following the 2006 legislation, independent of TiRO, asthma admissions decreased in children from the most deprived (SIMD 1; –0·49% [95% CI –0·87% to –0·11%], p=0·011) and intermediate deprived (SIMD 3; –0·70% [–1·17% to –0·23%], p=0·0043) area quintiles, but not in those from the least deprived (SIMD 5; -0.04% [-0.40% to 0.33%], p=0.85).<br><br>Asthma admissions did not change in any of the three deprivation categories after TiRO mass media campaign intervention in 2014.	No	Mixed impact on socioeconomic inequalities in asthma hospitalisations depending on which smoke-free intervention is considered. The smoke free legislation was associated with reduced inequalities in asthma hospitalisations but the mass-media campaign was not because asthma admissions did not change in any of the three deprivation categories after the Take it Right Out mass media campaign intervention in 2014.
Wharam (2018)[34]	Change in total hospitalisations in intervention (convert to HDHP) vs control group (remain LDHP):<br>Patients living in low-income neighbourhoods:<br>Relative % change (decrease) -5.7% [95% CI -9.9% to -1.5%]<br>Patients living in high-income neighbourhoods:<br>-5.4% [95% CI -12.1% to 1.2%]<br><br>Change in direct admissions (not via emergency department) per 1000 members:<br>Patients living in low-income neighbourhoods:<br>-10.0% [95% CI -14.8% to -5.3%]<br>Patients living in high-income neighbourhoods:<br>-7.7% [95% CI -14.2% to -1.2%]	No	Mixed impact on inequalities.<br>There is a statistically significant decrease in total hospitalisations in low-income neighbourhoods following the introduction of HDHP whereas the reduction in high-income neighbourhoods did not reach statistical significance.<br>In terms of direct hospital admissions (not via the emergency department) the HDHP intervention saw a decrease in % of hospitalisations for both the high and low income groups, the reduction being larger for patients living in low-income neighbourhoods.
Wyper (2023)[35]	Changes in hospitalisations [95% CI] wholly attributable to alcohol consumption associated with the implementation of alcohol minimum unit pricing (MUP) legislation:<br>Decile 1 (most deprived): -6.8% [-11.9 to -1.3]<br>Decile 2: -4.5% [-10.8 to 2.3]	No	Intervention of increasing minimum unit pricing of alcohol reduces socioeconomic inequalities.<br>The largest reductions in hospitalisations were estimated in the 40% most socioeconomically deprived areas in Scotland, indicating that the

Author (year)	Selected Quantitative findings	Further sub-analysis (e.g. by age, gender, ethnicity) following SES breakdown	Interpretation
	Decile 3: -6.3% [-11.3 to -1.0]<br>Decile 4: -6.9% [-11.4 to -2.3]<br>Decile 5: 11.9% [-0.5 to 25.7]<br>Decile 6: -0.7% [-9.8 to 9.2]<br>Decile 7: 0.7% [-7.6 to 9.7]<br>Decile 8: -1.2% [-8.1 to 6.4]<br>Decile 9: 0.3% [-8.3 to 9.7]<br>Decile 10 (least deprived): -2.0% [-16.8 to 15.5]		implementation of MUP has had a positive impact in tackling deprivation-based health inequalities in alcohol health harms.
Zhao (2017)[36]	1% increase in minimum prices was associated with rate of 100% AA hospital admissions as follows:<br>Regions with low average family income: an immediate and significant<br>3.547% decrease in admission rate [95% CI: -5.719 to -1.377]<br>Regions with medium average family income: 0.855% decrease in admission rate [-2.611 to +0.900]<br>Regions with high average family income: 0.557% decrease in admission rate [-2.517 to + 1.403]<br><br>A 1% increase in minimum prices was associated with a significant 2.518% reduction (t-test P < 0.001) in total AA hospital admissions (100% and partial) 2 years later in regions with low average annual family income and a significant 1.069% reduction in regions with high mean family income (t-test P < 0.001). No significant association was found in the regions with medium mean family income (t-test P > 0.05).<br><br>Multi-level regression analyses did not show a significant effect for the province as a whole when all the 89 LHAs were included. However, the analyses by regions with different income levels showed that a 1% increase in minimum prices was associated with a significant 2.242% reduction (95% CI: _4.097, _0.388; t-test P < 0.05) in chronic 100% AA hospital admissions 2 years later in the regions with low average annual family income, while no significant association was found in	No	Intervention of increasing minimum unit pricing of alcohol reduces socioeconomic inequalities. Minimum price increases for alcohol are associated with reductions in alcohol attributable hospitalisations, especially in regions with lower average family income, both for immediate effects on acute hospitalisations and delayed effects on chronic hospitalisations

Author (year)	Selected Quantitative findings	Further sub-analysis (e.g. by age, gender, ethnicity) following SES breakdown	Interpretation
	the regions with medium or high average annual family income (t-test P > 0.05 in each case).		

References supplementary file 5: table 2 – study outcomes

1. Angraal S, Khera R, Zhou S, et al. Trends in 30-Day Readmission Rates for Medicare and Non-Medicare Patients in the Era of the Affordable Care Act. *The American Journal of Medicine* 2018;131(11):1324-31.e14. doi: 10.1016/j.amjmed.2018.06.013
2. Almquist YB, Miething A. The impact of an unemployment insurance reform on incidence rates of hospitalisation due to alcohol-related disorders: a quasi-experimental study of heterogeneous effects across ethnic background, educational level, employment status, and sex in Sweden. *BMC Public Health* 2022;22(1):1847. doi: 10.1186/s12889-022-14209-2
3. Bell SP, Schnipper JL, Goggins K, et al. Effect of Pharmacist Counseling Intervention on Health Care Utilization Following Hospital Discharge: A Randomized Control Trial. *Journal of General Internal Medicine* 2016;31(5):470-77. doi: 10.1007/s11606-016-3596-3
4. Blanchard J, Weiss AJ, Barrett ML, et al. State variation in opioid treatment policies and opioid-related hospital readmissions. *BMC Health Serv Res* 2018;18(1):971. doi: 10.1186/s12913-018-3703-8
5. Capdevila Aguilera C, Vela Vallespín E, Clèries Escayola M, et al. Population-based evaluation of the impact of socioeconomic status on clinical outcomes in patients with heart failure in integrated care settings. *Revista Española de Cardiología (English Edition)* 2023;76(10):803-12. doi: https://doi.org/10.1016/j.rec.2023.03.009
6. Cheon O, Baek J, Kash BA, et al. An exploration of community partnerships, safety-net hospitals, and readmission rates. *Health Services Research* 2020;55(4):531-40. doi: https://doi.org/10.1111/1475-6773.13287
7. Chou S-C, Hong AS, Weiner SG, et al. Impact of High-Deductible Health Plans on Emergency Department Patients With Nonspecific Chest Pain and Their Subsequent Care. *Circulation* 2021;144(5):336-49. doi: doi:10.1161/CIRCULATIONAHA.120.052501
8. Colla CH, Wennberg DE, Meara E, et al. Spending Differences Associated With the Medicare Physician Group Practice Demonstration. *JAMA* 2012;308(10):1015-23. doi: 10.1001/2012.jama.10812
9. Connell SK, Rutman LE, Whitlock KB, et al. Health Care Reform, Length of Stay, and Readmissions for Child Mental Health Hospitalizations. *Hospital Pediatrics* 2020;10(3):238-45. doi: 10.1542/hpeds.2019-0197
10. DeWalt DA, Schillinger D, Ruo B, et al. Multisite randomized trial of a single-session versus multisession literacy-sensitive self-care intervention for patients with heart failure. *Circulation* 2012;125(23):2854-62. doi: 10.1161/circulationaha.111.081745 [published Online First: 20120509]
11. Elmer TB, Langford JW, Morris AJ. An alternative marker for the effectiveness of water fluoridation: hospital extraction rates for dental decay, a two-region study. *Br Dent J* 2014;216(5):E10-E10. doi: 10.1038/sj.bdj.2014.180
12. Garbutt JM, Yan Y, Highstein G, et al. A cluster-randomized trial shows telephone peer coaching for parents reduces children's asthma morbidity. *Journal of Allergy and Clinical Immunology* 2015;135(5):1163-70.e2. doi: https://doi.org/10.1016/j.jaci.2014.09.033

13. Gosselin V, Généreux M, Gagneur A, et al. Effectiveness of rotavirus vaccine in preventing severe gastroenteritis in young children according to socioeconomic status. *Human Vaccines & Immunotherapeutics* 2016;12(10):2572-79. doi: 10.1080/21645515.2016.1189038
14. Gosselin V, Petit G, Gagneur A, et al. Trends in severe gastroenteritis among young children according to socio-economic characteristics before and after implementation of a rotavirus vaccination program in Quebec. *Canadian Journal of Public Health* 2016;107(2):e161-e67. doi: 10.17269/cjph.107.5286
15. Grøtting MW, Lillebø OS. Health effects of retirement: evidence from survey and register data. *Journal of Population Economics* 2020;33(2):671-704. doi: 10.1007/s00148-019-00742-9
16. Herttua K, Mäkelä P, Martikainen P. Educational inequalities in hospitalization attributable to alcohol: a population-based longitudinal study of changes during the period 2000–07. *Addiction* 2015;110(7):1092-100. doi: https://doi.org/10.1111/add.12933
17. Hungerford D, Vivancos R, Read JM, et al. Rotavirus vaccine impact and socioeconomic deprivation: an interrupted time-series analysis of gastrointestinal disease outcomes across primary and secondary care in the UK. *BMC Medicine* 2018;16(1):10. doi: 10.1186/s12916-017-0989-z
18. Lu N, Huang KC, Johnson JA. Reducing excess readmissions: promising effect of hospital readmissions reduction program in US hospitals. *Int J Qual Health Care* 2016;28(1):53-8. doi: 10.1093/intqhc/mzv090 [published Online First: 20151115]
19. Mackay DF, Turner SW, Semple SE, et al. Associations between smoke-free vehicle legislation and childhood admissions to hospital for asthma in Scotland: an interrupted time-series analysis of whole-population data. *Lancet Public Health* 2021;6(8):e579-e86. doi: 10.1016/s2468-2667(21)00129-8 [published Online First: 20210716]
20. Madden JM, Soumerai SB, Lieu TA, et al. Effects of a law against early postpartum discharge on newborn follow-up, adverse events, and HMO expenditures. *N Engl J Med* 2002;347(25):2031-8. doi: 10.1056/NEJMsa020408
21. Meyers DJ, Chien AT, Nguyen KH, et al. Association of Team-Based Primary Care With Health Care Utilization and Costs Among Chronically Ill Patients. *JAMA Internal Medicine* 2019;179(1):54-61. doi: 10.1001/jamainternmed.2018.5118
22. Millett C, Lee JT, Laverty AA, et al. Hospital Admissions for Childhood Asthma After Smoke-Free Legislation in England. *Pediatrics* 2013;131(2):e495-e501. doi: 10.1542/peds.2012-2592
23. Murty S, Begley CE, Franzini L, et al. Primary Care Availability, Safety Net Clinics, and Health Insurance Coverage: The Association of These Access Factors With Preventable Hospitalizations. *J Ambul Care Manage* 2016;39(3):253-63. doi: 10.1097/jac.0000000000000115
24. Petousis-Harris H, Howe AS, Paynter J, et al. Pneumococcal Conjugate Vaccines Turning the Tide on Inequity: A Retrospective Cohort Study of New Zealand Children Born 2006-2015. *Clin Infect Dis* 2019;68(5):818-26. doi: 10.1093/cid/ciy570
25. Pimentel L, Anderson D, Golden B, et al. Impact of Health Policy Changes on Emergency Medicine in Maryland Stratified by Socioeconomic Status. *West J Emerg Med* 2017;18(3):356-65. doi: 10.5811/westjem.2017.1.31778 [published Online First: 20170313]
26. Pressley JC, Trieu L, Barlow B, et al. Motor vehicle occupant injury and related hospital expenditures in children aged 3 years to 8 years covered versus uncovered by booster seat legislation. *J Trauma* 2009;67(1 Suppl):S20-9. doi: 10.1097/TA.0b013e3181951a90
27. Piroddi R, Downing J, Duckworth H, et al. The impact of an integrated care intervention on mortality and unplanned hospital admissions in a disadvantaged community in England: A difference-in-differences study. *Health Policy* 2022;126(6):549-57. doi: https://doi.org/10.1016/j.healthpol.2022.03.009
28. Rezansoff SN, Moniruzzaman A, Clark E, et al. Beyond recidivism: changes in health and social service involvement following exposure to drug treatment court. *Substance Abuse Treatment, Prevention, and Policy* 2015;10(1):42. doi: 10.1186/s13011-015-0038-x
29. Rose TC, Daras K, Cloke J, et al. Impact of local air quality management policies on emergency hospitalisations for respiratory conditions in the North West Coast region of England: a longitudinal controlled ecological study. *International Journal for Equity in Health* 2021;20(1):254. doi: 10.1186/s12939-021-01598-w
30. Salerno AM, Horwitz LI, Kwon JY, et al. Trends in readmission rates for safety net hospitals and non-safety net hospitals in the era of the US Hospital Readmission Reduction Program: a retrospective time series analysis using Medicare administrative claims data from 2008 to 2015. *BMJ Open* 2017;7(7):e016149. doi: 10.1136/bmjopen-2017-016149

31. Sankaran R, Sukul D, Nuliyalu U, et al. Changes in hospital safety following penalties in the US Hospital Acquired Condition Reduction Program: retrospective cohort study. *BMJ* 2019;366:l4109. doi: 10.1136/bmj.l4109
32. Soto-Gordoa M, Arrospide A, Millán E, et al. Gender and socioeconomic inequalities in the implementation of the Basque programme for multimorbid patients. *European Journal of Public Health* 2019;29(4):681-86. doi: 10.1093/eurpub/ckz071
33. Turner S, Mackay D, Dick S, et al. Associations between a smoke-free homes intervention and childhood admissions to hospital in Scotland: an interrupted time-series analysis of whole-population data. *The Lancet Public Health* 2020;5(9):e493-e500. doi: 10.1016/S2468-2667(20)30178-X
34. Wharam JF, Zhang F, Eggleston EM, et al. Effect of High-Deductible Insurance on High-Acuity Outcomes in Diabetes: A Natural Experiment for Translation in Diabetes (NEXT-D) Study. *Diabetes Care* 2018;41(5):940-48. doi: 10.2337/dc17-1183 [published Online First: 20180130]
35. Wyper GMA, Mackay DF, Fraser C, et al. Evaluating the impact of alcohol minimum unit pricing on deaths and hospitalisations in Scotland: a controlled interrupted time series study. *The Lancet* 2023;401(10385):1361-70. doi: 10.1016/S0140-6736(23)00497-X
36. Zhao J, Stockwell T. The impacts of minimum alcohol pricing on alcohol attributable morbidity in regions of British Colombia, Canada with low, medium and high mean family income. *Addiction* 2017;112(11):1942-51. doi: https://doi.org/10.1111/add.13902
